# Synthesis, comprehensive in silico studies, and cytotoxicity evaluation of novel quinazolinone derivatives as potential anticancer agents

**DOI:** 10.1038/s41598-025-08062-7

**Published:** 2025-07-03

**Authors:** David S. A. Haneen, Abdelaal A. Abdalha, Musaab M. Alkhatib, Mahmoud Kamal, Ahmed S. A. Youssef, Wael S. I. Abou-Elmagd, Sandy S. Samir

**Affiliations:** 1 Chemistry Department, Faculty of Science, Ain Shams University, Abbassia, Cairo, 11566 Egypt; 2 Entomology Department, Faculty of Science, Ain Shams University, Cairo, 11566 Egypt

**Keywords:** Quinazolinone, Benzoxazinone, Hydrazide derivatives, Cytotoxicity, Molecular docking, Anticancer agents, Computational studies, Cancer, Computational biology and bioinformatics, Chemistry

## Abstract

Quinazolinone derivatives exhibit significant synthetic and biological properties, making them valuable candidates for anticancer drug development. In this study, we synthesized novel quinazolinone derivatives via reactions of the previously unreported 4*H*-benzoxazin-4-one derivative (**2**) with various nitrogen nucleophiles, such as hydrazine hydrate and ammonium acetate, which yielded hydrazide and quinazolinone derivatives (**3–6**). Further modifications were achieved through reactions with carbon electrophiles and carbonyl compounds (e.g., phthalic anhydride, phenyl isothiocyanate, potassium cyanate, and pyrazole carbaldehyde), resulting in an expanded library of novel quinazolinone derivatives (**7–15**). The structures of all synthesized compounds were confirmed by spectroscopic and microanalytical techniques. In vitro cytotoxicity evaluations (mean IC_50_ ± SE, *n* = 3) revealed promising anticancer activity, with compounds **3** and **5** demonstrating the highest efficacy. To gain deeper mechanistic insights, molecular docking and molecular dynamics simulations were performed against key cancer-related targets, including Topoisomerase II, VEGFR2, c-Met, EGFR, and Estrogen Receptor Alpha. The combined experimental and computational findings suggest that these quinazolinone derivatives hold significant potential as novel anticancer agents, justifying further biological investigations and structural optimizations.

**Supplementary Information:**

The online version contains supplementary material available at 10.1038/s41598-025-08062-7.

## Introduction

Heterocyclic compounds are crucial in medicinal chemistry due to their biologically active nature. Quinazolines are significant heterocycles in therapeutic chemistry^1–3^, with quinazoline derivatives like quinazolinone offering diverse biological properties^4–8^, making them important nitrogen-containing heterocycles. Quinazolinone derivatives, particularly 4(3*H*)-quinazolinone, have several biological uses, like antibacterial and antifungal^9–12^, anti-HIV^13^, antimalarial^14^, antiviral^15^, anti-inflammatory^16^, anticancer^17–19^, analgesic, anticonvulsant, CNS depressant, antioxidant, and anti-leukemic activities^20–22^.

Conventional methods for synthesizing 4(3*H*)-quinazolinones include combining 2-aminobenzoic acid with acyl chloride or acid anhydride, resulting in benzoxazinone, which is subsequently condensed with an amine for the desired product^23,24^. Since these moieties serve as valuable intermediates in organic synthesis^20^, there is growing interest in the chemistry of this system, and a variety of synthetic methods have been developed^25–27^.

**Fig. 1:**
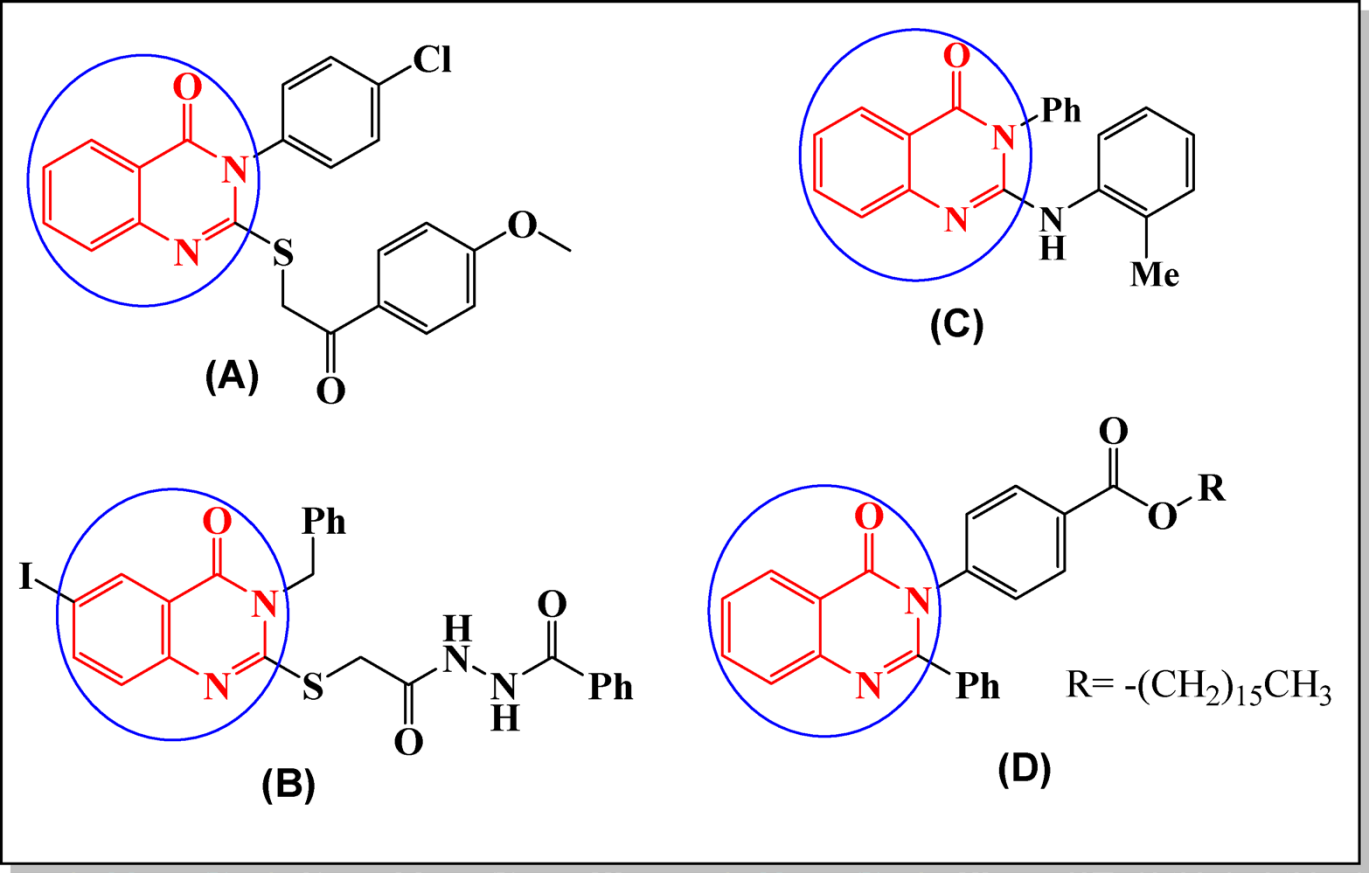
Quinazolinone derivatives which are used as anti-tumor agents.

Quinazolinone derivatives **(A-D)** have been widely used as anti-tumor agents, as illustrated in Fig. 1. These derivatives also contain a variety of functional groups such as amidic, hydrazine, benzoyl, and phenyl rings. They showed antitumor activity against various cell lines, such as 3-aryl-2-substituted mercapto-quinazolin-4(3*H*)-one **(A)**^28^, 3-benzylquinazolin-4(3*H*)-one derivative **(B)** containing 6-Iodo with 2-arylthio function^29^, the 3-phenyl-2-(o-tolylamino)quinazolin-4(3*H*)-one **(C)**^30^, 4-oxo-2-phenyl-3(4*H*)-quinazoline derivative^31^. In the present study, we aimed to synthesize new quinazoline-4(3*H*)-one derivatives containing specific target groups derived from a benzoxazinone derivative and investigate their anticancer activities.

Cancer is a complex and multifaceted disease driven by dysregulated molecular pathways that promote uncontrolled proliferation, resistance to apoptosis, angiogenesis, and metastasis^32^. Several key receptors play pivotal roles in these processes, making them critical targets for anticancer drug development. Topoisomerase II is an essential enzyme involved in DNA replication, transcription, and chromosome segregation^33–35^. Overexpression of Topoisomerase II in cancer cells leads to enhanced DNA replication and repair, allowing tumors to proliferate rapidly. Inhibiting this enzyme disrupts DNA topology, causing DNA strand breaks and apoptosis, making it a validated target for chemotherapeutic agents like doxorubicin^33–35^. Another major target, vascular endothelial growth factor receptor 2 (VEGFR2), is a key mediator of tumor angiogenesis, the process by which cancer cells stimulate new blood vessel formation to ensure an adequate supply of oxygen and nutrients^36–40^. VEGFR2 overexpression is strongly associated with tumor progression and metastasis, particularly in aggressive cancers such as hepatocellular carcinoma and breast cancer. Inhibiting VEGFR2 blocks angiogenesis, thereby depriving tumors of their essential blood supply and limiting their growth^36–40^.

In addition to promoting angiogenesis, c-Met (hepatocyte growth factor receptor, HGFR) plays a crucial role in tumor invasion, metastasis, and resistance to apoptosis. c-Met activation leads to enhanced cell motility, epithelial-to-mesenchymal transition (EMT), and metastatic dissemination, making it a key target in cancers that exhibit high metastatic potential, including breast and liver cancers. Inhibition of c-Met disrupts these oncogenic pathways, preventing tumor spread and increasing cancer cell susceptibility to apoptosis^41–45^. Similarly, epidermal growth factor receptor (EGFR) is a well-characterized driver of tumor cell proliferation and survival. EGFR overactivation is frequently observed in breast, lung, and liver cancers, where it triggers downstream signaling cascades such as the RAS–RAF–MEK–ERK and PI3K–AKT pathways, leading to enhanced cell division and survival. Targeting EGFR with tyrosine kinase inhibitors (TKIs) like gefitinib effectively blocks these signaling pathways, reducing tumor growth and increasing cancer cell sensitivity to chemotherapy^46–48^.

Lastly, estrogen receptor alpha (ERα) is a key regulator of hormone-dependent cancers, particularly breast cancer, where it drives tumor proliferation in response to estrogen signaling. Overexpression of ERα is a hallmark of estrogen receptor-positive (ER+) breast cancers, making it a crucial target for endocrine therapy using selective estrogen receptor modulators (SERMs) like tamoxifen. Inhibiting ERα disrupts hormone-driven tumor growth, improving patient outcomes and reducing the risk of cancer recurrence^49–52^. Given the essential roles of these receptors in cancer progression, targeting them simultaneously through multi-target inhibitors or combination therapies holds significant promise for improving treatment efficacy and overcoming drug resistance.

In this study, we synthesized novel quinazolinone derivatives from benzoxazinone precursors and evaluated their anticancer potential. To complement the experimental cytotoxicity screening, we conducted in silico molecular docking studies to predict the binding interactions of these derivatives with the key cancer-related targets, including Topoisomerase II, VEGFR2, c-Met, EGFR, and ERα. Additionally, molecular dynamics (MD) simulations were performed to validate the docking results and assess the stability and dynamic behavior of the most promising ligand-receptor complexes over a 100 ns trajectory. The integration of experimental and computational approaches provides valuable insights into the potential of these quinazolinone derivatives as anticancer agents, laying a rational foundation for further structural optimization and biological evaluation.

## Results and discussion

**Fig. 2:**
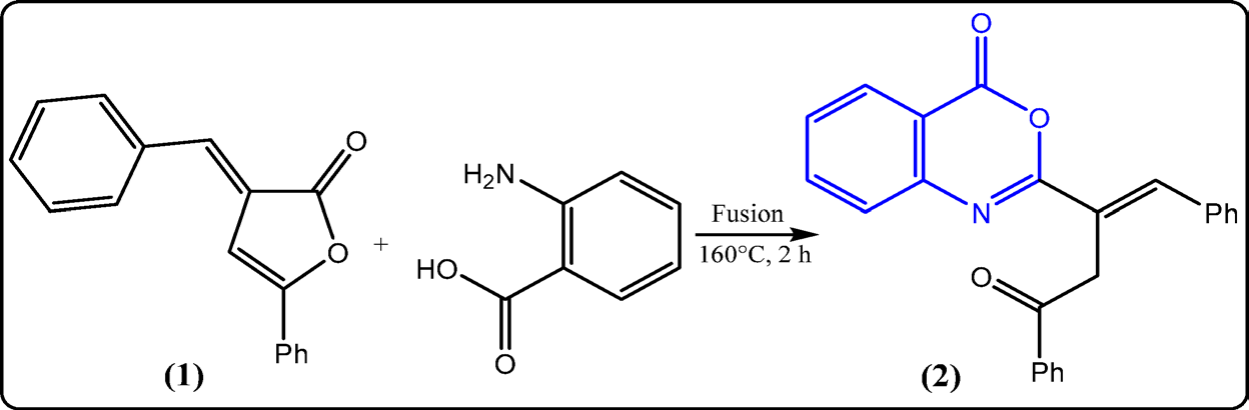
Synthesis of 4-benzoxazinone derivative **2**.

**Fig. 3:**
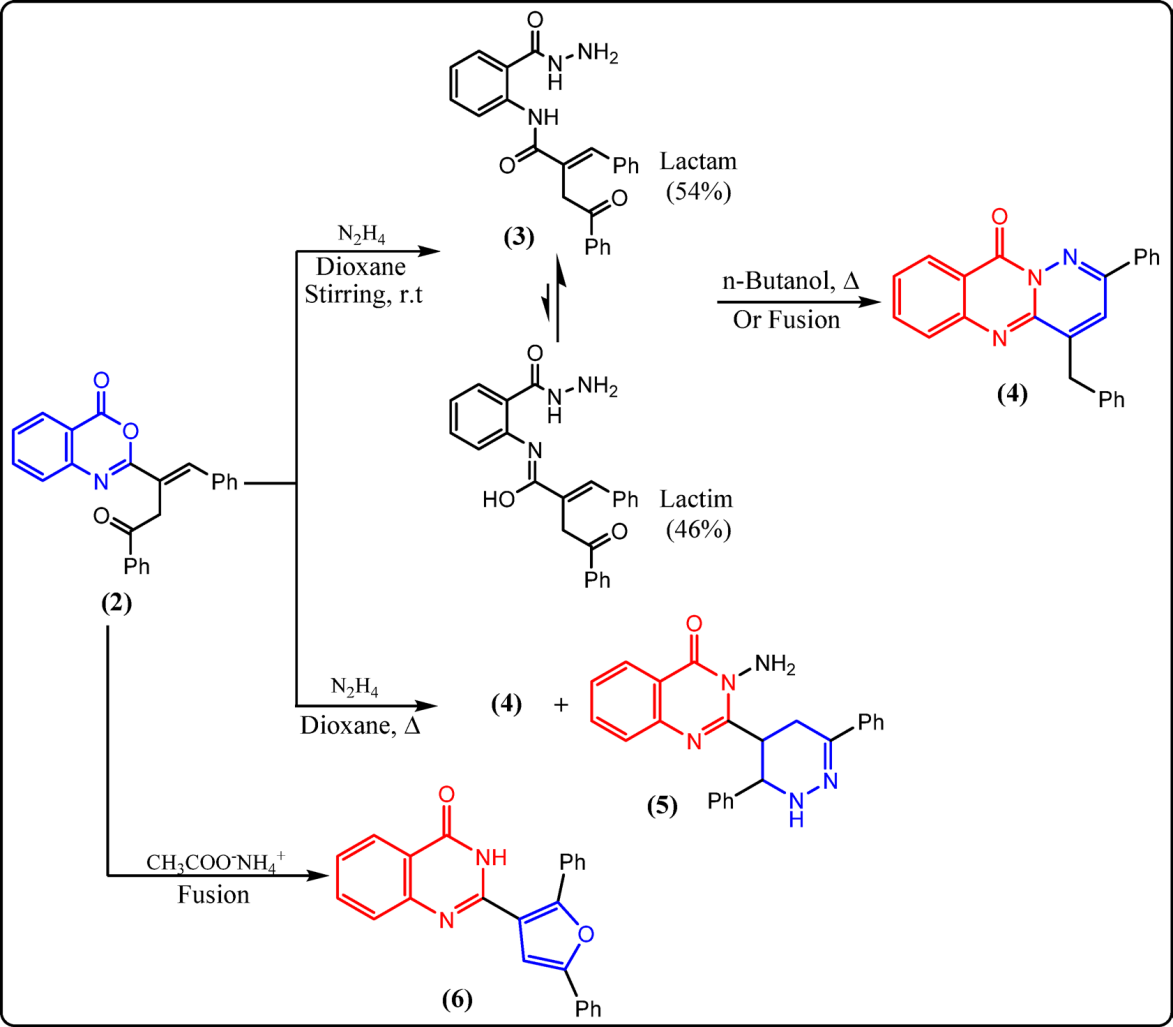
Reactions of **2** with nitrogen nucleophiles.

**Fig. 4:**
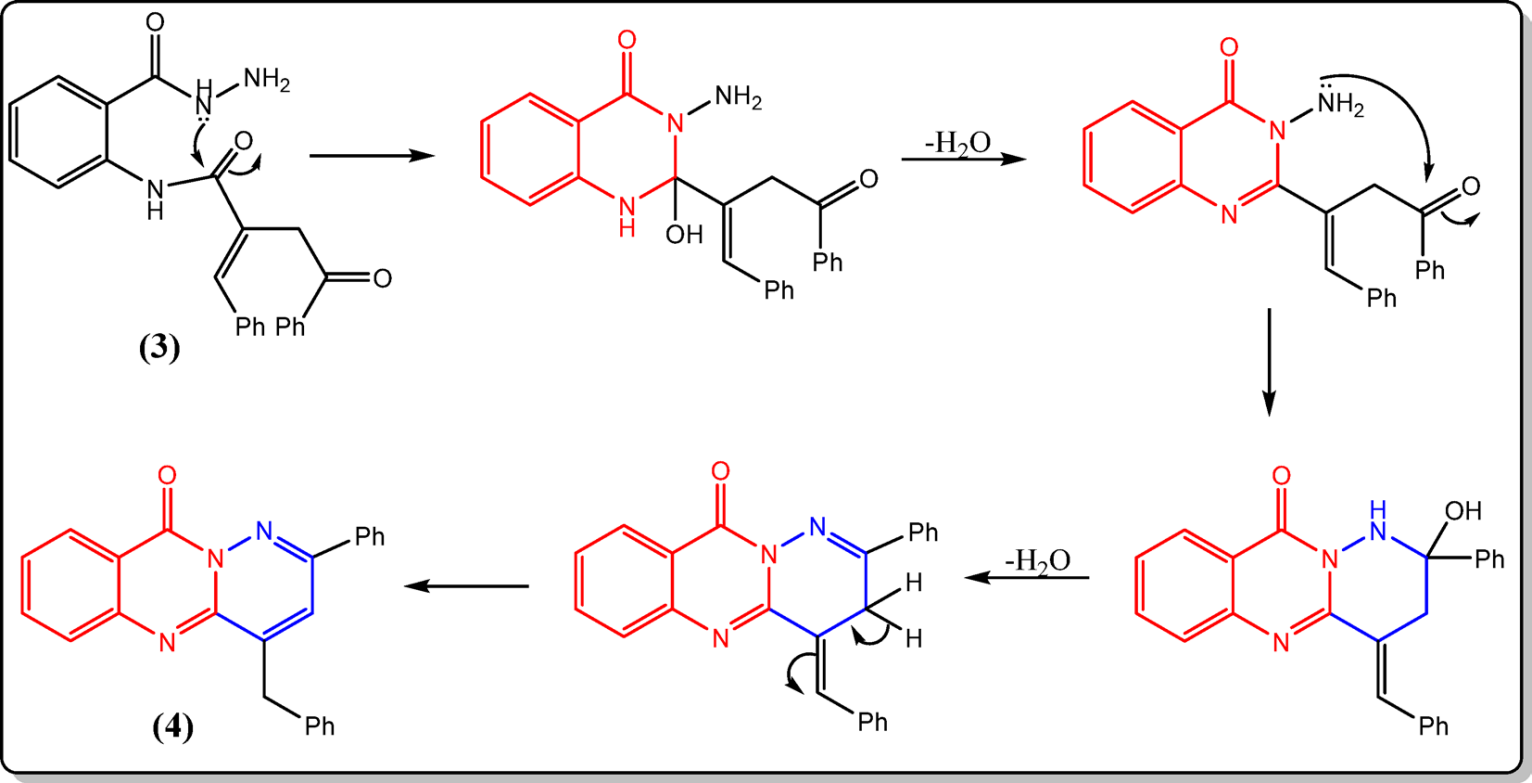
Reactions of **3** with acid and/or acid anhydride.

**Fig. 5:**
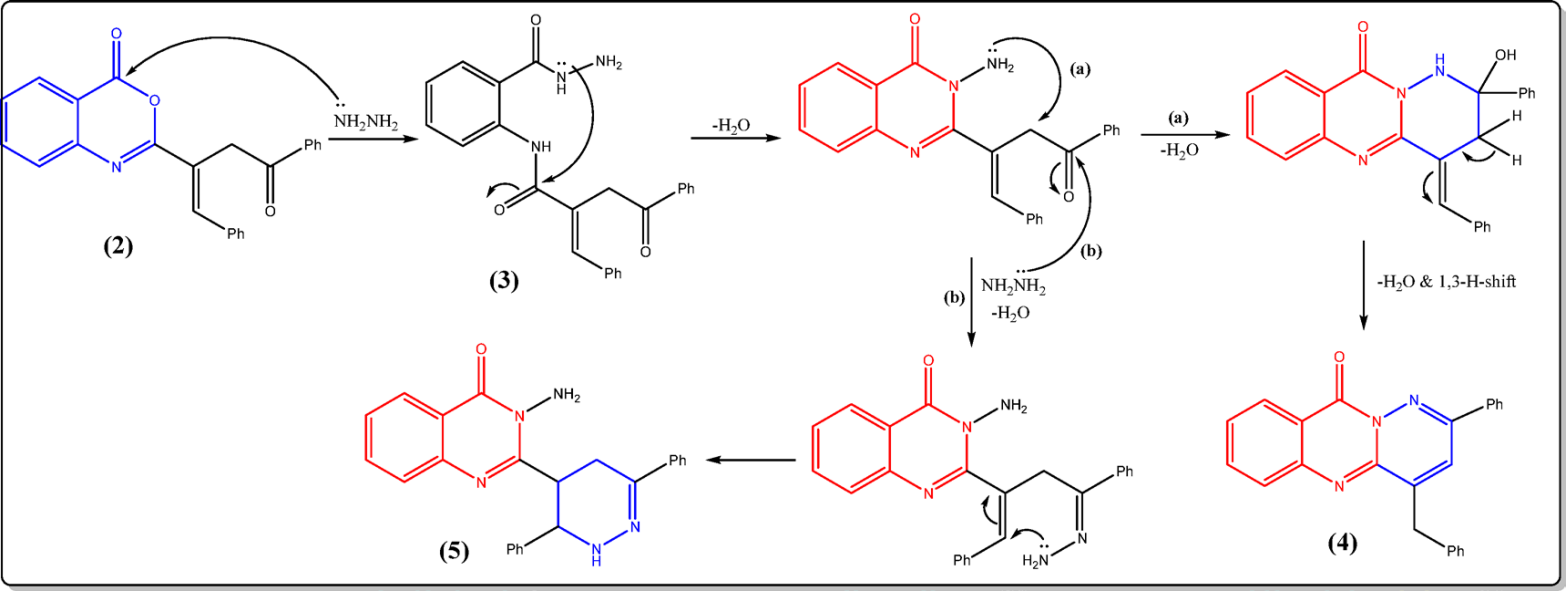
Reactions of **3** with carbon electrophiles.

**Fig. 6:**
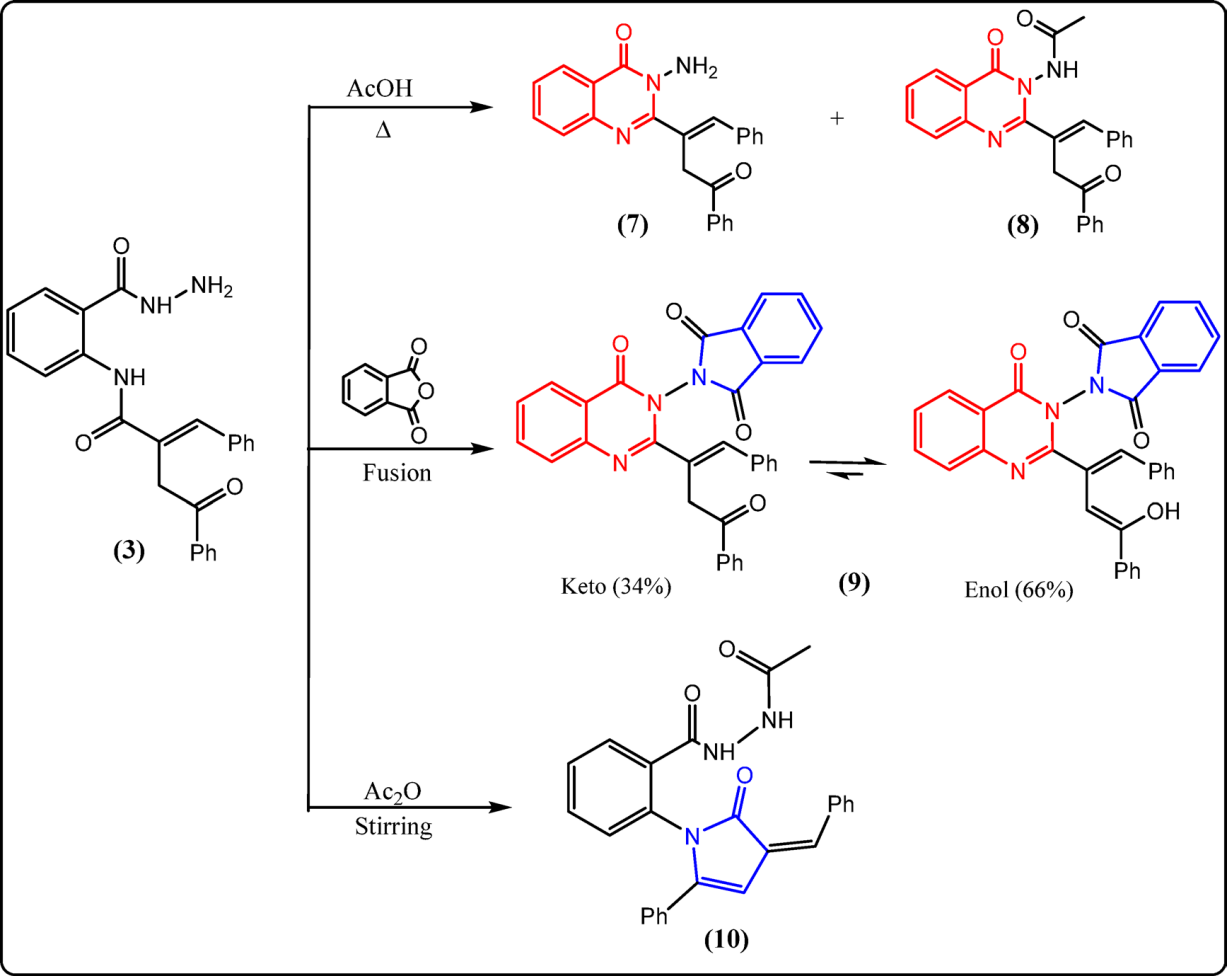
Reactions of **3** with carbonyl compounds.

The synthetic routes used to get the desired compounds **2–15** in reasonably high yields are outlined in Figs. 2, 3, 4, 5 and 6. Nucleophilic addition of anthranilic acid to furanone **1** produced the starting compound 2-substituted benzoxazin-4-one **2** (Fig. 2).

Compound **2** was elucidated via its spectroscopic data and micro-analytical. Its IR spectrum revealed a prominent band at 1741 cm^−1^ that corresponds to the C=O of the oxazinone, alongside a band at 1710 cm^−1^ related to ketonic group. Moreover, the ^1^H-NMR spectrum of **2** indicated the existence of two doublet signals at δ 3.47 & 3.95 due to geminal coupling between two protons of methylene group (CH_2_), with coupling constant *J* = 18.3 Hz. Further support for chemical structure of 2 was obtained from its ^13^C-NMR and accurate elemental analysis data (cf. Experimental). The authors aimed to use the key starting compound **2** to prepare new quinazolinone and hydrazide derivatives by reacting oxazinone **2** with two nitrogen nucleophiles: hydrazine hydrate and ammonium acetate (Fig. 3). Stirring of **2** at room temperature with an adequate amount of hydrazine hydrate in dioxane afforded the acid hydrazide derivative **3** in a good yield, which was then cyclized under reflux in n-butanol or by fusion to give quinazolinone derivative **4** in 60% yield (Fig. 3). Suggesting structures 3 & 4 seemed to be acceptable according to their spectroscopic and elemental analyses. Compound 3’s infrared spectrum exhibited bands that were characteristic of the NH, NH_2_, and C=O groups. The ^1^H-NMR spectrum of **3** indicated additional exchangeable broad singlet signals, which were consistent with the presence of lactam–lactim tautomers, with a ratio of 54:46, corresponding to the integration values for the NH and OH groups, respectively.

**Fig. 7:**
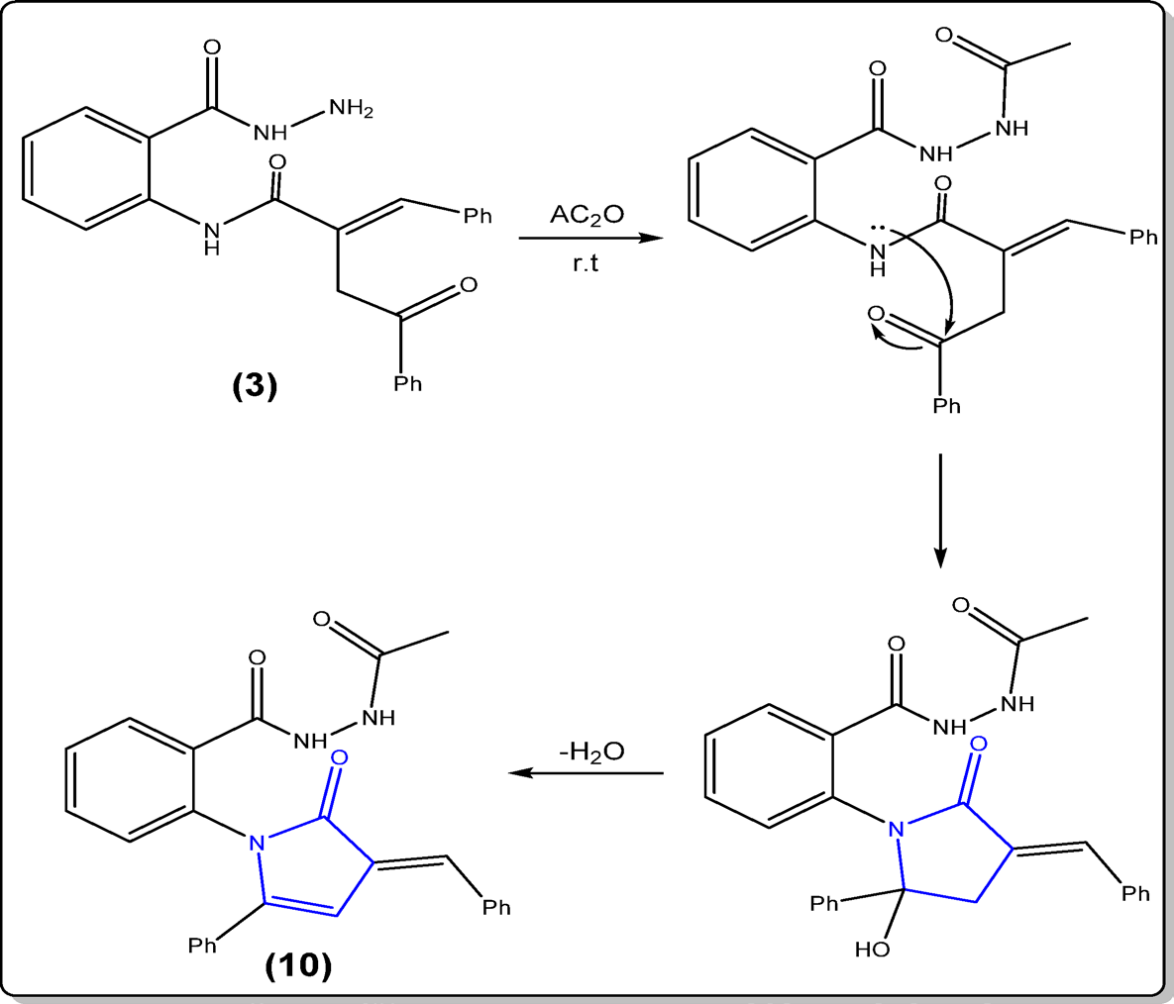
Mechanistic pathway for cyclization of **3** to **4**.

Also, the ^13^C-NMR spectrum supported the proposed structure of compound **3**, in which three peaks appeared at δ 157.36, 158.55, and 188.55 ppm, corresponding to the three C=O groups (cf. Experimental). The chemical structure of compound **4** was elucidated by analyzing its infrared spectrum, which did not show any absorptions corresponding to the NH and NH_2_ groups. Instead, it exhibited a band for the quinazolinone carbonyl at 1689 cm^−1^. Additional evidence was obtained from its ^1^H- and ^13^C-NMR spectra, which were consistent with the suggested structure (cf. Experimental). The following mechanism (Fig. 7) summarizes the cyclization of compound **3** to **4**.

**Fig. 8:**
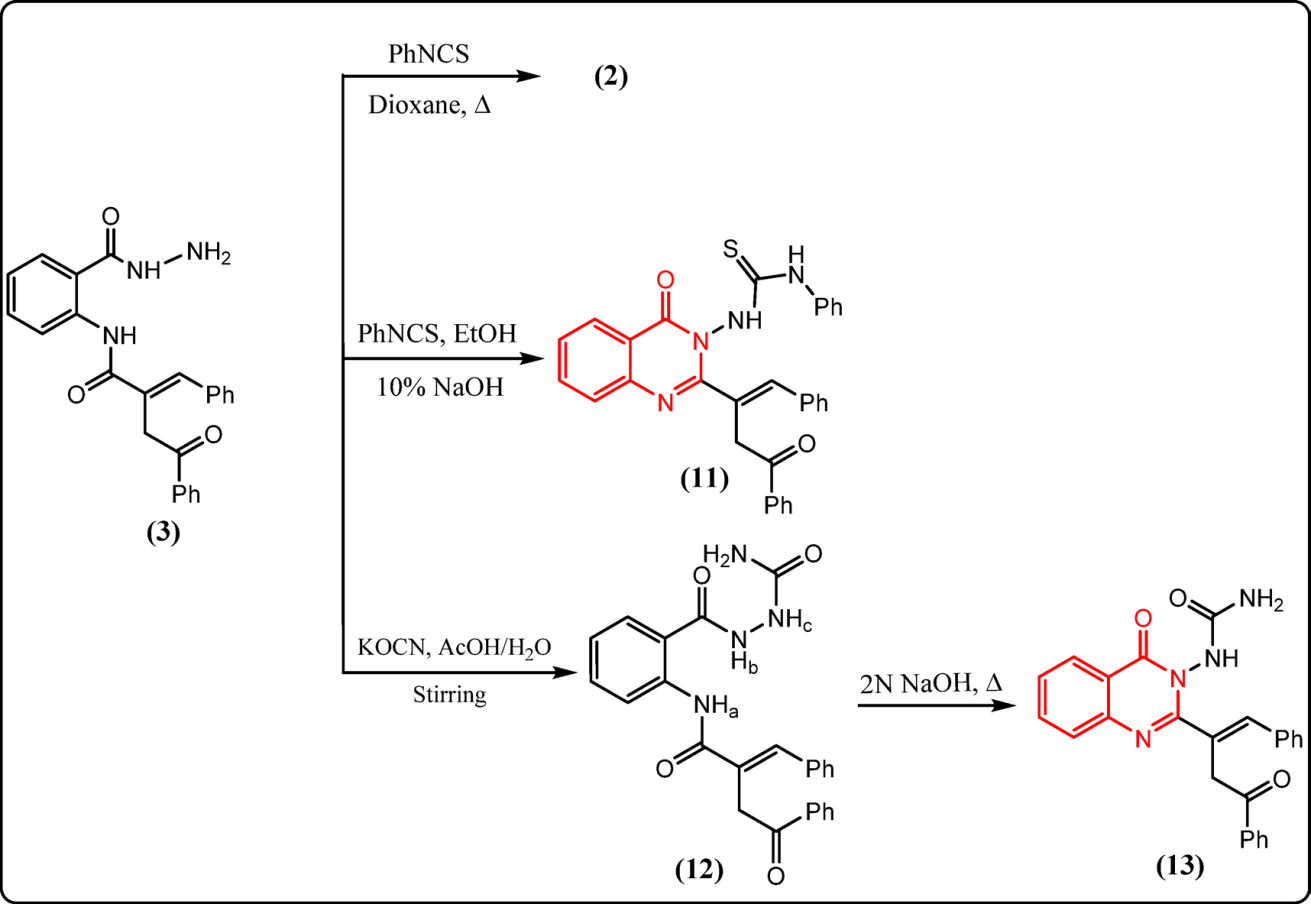
Mechanistic pathway for conversion of **2** into **4** and **5**.

On the other hand, refluxing oxazinone derivative **2** with an excess amount of hydrazine hydrate in dioxane yielded a mixture of compound **4** and the amino quinazolinone derivative **5** in 40% yield (Fig. 3). The suggested structure for compound **5** was confirmed through spectral and analytical data. The presence of bands belonging to the NH, NH_2_, and C=O groups was shown in the infrared spectrum (cf. Experimental). The ^1^H-NMR spectrum of compound **5** showed exchangeable singlet signals corresponding to NH_2_ and NH at 5.83 and 8.24 ppm, respectively, along with a doublet of triplets for the PhCH*CH* proton, a doublet for the PhCHCH proton, and two doublets of doublets related to CH_2_ protons at 4.26, 5.25, and 2.69 & 2.87 ppm, respectively. The ^13^C-NMR spectrum and accurate elemental analysis also confirmed the suggested structure of compound **5** (cf. Experimental). The mechanistic pathway of the reaction is depicted in Fig. 8.

When the benzoxazinone derivative **2** was fused with ammonium acetate in a thermally controlled oil bath at 160 °C, a brown solid product was separated and identified as 2-(2,5-diphenylfuran-3-yl)quinazolin-4(3*H*)-one (**6**) (Fig. 3). Its structure was supported by spectral and elemental data values, which were in agreement with those expected for the postulated structure. The infrared spectrum of quinazolinone derivative **6** displayed the disappearance of the absorption band of the oxazinone carbonyl and the appearance of absorption bands corresponding to NH and C=O of cyclic amides at 3151 and 1692 cm^−1^, respectively. Moreover, its ^1^H-NMR spectrum exhibited an exchangeable broad singlet signal for the NH proton (cf. Experimental). The ascribed structure of **6** was further supported by its ^13^C-NMR spectrum, which revealed only one signal for the carbonyl carbon at 170.99 ppm, along with other important signals for the remaining carbons (cf. Experimental).

A series of chemical reactions with benzoyl hydrazine derivative **3** were performed as chemical evidence supporting the proposed structure and also to prepare some new derivatives with expected antitumor activity. This class of compounds has been reported to exhibit a wide variety of biological applications^53–62^. These reactions are discussed as shown in Figs. 4 and 5, and 6. When the hydrazide derivative **3** was refluxed with boiling acetic acid, the corresponding amino quinazolinone derivative **7** was formed, which was easily acetylated to the corresponding acetylated derivative **8** (Fig. 4). The structures of both **7** and **8** were determined using their spectroscopic and elemental data. Their IR spectra exhibited new absorption bands at 1639 and 1643 cm^−1^, respectively, corresponding to the C=N group, which was not present in hydrazide derivative **3**. The ^1^H-NMR spectrum of acetylated derivative **8** revealed a new singlet signal at 2.13 ppm corresponding to the methyl group protons. ^13^C-NMR for both compounds **7** and **8** exhibited signals at δ 142.54 and 142.21 ppm, respectively, corresponding to the C=N group (cf. Experimental).

The hydrazide derivative **3** was fused with phthalic anhydride to give quinazolinone derivative **9** (Fig. 4). The spectral and elemental data of compound **9** confirmed its structure. Its IR spectra revealed bands associated with the carbonyl phthalimido moiety at 1795 (asymmetric) and 1738 cm^−1^ (symmetric). A singlet signal at 3.56 ppm for methylene protons (34%), a singlet signal at 11.32 ppm for the hydroxy proton (66%), as well as an extra singlet signal for the methine (CH=) proton, were detected in the ^1^H-NMR spectrum. This provides convincing proof that compound **9** exists in DMSO as an equilibrium mixture of keto–enol tautomers in a ratio of 34:66, based on the signal integration percentages (Fig. 4).

**Fig. 9:**
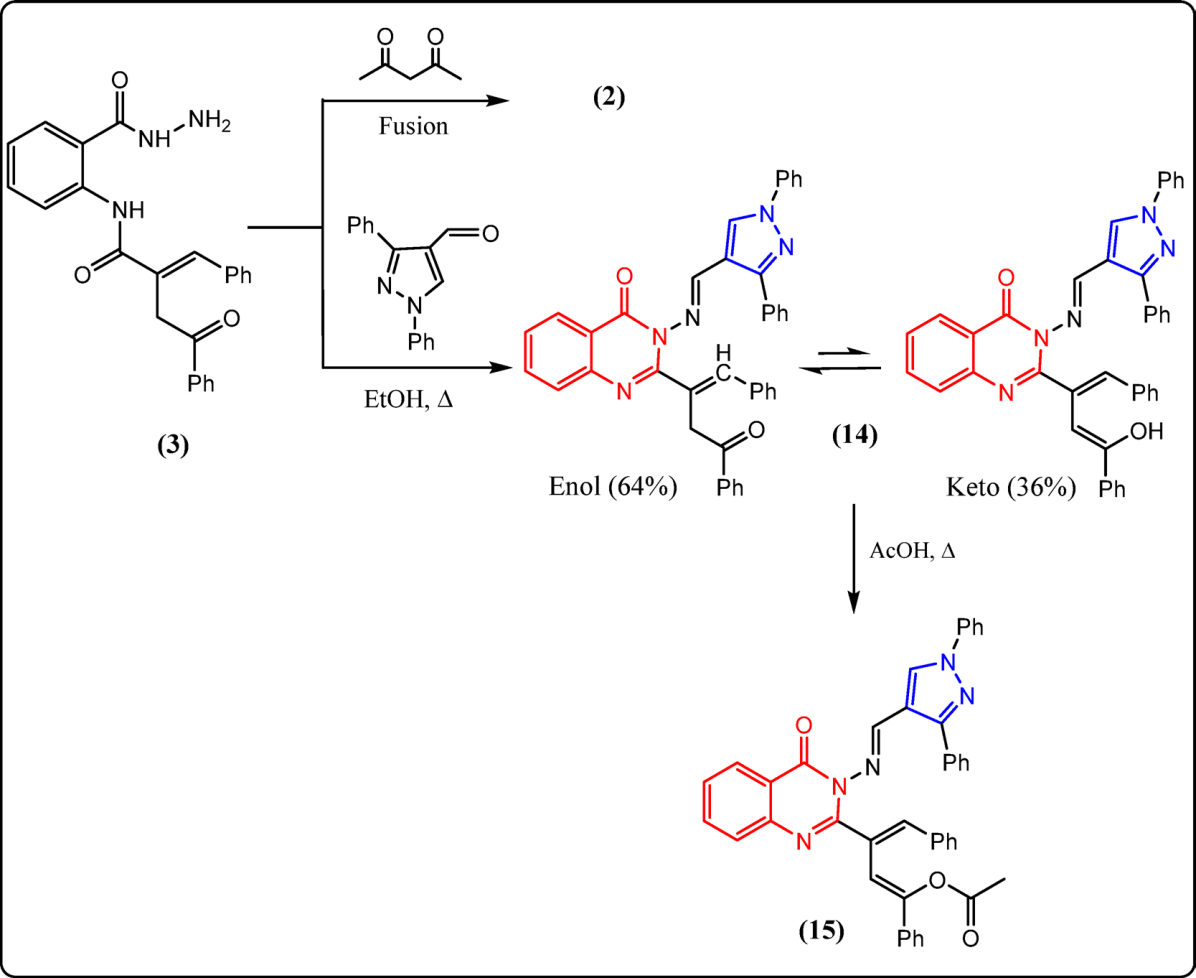
Mechanistic pathway for formation of **10**.

Stirring of hydrazide derivative **3** with freshly distilled acetic anhydride at room temperature gave pyrrolone derivative **10** (Fig. 4), according to the following mechanism (Fig. 9).

As shown in Fig. 9, acetic anhydride acts as both an acetylating and a dehydrating agent, leading to cyclization and formation of the pyrrolone ring. The spectral and elemental data confirmed the suggested structure for compound **10** (cf. Experimental).

On the other hand, the authors investigated the chemical behavior of hydrazide derivative **3** toward electrophiles such as phenyl isothiocyanate and potassium cyanate. However, when derivative **3** was refluxed with phenyl isothiocyanate in dioxane, internal hydrolysis occurred due to nucleophilic attack by the lactim OH on the hydrazide carbonyl instead of attack by NH_2_ on PhNCS, forming back the benzoxazinone derivative **2** (Fig. 5), as confirmed by TLC, m.p., and m.m.p. with compound **2**. Because of the low activity of the hydrazide toward phenyl isothiocyanate, the reaction was catalyzed under basic conditions by adding ethanolic sodium hydroxide to the refluxed mixture of **3** and phenyl isothiocyanate, which afforded the quinazolinone derivative **11** (Fig. 5). The basic medium activates the amidic hydrogen, promoting attack on the amide carbonyl and forming a six-membered amino-quinazolinone. This amino intermediate then attacked PhNCS, yielding compound **11**. The infrared spectrum of **11** displayed bands associated with NH, C=S, and C=O groups (cf. Experimental). Its ^1^H-NMR spectrum exhibited exchangeable signals at δ 11.24 and 13.60 ppm for two NH protons. Moreover, its ^13^C-NMR spectrum showed signals at 152.79, 154.81, and 170.58 ppm for C=S and the two C=O carbons, respectively.

When an acetic acid solution of **3** was stirred with potassium cyanate at room temperature, the urea derivative **12** formed in good yield (Fig. 5), then cyclized to the quinazolinone derivative **13** (Fig. 5) upon reflux with ethanolic sodium hydroxide.

The structures of compounds **12** and **13** have been inferred from their analytical and spectral data (cf. Experimental). The hydrazide derivative **3** was allowed to react with two carbonyl compounds, such as acetylacetone and/or diphenylpyrazole-4-carbaldehyde, to examine the nucleophilicity of the amino group. Due to the low reactivity of acetylacetone, fusion of it with hydrazide derivative **3** gave the benzoxazinone derivative **2** (Fig. 6), which was verified by comparison with compound **2**.

According to the chemotherapeutic potential of pyrazole derivatives, the authors aimed to construct a new pyrazole ring by treating compound **3** with 1,3-diphenyl-1*H*-pyrazole-4-carbaldehyde in an ethanolic solution under reflux, which afforded the pyrazoloquinazoline derivative **14** (Fig. 6).

It is noteworthy that its ^1^H-NMR spectrum displayed a singlet signal at 3.50 ppm related to CH_2_ (64%) and a broad singlet signal at 11.94 ppm (exchangeable) related to OH (36%), which confirmed the presence of compound **14** in DMSO solution as a keto–enol tautomers mixture in a 64:36 ratio.

As chemical proof, the hydroxyl group in compound **14** was acetylated when it was refluxed with an acetic acid solution to give quinazoline derivative **15** in excellent yield (Fig. 6). Utilizing spectral and elemental data, the structure of compound **15** was established (cf. Experimental).

### Cytotoxic activity against human tumor cells using the MTT assay

The newly synthesized compounds **2–15** were evaluated for in vitro cytotoxicity against two human cancer cell lines: HEPG2 (liver carcinoma) and MCF7 (breast adenocarcinoma), using doxorubicin as a reference drug. IC_50_ values were determined using nonlinear regression (four-parameter logistic model), and are presented as best-fit estimates ± standard error (SE) in Table 1. The data reflects pooled results from three independent biological experiments per compound.

Based on the IC_50_ classification scale (*1–10 µM: very high; 11–20 µM: high; 21–50 µM: moderate; 51–100 µM: low; >100 µM: non-cytotoxic*), compound **3** demonstrated very high cytotoxic activity against MCF7 cells (IC_50_ = 3.84 ± 0.2 µM), even slightly lower than doxorubicin (4.17 ± 0.2 µM). Compound **5** showed high activity against HEPG2 cells (IC_50_ = 6.90 ± 0.4 µM), approaching the potency of doxorubicin (4.50 ± 0.2 µM).

Compounds **8** and **10** exhibited high to moderate cytotoxic activity in both cell lines, with IC_50_ values between approximately 10 and 18 µM. Compounds **12** and **13** also showed consistent cytotoxic potential, with moderate to high activity depending on the cell line. Compound **14** exhibited moderate activity, while compounds **2**, **4**, and **11** were less potent, with IC_50_ values in the low activity range (> 50 µM). Compounds **7**, **9**, and **15** presented cell line–dependent responses, ranging from moderate to low potency.

Structure–activity analysis suggests that compounds **3** and **5**, which contain hydrazide, lactam, lactim, and phenyl functional groups, may benefit from functional elements known to enhance cytotoxicity. In addition, the presence of acetamido, acetyl, amidic, and quinazolinone moieties in compounds **8**, **10**, **12**, and **13** may contribute to their observed activity. These groups are commonly present in clinically relevant anticancer agents such as doxorubicin, sorafenib, and gefitinib.

It should be noted that no formal statistical comparisons were performed between IC_50_ values. Therefore, potency conclusions are descriptive, based on numerical trends and standard errors derived from nonlinear regression models.

**Table 1:** In vitro cytotoxicity (IC_50_, µM ± SE) of compounds 2–15 compared to doxorubicin, evaluated in HEPG2 and MCF7 human cancer cell lines. IC_50_ values were determined by 4-parameter nonlinear regression from pooled triplicate data across three independent experiments.

Compound	HEPG2 IC_50_ ± SE (µM)	MCF7 IC_50_ ± SE (µM)
**Doxorubicin**	4.50 ± 0.2	4.17 ± 0.2
**2**	56.14 ± 3.1	61.24 ± 3.5
**3**	8.96 ± 0.6	3.84 ± 0.2
**4**	68.32 ± 3.8	54.22 ± 3.2
**5**	6.90 ± 0.4	5.58 ± 0.3
**6**	38.12 ± 2.4	9.14 ± 0.7
**7**	63.57 ± 3.6	39.27 ± 2.4
**8**	10.48 ± 0.9	18.12 ± 1.4
**9**	52.18 ± 2.9	29.28 ± 2.1
**10**	13.09 ± 1.1	15.47 ± 1.2
**11**	83.60 ± 4.1	78.69 ± 4.0
**12**	27.73 ± 2.0	7.76 ± 0.5
**13**	19.38 ± 1.4	23.68 ± 1.8
**14**	32.56 ± 2.2	34.66 ± 2.3
**15**	75.51 ± 3.9	44.40 ± 2.6

IC_50_ classification: 1–10 µM (very high), 11–20 µM (high), 21–50 µM (moderate), 51–100 µM (low), > 100 µM (non-cytotoxic).

## Molecular docking assessment

To gain insight into the molecular interactions of the synthesized quinazolinone derivatives, molecular docking studies were conducted against five key cancer-related targets: Topoisomerase II (PDB: 5ZAD), VEGFR2 (PDB: 3WZE), c-Met (PDB: 3U6I), EGFR (PDB: 1M17), and ERα (PDB: 3ERT). These targets were selected for their critical roles in DNA replication, angiogenesis, metastasis, and tumor proliferation^33,34,36,38,41,44,48–50^.

**Fig. 10:**
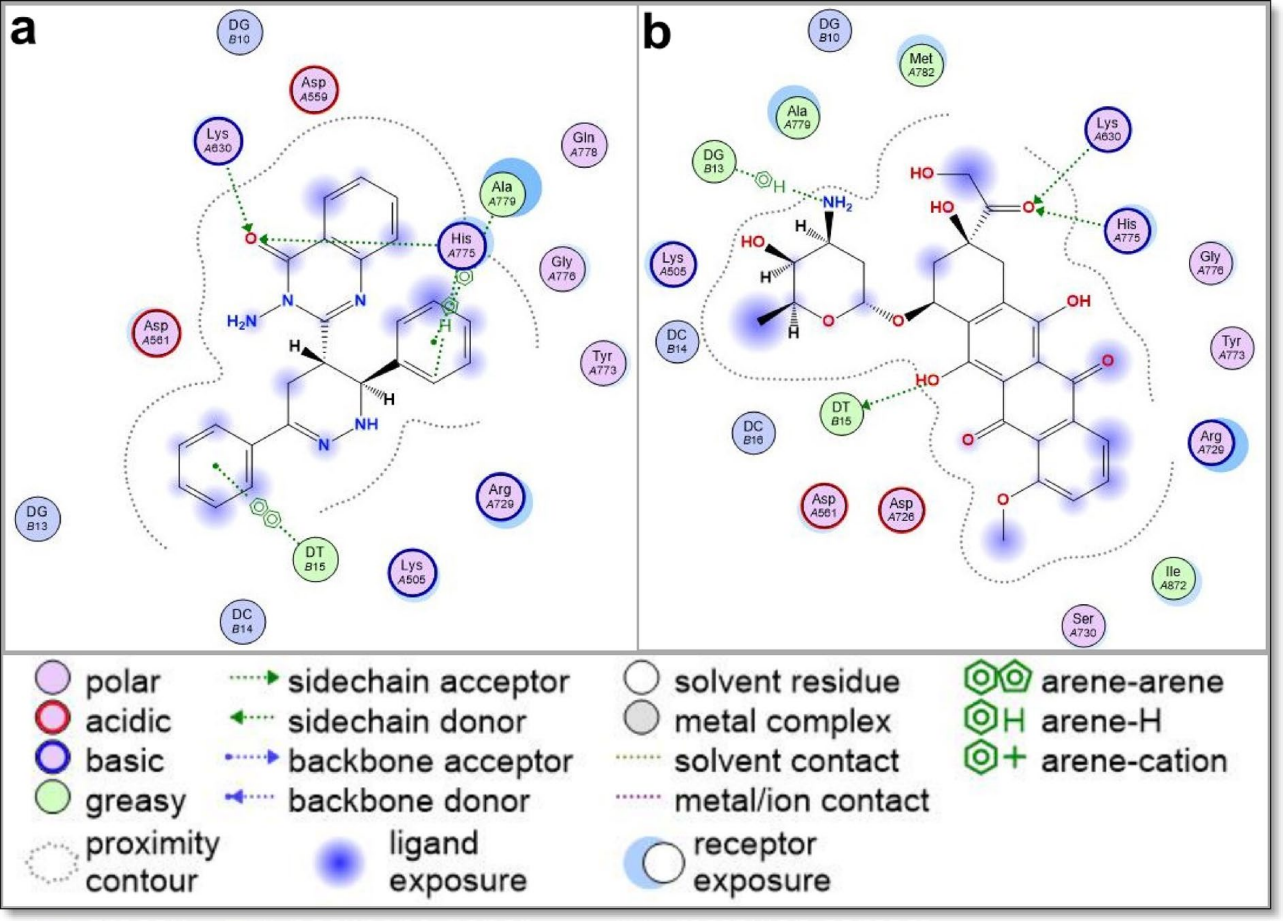
2D interaction diagram of Quinazolinone Derivative **5** with (**a**) topoisomerase II (PDB: 5ZAD) compared to (**b**) doxorubicin, a known topoisomerase II inhibitor. The diagram illustrates key binding interactions, including hydrogen bonds, hydrophobic contacts, and π-π stacking, highlighting the binding mode similarities and differences between Quinazolinone Derivative **5** and the reference drug doxorubicin. These interactions suggest the potential of Quinazolinone Derivative **5** as a topoisomerase II inhibitor.

**Fig. 11:**
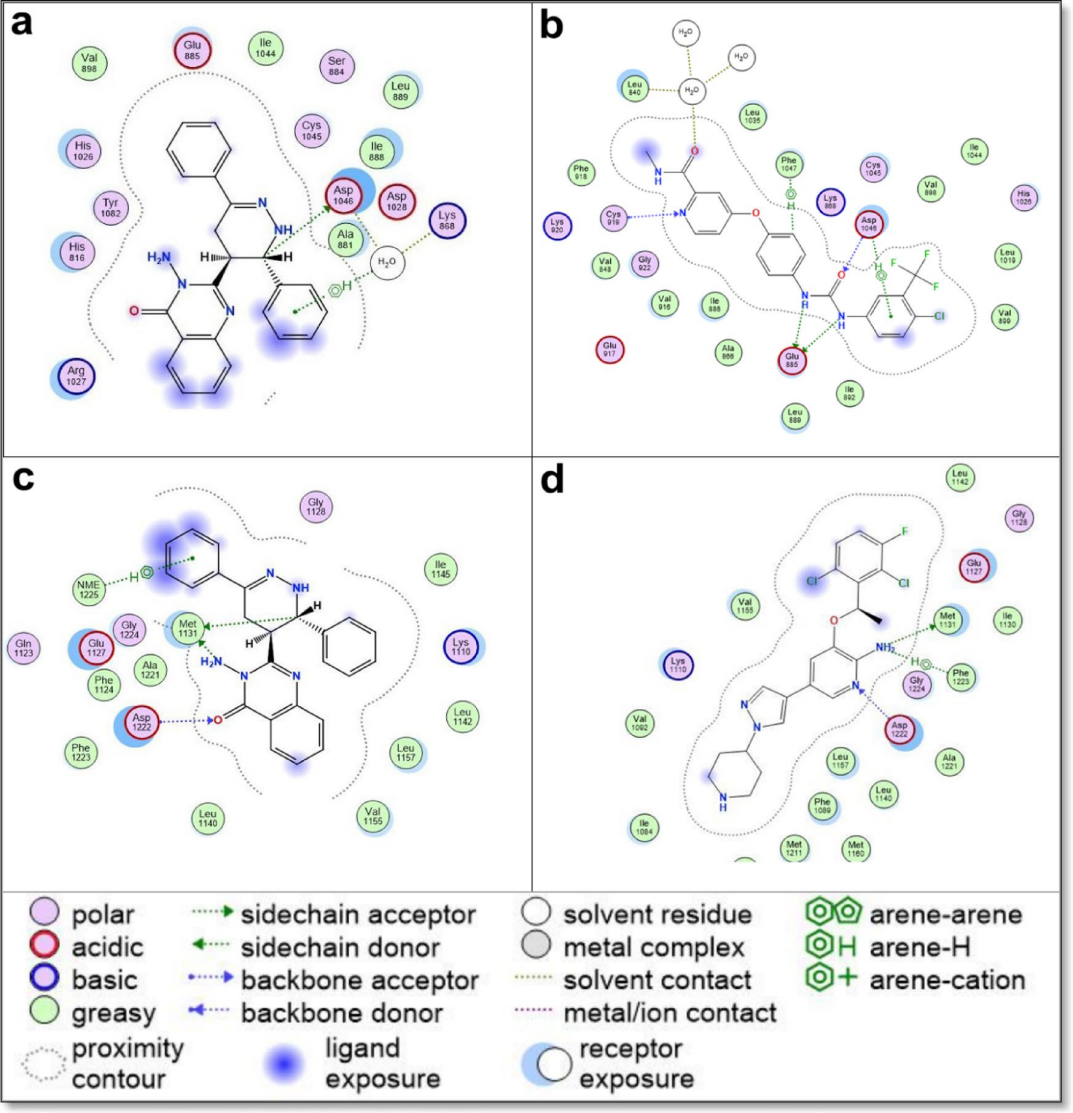
2D interaction diagrams of Quinazolinone Derivative **5** with key molecular targets, compared to reference inhibitors. (**a**) Interaction of Quinazolinone Derivative **5** with VEGFR2 (PDB: 3WZE), showing binding interactions relative to (**b**) sorafenib, a known VEGFR2 inhibitor. (**c**) Interaction of Quinazolinone Derivative 5 with c-MET (PDB: 3U6I), compared to (**d**) crizotinib, a well-established c-MET inhibitor. The diagrams highlight key molecular interactions, including hydrogen bonds, hydrophobic interactions, and π-π stacking, providing insights into the binding mode and potential inhibitory activity of Quinazolinone Derivative **5** against VEGFR2 and c-MET.

**Fig. 12:**
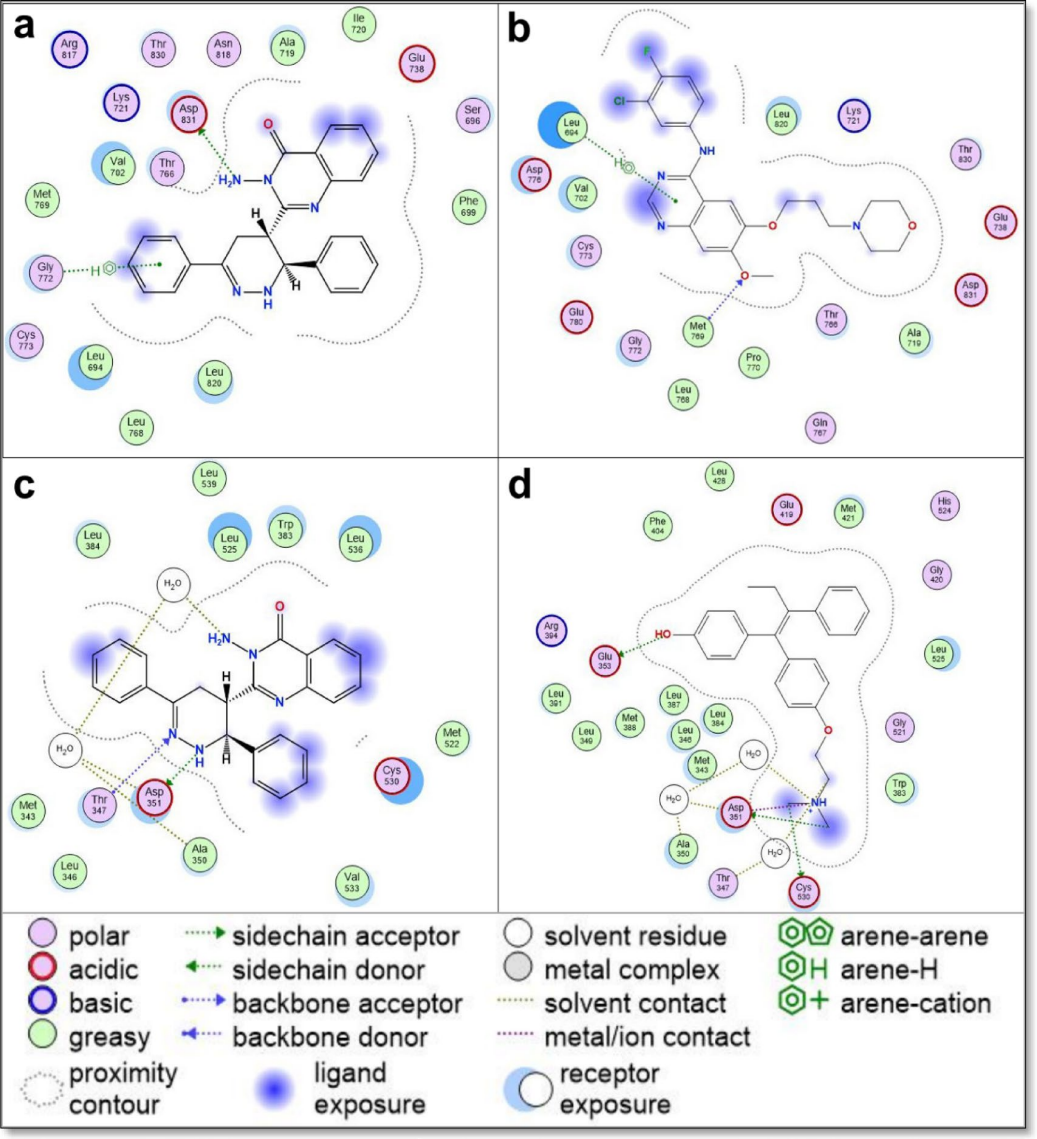
2D interaction diagrams of Quinazolinone Derivative **5** with key molecular targets, compared to reference inhibitors. (**a**) Interaction of Quinazolinone Derivative **5** with EGFR (PDB: 1M17), demonstrating a distinct binding mode compared to (**b**) gefitinib, a known EGFR inhibitor. (**c**) Interaction of Quinazolinone Derivative **5** with Estrogen Receptor Alpha (ERα) (PDB: 3ERT), compared to (**d**) 4-hydroxytamoxifen, which serves as both the reference ligand and the co-crystallized ligand in the 3ERT structure. The diagrams highlight key molecular interactions, including hydrogen bonding, hydrophobic contacts, and π-π stacking, providing insights into the differential binding modes of Quinazolinone Derivative **5** across these targets.

The docking results, summarized in Table 2, present the binding energy scores for all tested derivatives and reference inhibitors. Detailed interaction profiles—including hydrogen bonding, π–π stacking, and hydrophobic interactions—are provided in Supplementary Data File 2. To better understand the mechanism of action for the most cytotoxic compound, compound **5**, its binding interactions with all five molecular targets are further illustrated in Table 3; Figs. 10, 11 and 12, alongside comparisons to known reference inhibitors.

Most of the newly synthesized quinazolinone derivatives exhibited binding modes similar to their respective reference drugs, engaging essential active site residues through a combination of hydrogen bonds, π–π stacking, and hydrophobic contacts. Key interacting residues across the targets included LYS630 and HIS775 in Topoisomerase II, ASP1046 and GLU885 in VEGFR2, ASP1222 and MET1131 in c-Met, ASP831 and GLY772 in EGFR, and ASP351 and GLU353 in ERα—suggesting good structural compatibility between the compounds and target binding pockets. Supplementary Data File 2 provides a comprehensive account of all ligand–receptor interactions, including interaction types, distances, and binding energy values.

To explain the potent cytotoxic activity of compound **5**, its docking interactions were examined in greater detail. In Topoisomerase II, compound **5** formed strong hydrogen bonds with LYS630 and HIS775, as well as π–π stacking with DT15, suggesting DNA intercalation potential similar to doxorubicin (Fig. 10). This interaction supports the compound’s ability to inhibit DNA replication and repair, leading to apoptosis in cancer cells.

In VEGFR2, compound **5** engaged in hydrogen bonding with ASP1046 and formed water-mediated interactions, closely mimicking the binding pattern of sorafenib (Fig. 11a and b). These interactions suggest anti-angiogenic potential via VEGFR2 inhibition. In c-Met, which is critical for tumor invasion and metastasis, compound **5** formed hydrogen bonds with MET1131 and ASP1222, similar to crizotinib (Fig. 11c and d), but also displayed additional hydrophobic interactions, which may enhance binding affinity and stability.

In the EGFR binding site, compound 5 formed hydrogen bonds with ASP831 and GLY772, differentiating its binding profile from gefitinib, which typically binds MET769 and LEU694 (Fig. 12a and b). These distinct interactions may help explain compound **5**’s strong in vitro cytotoxicity despite only moderate docking scores. For ERα, compound **5** showed hydrogen bonding with ASP351 and additional water-mediated contacts, closely resembling the interaction mode of 4-hydroxytamoxifen (Fig. 12c and d).

A full overview of docking scores is provided in Table 2, revealing variable binding affinities across different compounds and targets. While several derivatives achieved docking scores higher than those of their respective reference inhibitors, this did not necessarily imply novel binding modes. For example, compound **11** exhibited a higher docking score (− 8.21 kcal/mol) than doxorubicin (− 8.05 kcal/mol) in Topoisomerase II, while maintaining identical hydrogen bonding interactions with LYS630 and HIS775, demonstrating that increased affinity does not always reflect new binding mechanisms.

Similarly, compound **9** showed strong binding affinity to VEGFR2 (− 8.38 kcal/mol) through interactions with ASP1046 and HIS1026, similar to sorafenib, despite having slightly lower numerical scores than other derivatives. These observations emphasize the importance of evaluating both quantitative binding affinities and qualitative interaction profiles when assessing potential anticancer candidates.

Overall, the molecular docking results suggest that the majority of quinazolinone derivatives interact favorably with critical cancer targets, often in ways comparable to established inhibitors. Among them, compound **5** stood out for its potent cytotoxicity and versatile interaction patterns across all five targets. Its combination of hydrogen bonding, π–π stacking, and hydrophobic contacts likely contributes to its high biological activity. Derivatives such as compounds **14** and **15** also exhibited alternative but meaningful binding patterns, underscoring their potential as lead structures for future optimization.

Complete interaction data for all tested compounds is available in Supplementary Data File 2, serving as a valuable resource for further structural analysis and rational drug design. Moving forward, experimental validation through enzyme inhibition assays, in vitro mechanistic studies, and molecular dynamics-based refinement will be essential to confirm and optimize the anticancer potential of these derivatives for potential clinical development.

**Table 2:** Binding energy scores (S) and root mean square deviation (RMSD) values of Quinazolinone derivatives docked against five Cancer-Related targets: topoisomerase II (PDB ID: 5ZAD), VEGFR2 (3WZE), c-Met (3U6I), EGFR (1M17), and Estrogen receptor alpha (3ERT).

Compound	Topoisomerase II (PDB: 5ZAD)		VEGFR2 (PDB: 3WZE)		c-Met (PDB: 3U6I)		EGFR (PDB: 1M17)		ERα (PDB: 3ERT)	
	S	RMSD	S	RMSD	S	RMSD	S	RMSD	S	RMSD
2	-6.69	1.47	-7.70	1.49	-7.47	1.56	-7.99	1.22	-7.56	1.01
3_Lactam	-7.04	1.84	-7.72	1.59	-8.85	1.27	-7.75	1.19	-7.92	1.76
3_Lactim	-6.75	1.61	-7.98	1.81	-8.51	1.55	-7.86	1.31	-8.08	1.92
4	-6.47	1.63	-6.50	1.63	-7.30	1.37	-6.79	1.18	-5.88	1.50
5	-6.74	1.76	-7.41	1.80	-8.10	1.38	-7.25	1.17	-5.70	1.56
6	-6.70	1.12	-7.06	1.47	-7.13	1.05	-6.66	1.20	-5.84	1.43
7	-6.84	1.11	-8.11	1.41	-7.73	1.33	-7.38	1.19	-6.12	1.50
8	-7.43	1.60	-7.56	1.04	-8.45	1.21	-7.32	1.61	-6.30	1.43
9_Enol	-7.67	1.96	-8.38	1.40	-9.08	1.95	-7.27	1.74	-5.79	1.72
9_Keto	-7.75	1.56	-7.51	1.81	-8.70	1.88	-7.43	0.91	-7.63	1.82
10	-7.44	1.12	-7.76	1.47	-8.46	1.09	-7.49	1.90	-6.76	1.13
11	-8.21	1.78	-7.94	1.85	-8.76	1.90	-8.57	1.98	-5.86	1.50
12	-7.11	1.38	-8.12	1.93	-8.35	1.53	-8.01	1.68	-8.17	1.61
13	-7.14	1.64	-7.53	1.64	-8.64	1.18	-8.69	1.63	-7.80	1.67
14_Enol	-8.16	1.47	-8.17	1.73	-9.09	1.84	-7.45	1.98	-7.40	1.85
14_Keto	-8.64	1.48	-6.44	1.61	-7.60	1.87	-8.17	1.88	-5.43	1.49
15	-8.51	1.99	-8.08	1.98	-8.87	1.90	-8.59	1.59	-7.07	1.77
Reference	Doxorubicin		Sorafenib		Crizotinib		Gefitinib		4HYD.TF	
	-8.05	1.83	-9.66	0.77	-8.03	1.32	-8.12	1.29	-8.67	1.53

Reference inhibitors included doxorubicin, sorafenib, crizotinib, gefitinib, and 4-hydroxytamoxifen.

**Table 3:** Summary of key hydrogen bonding, π–π stacking, and hydrophobic interactions of Quinazolinone derivative 5 with active site residues of five target proteins.

Complex	Residues	Interaction type	Distance (Å)	E (kcal/mol)
5- Topoisomerase II (PDB: 5ZAD)	LYS 630 (A)	H-acceptor	2.86	-15.8
	HIS 775 (A)	H-acceptor	2.98	-4.9
	HIS 775 (A)	H-pi	3.61	-0.5
	ALA 779 (A)	pi-H	4.24	-0.5
	DT 15 (F)	pi-pi	4.15	
Doxorubicin- Topoisomerase II (PDB: 5ZAD)	DT 15 (F)	H-donor	2.94	-1.2
	LYS 630 (A)	H-acceptor	3.48	-2
	HIS 775 (A)	H-acceptor	2.93	-5.2
	DG 13 (F)	H-pi	4.85	-0.5
5-VEGFR2 (PDB: 3WZE	ASP 1046 (A)	H-donor	3.48	-0.7
	HOH 3064 (A)	pi-H	4.7	-0.5
Sorafenib-VEGFR2 (PDB: 3WZE)	GLU 885 (A)	H-donor	2.84	-5
	GLU 885 (A)	H-donor	3.02	-4.8
	ASP 1046 (A)	H-acceptor	3	-1.2
	HOH 3089 (A)	H-acceptor	2.82	-1.7
	CYS 919 (A)	H-acceptor	3.28	-1.8
	PHE 1047 (A)	H-pi	3.8	-0.8
	ASP 1046 (A)	pi-H	4.22	-0.5
5-c-Met (PDB: 3U6I)	ET 1131 (A)	H-donor	3.74	-0.8
	MET 1131 (A)	H-donor	3.61	-2.3
	ASP 1222 (A)	H-acceptor	2.95	-1.3
	NME 1225 (A)	pi-H	3.68	-0.6
Crizotinib-c-Met (PDB: 3U6I)	MET 1131 (A)	H-donor	3.12	-1
	ASP 1222 (A)	H-acceptor	2.88	-2.6
	PHE 1223 (A)	H-pi	4.27	-0.5
5-EGFR (PDB: 1M17)	ASP 831 (A)	H-donor	3.03	-3.8
	GLY 772 (A)	pi-H	3.39	-0.6
Gefitinib- EGFR (PDB: 1M17)	MET 769 (A)	H-acceptor	3.18	-0.8
	LEU 694 (A)	pi-H	3.93	-1.3
5-ERα (PDB: 3ERT)	ASP 351 (A)	H-donor	3.34	-1.5
	HOH 58 (A)	H-donor	3.03	-1.9
	THR 347 (A)	H-acceptor	3.6	-0.5
4-Hydroxytamoxifen- ERα (PDB: 3ERT)	GLU 353 (A)	H-donor	2.92	-3.1
	HOH 31 (A)	H-donor	3.18	-0.7
	HOH 58 (A)	H-donor	3.2	-1.8
	CYS 530 (A)	H-donor	3.61	-2.1
	ASP 351 (A)	H-donor	3.54	-0.6
	ASP 351 (A)	Ionic	3.28	-2.8

Interaction types and distances (in Å) are reported along with the estimated binding energy values (E, kcal/mol).

## Molecular dynamics simulations

Molecular dynamics (MD) simulations were performed over a 100 ns trajectory to validate the molecular docking findings and evaluate the dynamic stability of quinazolinone derivative **5** in complex with five critical cancer-related receptors: Topoisomerase II (5ZAD), VEGFR2 (3WZE), c-Met (3U6I), EGFR (1M17), and Estrogen Receptor α (ERα; 3ERT).

**Fig. 13:**
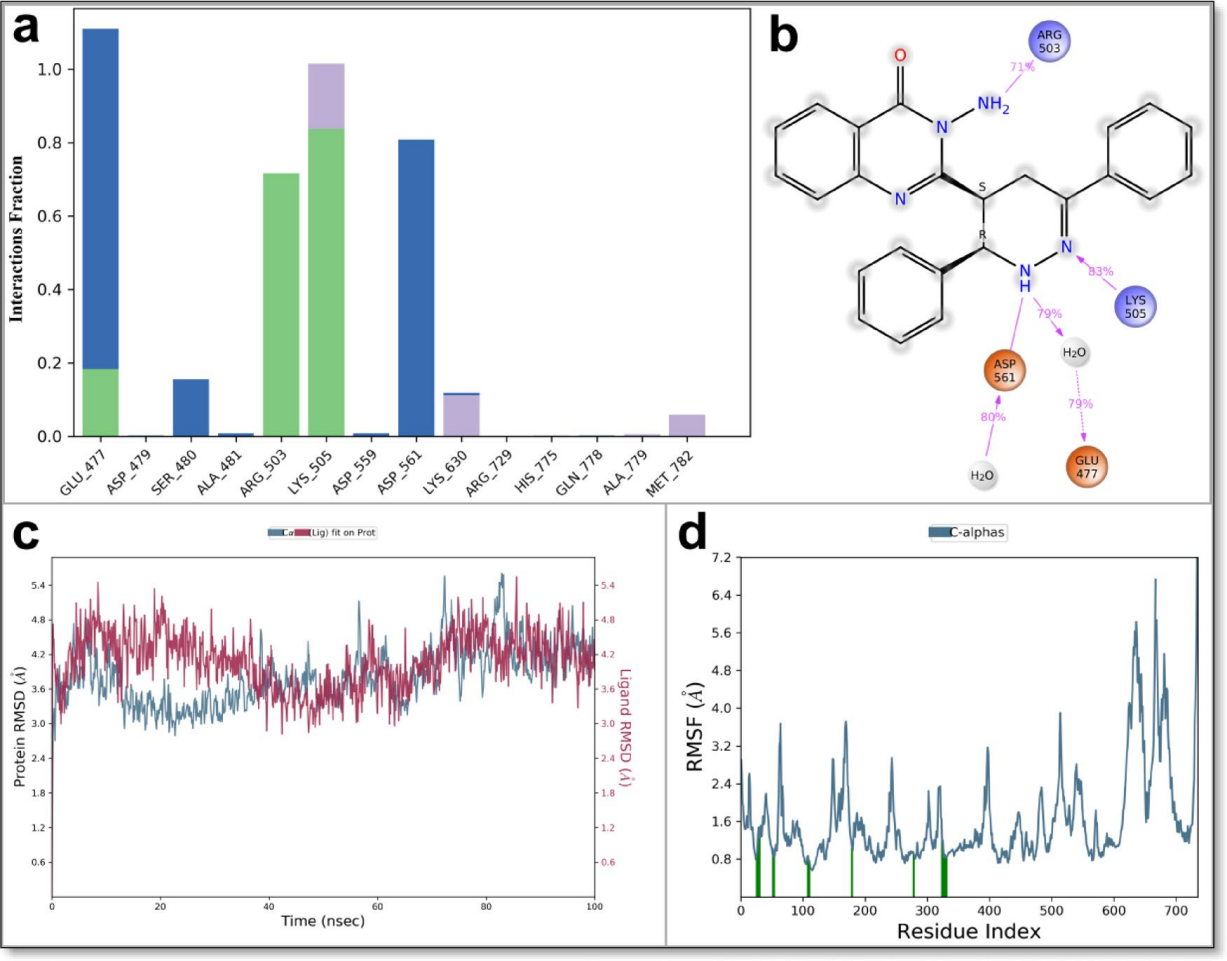
Molecular dynamics analysis of quinazolinone derivative **5** in complex with Topoisomerase II (PDB: 5ZAD). (**a**) PL-Contacts Histogram, illustrating the frequency of protein-ligand contacts over the simulation trajectory, where green indicates hydrogen bonds, violet denotes hydrophobic interactions, and blue represents water bridges. (**b**) LP-Contacts 2D-Summary, depicting the interaction hotspots between derivative **5** and key active site residues. (**c**) Protein-ligand Root Mean Square Deviation (PL-RMSD) plot, showcasing the structural stability of the ligand-receptor complex over time. (**d**) Protein Residue Root Mean Square Fluctuation (P-RMSF) profile, highlighting the flexibility of key residues within the binding pocket. These molecular dynamics simulations validate the strong binding affinity and stability of derivative **5** within the Topoisomerase II active site, supporting its potential as a promising anticancer agent.

**Fig. 14:**
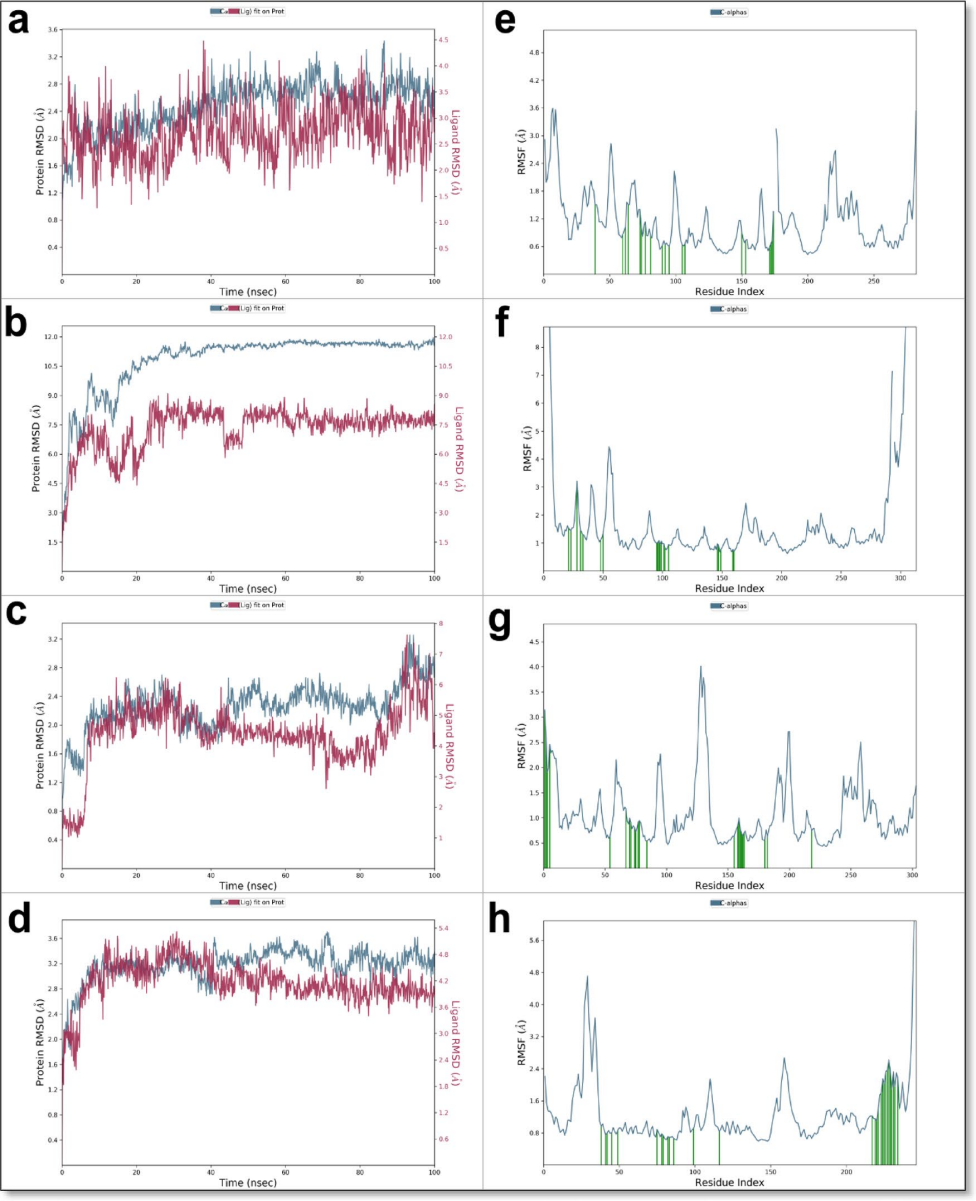
Molecular dynamics simulation analysis of quinazolinone derivative **5** with key cancer-related targets. (**a**–**d**) Root Mean Square Deviation (RMSD) plots for derivative 5 in complex with (**a**) VEGFR2 (PDB: 3WZE), (**b**) c-Met (PDB: 3U6I), (**c**) EGFR (PDB: 1M17), and (**d**) Estrogen Receptor Alpha (PDB: 3ERT), illustrating the structural stability of the ligand-receptor complexes over the simulation time. (**e**–**h**) Root Mean Square Fluctuation (RMSF) profiles for derivative **5** in complex with (**e**) VEGFR2, (**f**) c-Met, (**g**) EGFR, and (**h**) Estrogen Receptor Alpha, highlighting residue-level flexibility and dynamic behavior within the binding pockets. These results provide insights into the conformational stability and interaction dynamics of derivative **5** across multiple cancer-related targets.

The simulations provided comprehensive insights into ligand stability, receptor flexibility, and detailed binding interactions over time. The Root-Mean-Square Deviation (RMSD) analysis indicated that all protein complexes maintained structural stability within an acceptable range (1.0–3.5 Å), with slight variations among the different receptors (Figs. 13 and 14, and Supplementary Data File 3). Specifically, the ligand RMSD values in the Topoisomerase II complex ranged between 3.0 and 5.5 Å, exhibiting fluctuations around ~ 2.5 Å, suggesting a relatively stable ligand conformation within the binding pocket (Fig. 13c). In contrast, the VEGFR2-ligand complex displayed higher ligand RMSD values, primarily between 3.0 and 6.5 Å with fluctuations of approximately ~ 3.5 Å, indicating moderate flexibility but still consistent ligand occupancy within the binding site (Fig. 14a).

Similarly, the ligand bound to the c-Met receptor demonstrated RMSD fluctuations between 1.5 and 4.5 Å with an average deviation of ~ 3.0 Å, highlighting stable yet flexible interactions within its binding pocket (Fig. 14b). The EGFR complex showed ligand RMSD values predominantly between 6.0 and 9.0 Å, also reflecting fluctuations of approximately ~ 3.0 Å; this suggests notable ligand mobility that may result from partial instability or ligand-induced conformational adjustments within the active site (Fig. 14c). Among all studied complexes, the Estrogen Receptor α displayed the lowest RMSD fluctuations (~ 1.9 Å), ranging between 3.6 and 5.5 Å, indicating particularly stable ligand binding, likely due to the inherently dynamic yet accommodating nature of the receptor’s ligand-binding domain (Fig. 14d).

Overall, the MD simulations highlighted distinct variations in ligand stability across the targets, with ERα and Topoisomerase II exhibiting the strongest ligand retention and minimal conformational fluctuations, suggesting them as the most favorable binding targets. Conversely, VEGFR2 demonstrated the greatest ligand movement, indicating opportunities for further ligand optimization to enhance binding stability.

The Root-Mean-Square Fluctuation (RMSF) analysis provided additional insights into protein flexibility, identifying specific residue fluctuations within each complex. Across all five targets, ligand-binding sites remained consistently stable, with moderate RMSF values predominantly localized to flexible loop regions. Residues directly involved in ligand binding exhibited low RMSF values, confirming robust and stable interactions during the simulation. For Topoisomerase II, moderate fluctuations were observed near the DNA-binding region, reflecting inherent functional flexibility necessary for its catalytic activity (Fig. 13d). VEGFR2 and c-Met displayed slight increases in RMSF values, mostly within loops adjacent to their ligand-binding pockets, yet their core secondary structures remained structurally stable (Fig. 13e and f). EGFR and ERα complexes presented somewhat higher RMSF values localized to loop regions, suggesting these proteins undergo ligand-induced structural adaptations that support stable ligand binding (Fig. 13g and h).

**Fig. 15:**
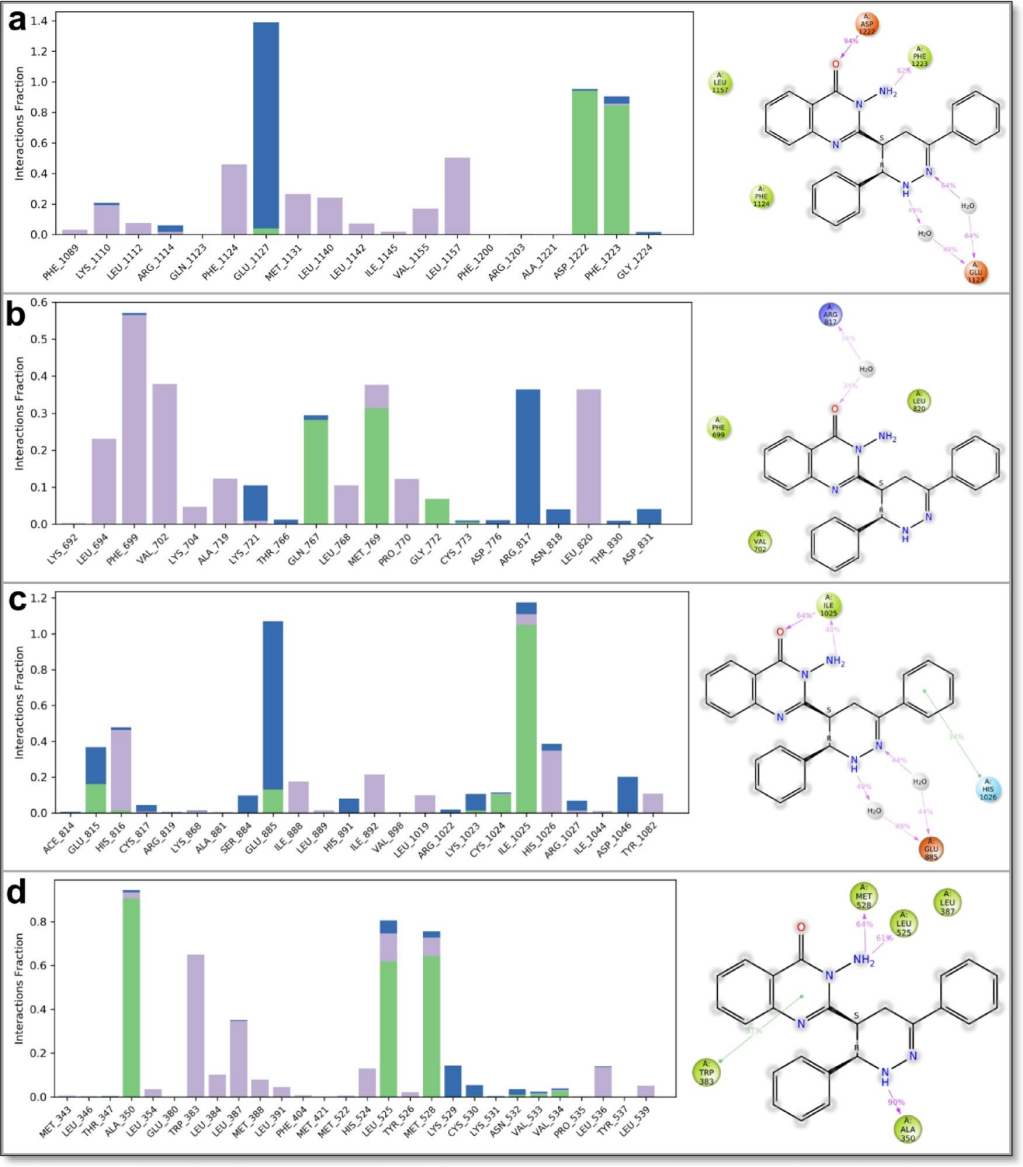
Molecular dynamics simulation analysis of quinazolinone derivative **5** with key cancer-related targets. (**a**) Interaction of derivative **5** with VEGFR2 (PDB: 3WZE), demonstrating stable binding through hydrogen bonding and hydrophobic interactions. (**b**) Interaction of derivative **5** with c-Met (PDB: 3U6I), showing key molecular contacts within the active site. (**c**) Interaction of derivative **5** with EGFR (PDB: 1M17), highlighting π-π stacking and hydrogen bonding with critical residues. (**d**) Interaction of derivative **5** with Estrogen Receptor Alpha (PDB: 3ERT), revealing key interactions that influence ligand binding stability. The accompanying histogram represents interaction analysis, where green indicates hydrogen bonds, violet denotes hydrophobic interactions, and blue represents water bridges. These molecular dynamics results reinforce the potential of derivative **5** as a multi-target anticancer agent.

The detailed protein–ligand interaction analysis, clearly illustrated in Figs. 13a–b and 15a–d, revealed sustained interactions crucial for the dynamic stability and binding strength of quinazolinone derivative 5 within each receptor’s active site. Within the Topoisomerase II complex, ligand stability was predominantly mediated through consistent hydrogen bonds involving residues GLU477, LYS505, and ARG503, significantly supported by water-mediated interactions with GLU477 and ASP561. Hydrophobic interactions played a complementary but less prominent role in ligand orientation. The VEGFR2 complex demonstrated robust and sustained hydrogen bonding, particularly with residues ILE1025, GLU815, and GLU885, reinforced by structured water bridges involving GLU885 and GLU815. Notably, substantial hydrophobic interactions with residues HIS816 and HIS1026 further stabilized the ligand conformation, suggesting its favorable inhibitory potential against VEGFR2-mediated pathways. In the c-Met complex, stable hydrogen bonds involving residues ASP1222 and PHE1223, complemented by prominent hydrophobic interactions with LEU1157, PHE1124, MET1131, and LEU1140, were essential for maintaining the ligand’s binding pose. Additionally, water bridges involving GLU1127 further enhanced ligand stability.

For EGFR, the ligand predominantly formed stable hydrophobic contacts with residues PHE699, VAL702, LEU820, and LEU694, supported by consistent hydrogen bonding interactions with MET769 and GLN767, and water-mediated bridges involving ARG817, collectively contributing to stable ligand occupancy despite its higher mobility. Similarly, within the ERα complex, significant hydrophobic interactions with residues TRP383, LEU387, and HIS524 were accompanied by persistent hydrogen bonds involving ALA350, LEU525, and MET528, emphasizing a highly stable and energetically favorable ligand-binding orientation. Notably, ionic interactions were absent in all complexes throughout the simulations, underscoring the predominant roles of hydrogen bonding, hydrophobic interactions, and water bridges in stabilizing ligand–protein interactions. Ligand torsional analysis consistently indicated minimal conformational strain, with energetically favorable torsion angles adopted by derivative 5, further supporting its potent and stable binding modes across all protein targets.

Overall, the molecular dynamics simulations, supported by clear graphical representations (Figs. 13, 14 and 15) and Supplementary Data File 3, reinforced quinazolinone derivative **5**’s potential as a promising multi-target anticancer candidate. The simulations highlighted ERα and Topoisomerase II as particularly promising targets due to their demonstrated stable ligand interactions, while VEGFR2, exhibiting greater ligand mobility, emerged as a candidate for further structural refinement. These findings provide valuable structural and dynamic insights, guiding subsequent optimization and experimental validation efforts aimed at developing effective inhibitors targeting cancer progression and metastasis.

.

## Pharmacokinetic properties and drug-likeness assessment

To assess the drug-likeness and pharmacokinetic profiles of the synthesized quinazolinone derivatives, in silico ADME predictions were carried out using SwissADME. The analysis included key parameters such as molecular weight (MW), topological polar surface area (TPSA), lipophilicity (Log P via iLOGP), gastrointestinal (GI) absorption, blood–brain barrier (BBB) permeability, P-glycoprotein (P-gp) substrate prediction, and bioavailability scores (Table 4) and Supplementary Data File 2.

Most of the compounds (specifically derivatives **2–10**) demonstrated favorable oral bioavailability with a score of 0.55, indicating good absorption and drug-like characteristics. However, compounds **9**, **11**, **14**, and **15** exhibited significantly lower scores (0.17), primarily due to higher molecular weights and/or TPSA values that exceeded standard thresholds defined by Lipinski’s and Veber’s rules. These violations suggest reduced drug-likeness and limited oral bioavailability for these higher-weight molecules.

Regarding gastrointestinal absorption, all compounds except **14** and **15** showed high GI absorption, reinforcing their potential for oral delivery. BBB permeability was limited for most derivatives, with only compounds **4**, **5**, and **10** predicted to cross the blood–brain barrier. This may reduce the risk of CNS-related side effects for the remaining derivatives, which could be advantageous depending on the therapeutic target.

P-gp substrate prediction further elucidated the compounds’ potential for cellular retention. Derivatives **4**, **5**, and **6** were predicted to be P-gp substrates, suggesting they may be subject to active efflux mechanisms that could lower their intracellular concentrations. In contrast, compound **3** was not predicted as a P-gp substrate, a property that may contribute to its strong cytotoxic activity by allowing better intracellular retention and sustained target engagement.

**Fig. 16:**
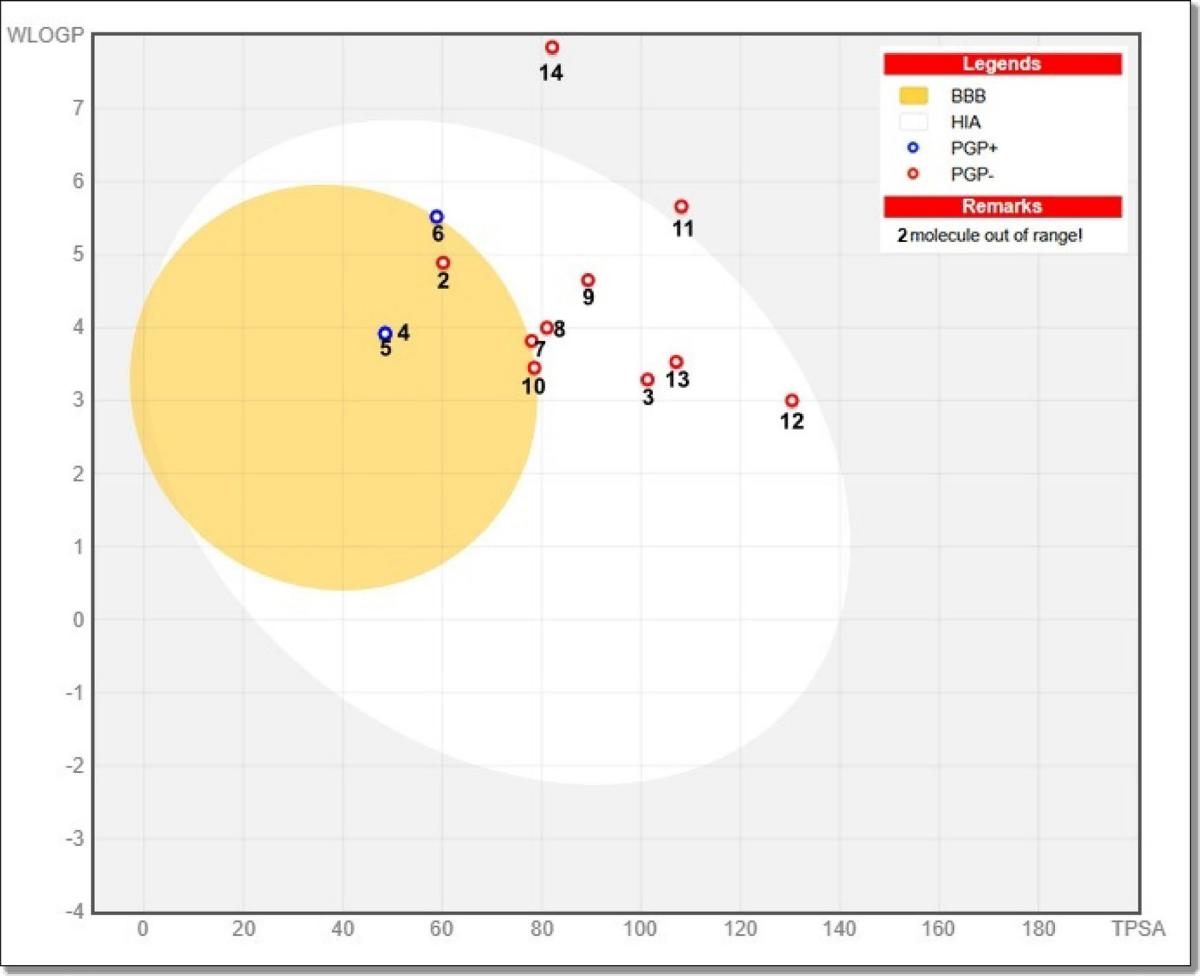
BOILED-Egg plot depicting the passive gastrointestinal absorption (HIA, white region) and blood-brain barrier (BBB, yellow region) penetration potential of quinazolinone derivatives. Each point represents a molecule plotted by its WLOGP and TPSA values. Blue circles indicate predicted P-gp substrates (PGP+), while red circles indicate non-substrates (PGP−). One compound lies outside the ideal absorption/permeability space, suggesting limited bioavailability.

The BOILED-Egg plot (Fig. 16) visually summarizes passive absorption and BBB penetration potential. Most compounds are located within the white region, indicative of favorable human intestinal absorption (HIA), while compounds falling within the yellow zone also exhibit predicted BBB permeability. Compounds **14** and **15** are plotted outside both regions, confirming their poor predicted bioavailability. The red and blue circular annotations reflect predicted P-gp substrate status, aligning with the tabulated data.

Altogether, the ADME predictions correlate well with the experimental cytotoxicity results. Notably, compounds **3** and **5** emerge as the most promising drug-like candidates, exhibiting optimal bioavailability, non-P-gp substrate behavior (compound **3**), good GI absorption, and strong cytotoxic activity. These favorable profiles are further supported by their ideal placement within the BOILED-Egg model’s absorption-permeability region.

These findings suggest that compounds **3** and **5** merit prioritization for further structural optimization, in vivo validation, and preclinical development as potential anticancer agents. Meanwhile, derivatives with suboptimal ADME profiles (e.g., **14** and **15**) may require chemical modification to improve their pharmacokinetic properties.

**Table 4:** Key ADME parameters of Quinazolinone derivatives.

Molecule	Molecular Weight (g/mol)	TPSA (Ã…Â^2^)	Log *P* (iLOGP)	GI absorption	BBB permeability	*P*-gp substrate	Bioavailability score
2	367.4	60.17	3.3	High	Yes	No	0.55
3	399.44	101.29	2.23	High	No	No	0.55
4	364.42	48.51	3.31	High	Yes	Yes	0.55
5	364.42	48.51	3.31	High	Yes	Yes	0.55
6	364.4	58.89	3.13	High	No	Yes	0.55
7	381.43	77.98	3.11	High	No	No	0.55
8	423.46	81.06	3.15	High	No	No	0.55
9	511.53	89.34	3.45	High	No	No	0.17
10	423.46	78.51	2.93	High	Yes	No	0.55
11	516.61	108.11	3.88	Low	No	No	0.17
12	442.47	130.39	2.2	High	No	No	0.55
13	424.45	107.08	2.89	High	No	No	0.55
14	611.69	82.14	4.94	Low	No	No	0.17
15	653.73	91.37	4.94	Low	No	No	0.17

This table summarizes selected Pharmacokinetic properties predicted using swissadme. Parameters include molecular weight (MW), topological Polar surface area (TPSA), lipophilicity (Log P via iLOGP), Gastrointestinal (GI) absorption, blood-brain barrier (BBB) permeability, P-glycoprotein (P-gp) substrate prediction, and estimated oral bioavailability score.

## Structure–activity relationship (SAR) analysis

A closer look at the structural variations among the synthesized quinazolinone derivatives reveals several molecular features that appear to influence their cytotoxic profiles. Among all compounds tested, derivatives **3** and **5** stood out, showing the most potent activity. Compound **3** exhibited IC_50_ values of 3.84 µM against MCF7 and 7.95 µM against HEPG-2, while compound **5** showed IC_50_ values of 4.48 µM against MCF7 and 6.90 µM against HEPG-2. These promising results are likely linked to the presence of hydrazide, lactam, and lactim groups, which are known to promote strong binding via hydrogen bonding and π–π interactions. Notably, compound **3** can exist in a lactam–lactim tautomeric equilibrium, which might provide added structural flexibility and facilitate its interaction with diverse targets like Topoisomerase II and VEGFR2.

Physicochemical data further support the observed activity. Compounds **3** and **5** both show TPSA values below 102 Å^2^ and Log P values between 2.2 and 3.3, placing them within optimal ranges for passive membrane diffusion and potential oral bioavailability. Consistent with this, the BOILED-Egg model predicts high gastrointestinal absorption for both, and even blood–brain barrier permeability in the case of compound **5**, pointing to favorable pharmacokinetic behavior.

Several other compounds, namely **8**, **10**, **12**, and **13**, also showed notable cytotoxic effects. These molecules contain acetamido, acetyl, or urea groups, which likely enhance their polarity and interaction potential with biological targets. Supporting this, docking studies showed stable hydrogen bonding and hydrophobic contacts within key cancer-related proteins, including VEGFR2, EGFR, and c-Met. It seems likely that their activity results from a combination of well-placed functional groups and acceptable drug-like properties.

On the other hand, compounds **14** and **15**, which feature bulkier pyrazole-based moieties, demonstrated weaker cytotoxicity and poor GI absorption predictions. Their relatively high molecular weights (over 600 g/mol) and Log P values around 4.94 suggest limited membrane permeability and higher susceptibility to efflux. This is in line with their BOILED-Egg positioning (outside optimal zones) and lower predicted bioavailability, hinting that excessive hydrophobicity and steric hindrance may reduce biological activity.

P-glycoprotein (P-gp) predictions also help explain some of the results. Compounds **4**, **5**, and **6** were flagged as likely P-gp substrates, meaning they may be actively exported from cells, limiting their intracellular concentrations. By contrast, compound **3** was not predicted to be a substrate, which may partly explain its strong cytotoxic performance—likely due to better intracellular retention.

Altogether, these findings emphasize the need to balance molecular weight, lipophilicity, hydrogen bonding potential, and efflux susceptibility when designing quinazolinone-based anticancer agents. Compounds **3** and **5**, in particular, offer a promising combination of structural, pharmacokinetic, and biological properties, making them strong candidates for further optimization and preclinical development.

## Conclusion

In this study, we successfully synthesized a novel series of quinazolinone derivatives and evaluated their potential as anticancer agents through comprehensive in vitro cytotoxicity assays and in silico molecular modeling. The findings demonstrated that compounds **3** and **5** exhibited the most potent cytotoxic activities against MCF7 and HEPG-2 cancer cell lines, surpassing or approaching the efficacy of the reference drug doxorubicin. Notably, compounds **8** and **10** also displayed strong cytotoxic effects, underscoring the importance of key functional groups such as hydrazide, lactam, and acetamido moieties in enhancing biological activity.

Molecular docking and molecular dynamics simulations provided mechanistic insights into the binding interactions of these derivatives with key cancer-related targets, including Topoisomerase II, VEGFR2, c-Met, EGFR, and Estrogen Receptor Alpha (ERα). Compound **5**, in particular, demonstrated strong and stable interactions across multiple targets, reinforcing its potential as a multi-target anticancer agent. These computational findings were further validated by molecular dynamics simulations, which confirmed the stability of these interactions and the structural compatibility of the compounds with their respective protein targets.

Overall, this study highlights the promising anticancer potential of quinazolinone derivatives particularly compounds **3** and **5** which warrant further structural optimization and preclinical investigation. Future work will focus on mechanistic studies, enzymatic inhibition assays, and in vivo evaluations to assess their therapeutic efficacy and pharmacokinetic profiles. These efforts will be essential for advancing the development of quinazolinone-based anticancer agents toward clinical application.

## Experimental

All melting points are uncorrected and were determined using a Griffin and George melting point apparatus (Griffin & George Ltd., Wembley, Middlesex, UK). Infrared (IR) spectra were recorded at the Faculty of Science, Ain Shams University, using the KBr pellet technique on a Pye Unicam SP1200 spectrophotometer (Pye Unicam Ltd., Cambridge, UK). ^1^H NMR spectra were obtained using a Varian Gemini 300 MHz or a Bruker Avance III spectrometer, with tetramethylsilane (TMS) as the internal standard; chemical shifts are reported in parts per million (ppm). ^13^C NMR spectra were recorded at 75 MHz at the Faculty of Science, Cairo University.

Elemental analyses were performed using a Perkin-Elmer 2400 CHN elemental analyzer (Waltham, MA, USA) at the Microanalytical Unit, Faculty of Science, Ain Shams University. All compounds gave satisfactory analytical results within ± 0.4% of theoretical values. Thin-layer chromatography (TLC) was used to monitor reaction progress and purity, employing aluminum-backed silica gel 60 F254 plates (Merck). The starting material, 3-benzylidene-5-phenylfuran-2(3*H*)-one (compound **1**), was synthesized according to a previously reported method in the literature^63^.

### 2-(4-Oxo-1,4-diphenylbut-1-en-2-yl)-4 H-benzo[d][1,3]oxazin-4-one (2)

Furanone **1** (1 g, 0.004 mol) and anthranilic acid (0.55 g, 0.004 mol) were mixed and fused in a sand bath at 160 °C for 2 h. After cooling, the resulting solid product was treated with boiling methanol, filtered, and recrystallized from an ethanol/dioxane mixture to yield compound **2** as pale-yellow crystals (74%), m.p. 178–180 °C. FTIR (KBr) cm^−1^: 3061, 3027 (CH_aryl_), 2967, 2926 (CH_alkyl_), 1742 (C=O, oxazinone), 1710 (C=O, ketone), 1656 (C=N), 1603 (C=C). ^1^H-NMR (DMSO-*d*^*6*^) δ ppm: 3.47 (d, 1H, CH_2_, *J* = 18.3 Hz), 3.95 (d, 1H, CH_2_, *J* = 18.3 Hz), 7.26–7.64 (m, 12 H, Ar-H + CH=), 7.78 (t, 1H, Ar-H, *J* = 8.1 Hz), 7.86 (d, 1H, Ar-H, *J* = 7.8 Hz), 8.19 (d, 1H, Ar-H, *J* = 8.1 Hz). ^13^C-NMR (DMSO-*d*^*6*^) δ ppm: 41.49, 94.36, 116.89, 121.26, 124.81, 125.75, 125.99, 128.88, 129.14, 129.78, 130.20, 133.93, 134.36, 135.85, 136.75, 140.44, 161.21, 166.20. MS, m/z (%): 367 (M^+^., 15.69), 366 (M-1, 33.16), 351 (42.68), 318 (22.92), 282 (22.23), 262 (28.43), 253 (27.01), 237 (28.67), 217 (19.54), 203 (100), 191 (23.66), 183 (27.18), 169 (24.25), 156 (49.02), 144 (50.62), 135 (20.04), 120 (31.69), 107 (67.47), 95 (24.52), 88 (35.04), 75 (29.28), 60 (23.86), 52 (33.82), 40 (49.52). Anal. calcd for C_24_H_17_NO_3_ (367.40): C, 78.46; H, 4.66; N, 3.81. Found C, 78.54; H, 4.69; N, 3.77%.

### 2-Benzylidene-N-[2-(hydrazinecarbonyl)phenyl]-4-oxo-4-phenylbutanamide (3)

A solution of compound **2** (1 g, 0.002 mol) in dioxane (20 mL) was stirred with hydrazine hydrate (0.4 mL, 0.006 mol) at room temperature for 8 h. The excess solvent was allowed to evaporate at ambient temperature; the resulting precipitated solid was filtered and recrystallized from ethanol to obtain compound **3** as white crystals (92%), m.p. 138–140 °C. FTIR (KBr) cm^−1^: 3338, 3315 (NH_2_), 3244, 3145 (NH), 3060 (CH_aryl_), 2864 (CH_alkyl_), 1680 (C=O, ketone), 1649 (C=O amide), 1615 (C=C). ^1^H-NMR (DMSO-*d*^*6*^) δ ppm: for (Lactam form): δ 3.43 (d, 1H, CH_2,_ *J* = 16.2 Hz), 3.50 (d, 1H, CH_2,_ *J* = 17.1 Hz), 4.53 (br.s, 2 H, NH_2_, exchangeable), 7.13–8.52 (m, 15 H, Ar-H + CH=), 9.90 (br.s, 1H, NH_2_*NH*CO, exchangeable), 10.20 (br.s, 1H, Ph*NH*CO, exchangeable, 54%). For (Lactim form): δ 3.43 (d, 1H, CH_2,_ *J* = 16.2 Hz), 3.50 (d, 1H, CH_2,_ *J* = 17.1 Hz), 4.53 (br.s, 2 H, NH_2_, exchangeable), 7.13–8.52 (m, 15 H, Ar-H + CH=), 9.90 (br.s, 1H, NH_2_*NH*CO, exchangeable), 12.30 (br.s, OH, exchangeable, 46%). ^13^C-NMR (DMSO-*d*^*6*^) δ ppm: 37.60, 81.68, 109.98, 110.99, 113.76, 117.16, 118.75, 118.94, 119.88, 120.00, 120.33, 120.89, 121.48, 121.75, 123.15, 124.23, 124.69, 125.70, 126.05, 126.55, 127.18, 130.32, 134.55, 157.36, 158.32, 158.55, 158.55, 158.66, 188.55. MS, m/z (%): 399 (M^+^., 32.69), 397 (M-2, 36.46), 394 (43.79), 377 (25.48), 374 (68.94), 363 (68.53), 358 (67.71), 346 (41.54), 343 (25.95), 329 (25.60), 326 (22.29), 299 (16.03), 291 (71.78), 276 (20.21), 270 (46.62), 255 (50.58), 252 (25.35), 234 (21.13), 209 (22.09), 198 (26.38), 196 (59.05), 186 (100), 168 (34.30), 159 (30.65), 137 (33.75), 132 (38.07), 106 (21.80), 85 (32.42), 77 (41.11), 65 (77.16), 57 (81.38), 44 (20.25). Anal. calcd for C_24_H_21_N_3_O_3_ (399.45): C, 72.17; H, 5.30; N, 10.52. Found C, 72.20; H, 4.99; N, 10.32%.

### 4-Benzyl-2-phenyl-10 H-pyridazino[6,1-b]quinazolin-10-one (4)

#### 3-Amino-2-(3,6-diphenyl-2,3,4,5-tetrahydropyridazin-4-yl)quinazolin-4(3 H)-one (5)

A solution of compound **2** (1 g, 0.002 mol) in dioxane (25 mL) was refluxed with hydrazine hydrate (0.2 mL, 0.006 mol) for 3 h. The excess solvent was allowed to evaporate at room temperature, and the resulting product was crystallized using a benzene/ethanol mixture, which gave compound **4** as white crystals (60%), m.p. 210–211 °C. The mother liquor afforded another product, compound **5**, as white crystals (40%), m.p. 222–224 °C.

### Data of compound 4

FTIR (KBr) cm^−1^: 3059, 3034 (CH_aryl_), 2930 (CH_alkyl_), 1689 (C=O), 1627 (C=N). ^1^H-NMR (DMSO-*d*^*6*^) δ ppm: 4.34 (s, 2 H, CH_2_), 7.18–8.03 (m, 14 H, Ar-H + CH=), 8.28 (d, 1H, Ar-H, *J* = 8.1 Hz). ^13^C-NMR (DMSO-*d*^*6*^) δ ppm: 36.08, 111.72, 114.19, 115.67, 119.08, 121.17, 123.38, 124.93, 126.43, 126.68, 127.12, 127.59, 128.54, 129.26, 130.98, 134.31, 135.11, 138.16, 144.90, 146.45, 146.80, 150.00, 158.14. Anal. calcd for C_24_H_17_N_3_O (363.42): C, 79.32; H, 4.72; N, 11.56. Found C, 79.20; H, 4.70; N, 11.40%.

### Data of compound 5

FTIR (KBr) cm^−1^: 3311, 3284, 3195 (NH, NH_2_), 3086, 3059 (CH_aryl_), 2959, 2826 (CH_alkyl_), 1680 (C=O), 1619 (C=N). ^1^H-NMR (DMSO-*d*^*6*^) δ ppm: 2.69 (dd, 1H, CH_2_, *J* = 4.2, 17.7 Hz), 2.87 (dd, 1H, CH_2,_ *J* = 12, 17.7 Hz), 4.26 (td, 1H, PhCH*CH*, *J* = 11.7, 4.8 Hz), 5.25 (d, 1H, Ph*CH*CH, *J* = 3.9 Hz), 5.83 (br.s, 2 H, NH_2_, exchangeable), 6.76–7.73 (m, 13H, Ar-H), 8.14 (d, 1H, Ar-H, *J* = 7.8 Hz), 8.24 (br.s, 1H, NH, exchangeable). ^13^C-NMR (DMSO-*d*^*6*^) δ ppm: 21.65, 36.65, 55.27, 119.85, 123.71, 125.90, 126.46, 126.65, 126.77, 127.24, 127.57, 128.28, 134.07, 136.53, 139.01, 141.23, 145.97, 156.52, 161.19. Anal. calcd for C_24_H_21_N_5_O (395.47): C, 72.89; H, 5.35; N, 17.71. Found C, 72.87; H, 5.32; N, 17.68%.

### 2-(2,5-Diphenylfuran-3-yl)quinazolin-4(3 H)-one (6)

A mixture of compound **2** (1 g, 0.002 mol) and ammonium acetate (5 g, 0.064 mol) was heated in a sand bath at 160 °C for 1 h. The reaction mixture was then allowed to cool to ambient temperature before being poured into ice-cold water; the resulting solid was filtered and recrystallized from ethanol to yield compound **6** as brown crystals (73%), m.p. 227–229 °C. FTIR (KBr) cm^−1^: 3151, 3130 (NH), 3085, 3058 (CH_aryl_), 1692 (C=O), 1610 (C=N). ^1^H-NMR (DMSO-*d*^*6*^) δ ppm: 6.90 (s, 1H, Ar-H), 7.21 (s, 1H, Ar-H), 7.40–7.50 (m, 9 H, Ar-H), 7.82 (d, 2 H, Ar-H, *J* = 7.5 Hz), 7.90 (d, 2 H, Ar-H, *J* = 7.8 Hz), 10.57 (br.s,1H, NH, exchangeable). ^13^C-NMR (DMSO-*d*^*6*^) δ ppm: 97.32, 125.59, 128.80, 129.03, 129.46, 129.60, 129.70, 129.96, 130.34, 131.01, 135.49, 145.91, 170.99. Anal. calcd for C_24_H_16_N_2_O_2_ (364.40): C, 79.11; H, 4.43; N, 7.69. Found C, 79.32; H, 4.52; N, 7.42%.

### 3-Amino-2-(4-oxo-1,4-diphenylbut-1-en-2-yl)quinazolin-4(3 H)-one (7)

#### 3-Acetamido-2-(4-oxo-1,4-diphenylbut-1-en-2-yl)quinazolin-4(3 H)-one (8)

When compound **3** (1 g, 0.002 mol) was heated in acetic acid under reflux for 3 h, a white solid precipitated after most of the solvent had evaporated at room temperature. Fractional crystallization of the formed solid using a benzene/ethanol mixture gave compound 7 as white crystals (55%), m.p. 239–240 °C. The mother liquor afforded another compound, compound **8**, as white crystals (45%), m.p. 267–269 °C.

### Data of compound 7

FTIR (KBr) cm^−1^: 3302, 3206, 3106 (NH_2_), 3071, 3029 (CH_aryl_), 2978 (CH_alkyl_), 1700 (C=O, ketone), 1661 (C=O, amide), 1639 (C=N), 1599 (C=C).^1^H-NMR (DMSO-*d*^*6*^) δ ppm: 3.40 (d, 1H, CH_2_, *J* = 18.3 Hz), 4.37 (d, 1H, CH_2_, *J* = 18.3 Hz), 5.51 (br.s, 2 H, NH_2_, exchangeable), 7.17–7.29 (m, 6 H, Ar-H + CH=), 7.38–7.49 (m, 3 H, Ar-H), 7.56–7.66 (m, 4 H, Ar-H), 7.91 (d, 1H, Ar-H, *J* = 7.5 Hz), 8.14 (d, 1H, Ar-H, *J* = 8.1 Hz). ^13^C-NMR (DMSO-*d*^*6*^) δ ppm: 38.95, 80.71, 120.45, 120.61, 124.65, 125.43, 127.69, 127.84, 128.38, 128.72, 128.97, 129.54, 130.00, 132.91, 133.19, 134.40, 135.41, 142.54, 162.28, 166.21. Anal. calcd for C_24_H_19_N_3_O_2_ (381.44): C, 75.57; H, 5.02; N, 11.02. Found C, 75.42; H, 4.99; N, 11.32%.

### Data of compound 8

FTIR (KBr) cm^−1^: 3253, 3224 (NH), 3090, 3055 (CH_aryl_), 2997, 2920, 2850 (CH_alkyl_), 1724 (C=O, ketone), 1712 (C=O, amide), 1643 (C=N), 1602 (C=C). ^1^H-NMR (DMSO-*d*^*6*^) δ ppm: 2.13 (s, 3 H, CH_3_), 3.31 (d, 1H, CH_2_, *J* = 18 Hz), 3.79 (d, 1H, CH_2_, J = 18.6 Hz), 7.25–7.47 (m, 8 H, Ar-H + CH=), 7.61–7.69 (m, 5 H, Ar-H), 7.90 (d, 1H, Ar-H, *J* = 6.9 Hz), 8.20 (d, 1H, Ar-H, *J* = 7.8 Hz), 10.59 (br.s, 1H, NH, exchangeable).^13^C-NMR (DMSO-*d*^*6*^) δ ppm: 20.64, 38.49, 81.12, 120.13, 120.76, 124.65, 125.61, 127.00, 128.27, 128.72, 128.92, 129.65, 130.00, 130.18, 133.36, 134.00, 134.10, 135.65, 142.21, 160.59, 166.02, 171.47. Anal. calcd for C_26_H_21_N_3_O_3_ (423.47): C, 73.74; H, 4.99; N, 9.92. Found C, 73.69; H, 4.89; N, 9.90%.

### 2-[4-Oxo-2-(4-oxo-1,4-diphenylbut-1-en-2-yl)quinazolin-3(4 H)-yl]isoindoline-1,3-dione(9)

Compound **3** (1 g, 0.002 mol) was fused with phthalic anhydride (0.37 g, 0.002 mol) at 120 °C for 0.5 h in a sand bath. After cooling, an orange product was formed, which was recrystallized from ethanol to obtain compound **9** as orange crystals (72%), m.p. 258–260 °C. FTIR (KBr) cm^−1^: 3068 (CH_aryl_), 1795 (Asymmetric C=O, 1,3-dione), 1738 (Symmetric C=O, 1,3-dione), 1707 (C=O, ketone), 1684 (C=O, amide), 1613 (C=N), 1601 (C=C). ^1^H-NMR (DMSO-*d*^*6*^) δ ppm: for (Keto form): δ 3.56 (s, 2 H, CH_2_, 34%), 6.73 (s, 1H, CH=), 7.20–7.99 (m, 18 H, Ar-H). For (Enol form): δ 6.73 (s, 1H, CH=), 7.20–7.99 (m, 19 H, Ar-H + CH=), 11.32 (br.s, 1H, OH, exchangeable, 66%). ^13^C-NMR (DMSO-*d*^*6*^) δ ppm: 38.67, 101.26, 123.73, 127.86, 128.18, 128.27, 128.64, 128.81, 129.09, 129.17, 129.45, 129.78, 130.09, 130.18, 130.43, 130.54, 130.99, 131.55, 132.25, 135.25, 148.70, 164.97, 165.18, 165.39, 169.06. Anal. calcd for C_32_H_21_N_3_O_4_ (511.54): C, 75.14; H, 4.14; N, 8.21. Found C, 75.22; H, 4.15; N, 8.30%.

### N′-Acetyl-2-(3-benzylidene-2-oxo-5-phenyl-2,3-dihydro-1 H-pyrrol-1-yl)benzo- Hydrazide (10)

A mixture of compound **3** (1 g, 0.002 mol) and purified acetic anhydride (25 mL) was stirred at room temperature for 2 h. The resulting solid was filtered, dried, and recrystallized from benzene to yield compound **10** as yellow crystals (57%), m.p. 186–188 °C. FTIR (KBr) cm^−1^: 3238 (NH), 3034 (CH_aryl_), 1711, 1688, 1642 (C=O), 1602 (C=C). ^1^H-NMR (DMSO-*d*^*6*^) δ ppm: 1.88 (s, 3 H, CH_3_), 6.74 (s, 2 H, CH=), 6.99 (d, 2 H, Ar-H, *J* = 6.9 Hz), 7.27–7.52 (m, 9 H, Ar-H), 7.76 (d, 1H, Ar-H, *J* = 6.9 Hz), 7.86 (d, 2 H, Ar-H, *J* = 7.2 Hz), 9.91 (br.s, 1H, NH, exchangeable), 10.17 (br.s, 1H, NH, exchangeable).^13^C-NMR (DMSO-*d*^*6*^) δ ppm: 20.54, 101.01, 127.84, 128.21, 128.32, 129.13, 129.52, 129.83, 130.44, 130.49, 131.08, 131.68, 132.93, 134.45, 135.17, 148.89, 165.65, 168.36, 168.80. Anal. calcd for C_26_H_21_N_3_O_3_ (423.47): C, 73.74; H, 5.00; N, 9.92. Found C, 73.88; H, 4.95; N, 9.90%.

### 1-[4-Oxo-2-(4-oxo-1,4-diphenylbut-1-en-2-yl)quinazolin-3(4 H)-yl]-3-phenylthio- Urea (11)

A mixture of compound **3** (1 g, 0.002 mol) and phenyl isothiocyanate (0.34 g, 0.002 mol) in 10% ethanolic sodium hydroxide solution (2 g NaOH in 20 mL ethanol) was heated under reflux for 2 h. The reaction mixture was then poured into ice water and acidified with concentrated hydrochloric acid. The resulting solid was filtered, dried, and recrystallized from ethanol to obtain compound **11** as pale-yellow crystals (43%), m.p. 211–212 °C. FTIR (KBr) cm^−1^: 3517, 3325 (NH), 3060, 3028 (CH_aryl_), 2881, 2720, 2633 (CH_alkyl_), 1660 (C=O), 1608 (C=C), 1157 (C=S). ^1^H-NMR (DMSO-*d*^*6*^) δ ppm: 4.14 (s, 2 H, CH_2_), 7.03 (t, 2 H, Ar-H, *J* = 7.5 Hz), 7.22–7.63 (m, 12 H, Ar-H), 7.83 (s, 1H, CH=), 8.00-8.05 (m, 4 H, Ar-H), 9.06 (d, 1H, Ar-H, *J* = 8.7 Hz), 11.24 (br.s, 1H, NH, exchangeable), 13.60 (br.s, 1H, NH, exchangeable). ^13^C-NMR (DMSO-*d*^*6*^) δ ppm: 35.27, 114.34, 119.23, 120.27, 125.51, 125.93, 126.90, 127.02, 128.72, 128.93, 129.12, 129.17, 129.43, 130.09, 131.38, 134.37, 136.25, 136.51, 143.40, 152.79, 154.81, 170.58. Anal. calcd for C_31_H_24_N_4_O_2_S (516.62): C, 72.07; H, 4.68; N, 10.85. Found C, 72.20; H, 4.70; N, 10.92%.

### 2-[2-(2-Benzylidene-4-oxo-4-phenylbutanamido)benzoyl]hydrazine-1-carboxamide (12)

A solution of compound **3** (1 g, 0.002 mol) in AcOH/H_2_O (25 mL, 1:1) was stirred with potassium cyanate (0.2 g, 0.002 mol) at room temperature for 3 h. During stirring, an orange precipitate formed, which was then filtered and recrystallized from benzene to yield compound **12** as orange crystals (64%), m.p. 147–149 °C. FTIR (KBr) cm^−1^: 3438, 3314, 3248 (NH, NH_2_), 3054, 3030 (CH_aryl_), 1686 (C=O, ketone), 1671 (C=O, amide), 1617 (C=C). ^1^H-NMR (DMSO-*d*^*6*^) δ ppm: 3.33 (d, 1H, CH_2,_ *J* = 17.7 Hz), 3.98 (d, 1H, CH_2,_ *J* = 18 Hz), 6.33 (br.s, 2 H, NH_2,_ exchangeable), 6.50 (br.s, 1H, NH_a_, exchangeable), 7.27–7.66 (m, 13H, Ar-H + CH=), 7.93 (d, 1H, Ar-H, *J* = 7.2 Hz), 8.21 (d, 1H, Ar-H, *J* = 7.8 Hz), 8.91 (br.s, 1H, NH_b_, exchangeable), 10.20 (br.s, 1H, NH_c_, exchangeable).^13^C-NMR (DMSO-*d*^*6*^) δ ppm: 38.67, 81.37, 120.64, 124.89, 125.43, 126.46, 127.68, 128.02, 128.35, 128.88, 129.01, 129.65, 130.05, 130.58, 131.68, 132.94, 133.92, 134.33, 135.00, 135.98, 142.37, 159.48, 162.18, 166.17, 168.57. Anal. calcd for C_25_H_22_N_4_O_4_ (442.48): C, 67.86; H, 5.01; N, 12.66. Found C, 67.80; H, 4.95; N, 12.62%.

### 1-[4-Oxo-2-(4-oxo-1,4-diphenylbut-1-en-2-yl)quinazolin-3(4 H)-yl]urea (13)

A solution of compound **12** (1 g, 0.002 mol) in 2 N ethanolic sodium hydroxide (2 g NaOH in 25 mL ethanol) was heated under reflux for 2 h. The reaction mixture was then poured into cold water and acidified with concentrated hydrochloric acid. The resulting product was filtered and recrystallized from ethanol to yield compound **13** as pale-yellow crystals (50%), m.p. 166–167 °C. FTIR (KBr) cm^−1^: 3517, 3300, 3228 (NH_2_), 3185 (NH), 3061, 3029 (CH_aryl_), 2969, 2891 (CH_alkyl_), 1678 (C=O), 1607 (C=C). ^1^H-NMR (DMSO-*d*^*6*^) δ ppm: 4.15 (s, 2 H, CH_2_), 5.50 (br.s, 2 H, NH_2,_ exchangeable), 7.00 (t, 1H, Ar-H, *J* = 7.5 Hz), 7.21–7.59 (m, 9 H, Ar-H), 7.79 (s, 1H, CH=), 8.00 (d, 2 H, Ar-H, *J* = 6.9 Hz), 8.07 (d, 1H, Ar-H, *J* = 6.9 Hz), 9.05 (d, 1H, Ar-H, *J* = 8.7 Hz), 11.82 (br.s, 1H, NH, exchangeable). ^13^C-NMR (DMSO-*d*^*6*^) δ ppm: 35.31, 116.25, 119.13, 120.05, 125.20, 125.85, 126.56, 126.83, 126.99, 128.42, 128.65, 128.89, 129.42, 130.08, 131.40, 133.48, 136.36, 136.65, 143.20, 152.48, 154.87, 170.98. Anal. calcd for C_25_H_20_N_4_O_3_ (424.20): C, 70.78; H, 4.75; N, 13.20. Found C, 70.75; H, 4.72; N, 13.24%.

### 3-{[(1,3-Diphenyl-1 H-pyrazol-4-yl)methylene]amino}-2-(4-oxo-1,4-diphenylbut-1-en-2-yl)-3,4-dihydroquinazolin-4(3 H)-one (14)

A mixture of compound **3** (1 g, 0.002 mol) and 1,3-diphenyl-1H-pyrazole-4-carbaldehyde (0.62 g, 0.002 mol) in 30 mL absolute ethanol was refluxed for 2 h. The reaction mixture was allowed to cool; the resulting solid was filtered, dried, and recrystallized from ethanol to obtain compound **14** as pale-yellow crystals (78%), m.p. 161–163 °C. FTIR (KBr) cm^−1^: 3056 (CH_aryl_), 2853 (CH_alkyl_), 1680 (C=O), 1648 (C=N). ^1^H-NMR (DMSO-*d*^*6*^) δ ppm: for (Keto form): δ 3.50 (s, 2 H, CH_2_, 64%), 7.23–8.03 (m, 25 H, Ar-H + CH=), 8.63 (s, 1H, N=CH), 8.96 (s, 1H, CH-pyrazolyl). For (Enol form): δ 7.23–8.03 (m, 26 H, Ar-H + 2CH=), 8.63 (s, 1H, N=CH), 8.96 (s, 1H, CH-pyrazolyl), 11.94 (br.s, 1H, OH, exchangeable, 36%). ^13^C-NMR (DMSO-*d*^*6*^) δ ppm: 38.69, 46.62, 116.86, 118.85, 126.40, 127.03, 127.78, 128.03, 128.03, 128.21, 128.39, 128.66, 128.79, 128.99, 129.62, 129.77, 130.64, 131.06, 131.98, 134.97, 139.06, 151.92, 167.60. Anal. calcd for C_40_H_29_N_5_O_2_ (611.71): C, 78.54; H, 4.78; N, 11.45. Found C, 78.36; H, 4.65; N, 11.43%.

#### 3-(3-{[(1,3-Diphenyl-1 H-pyrazol-4-yl)methylene]amino}-4-oxo-3,4-dihydroquinazo- lin-2-yl)-1,4-diphenylbuta-1,3-dien-1-yl acetate (15)

A solution of compound **14** (1.5 g, 0.002 mol) in acetic acid (25 mL) was refluxed for 2 h. After cooling the reaction mixture, the precipitated solid was filtered and recrystallized from acetic acid to yield compound **15** as pale-yellow crystals (69%), m.p. 226–228 °C. FTIR (KBr) cm^−1^: 3060, 3027 (CH_aryl_), 2927 (CH_alkyl_), 1708 (C=O, ketone), 1664 (C=O, amide), 1598 (C=N). ^1^H-NMR (DMSO-*d*^*6*^) δ ppm: 1.91 (s, 3 H, CH_3_), 7.24–8.26 (m, 26 H, Ar-H + 2CH=), 9.19 (s, 1H, CH = pyrazolo), 9.27 (s, 1H, N=CH). ^13^C-NMR (DMSO-*d*^*6*^) δ ppm: 21.08, 38.67, 81.58, 116.46, 119.00, 120.17, 120.45, 124.94, 125.52, 127.24, 127.55, 128.42, 128.60, 128.69, 128.77, 128.88, 129.00, 129.66, 129.83, 130.14, 132.09, 133.16, 133.98, 134.25, 135.30, 138.96, 142.23, 152.16, 153.66, 160.76, 166.22. Anal. calcd for C_42_H_31_N_5_O_3_ (653.74): C, 77.17; H, 4.78; N, 10.71. Found C, 77.20; H, 4.66; N, 10.53%.

#### Cytotoxicity assay

The cytotoxic potential of the synthesized compounds was evaluated against two human cancer cell lines: hepatocellular carcinoma (HEPG-2) and breast adenocarcinoma (MCF-7). Both cell lines were obtained from the American Type Culture Collection (ATCC) via the Holding Company for Biological Products and Vaccines (VACSERA), Cairo, Egypt. Doxorubicin, a widely used chemotherapeutic agent, served as positive control, while untreated cells (0.1% DMSO) served as negative control.

Cytotoxicity was assessed using the MTT colorimetric assay^64^, which quantifies mitochondrial metabolic activity as an indicator of viable cells. Yellow MTT (3-(4,5-dimethylthiazol-2-yl)-2,5-diphenyltetrazolium bromide) is enzymatically reduced to purple formazan crystals by metabolically active cells.

Cells were cultured in RPMI-1640 medium supplemented with 10% fetal bovine serum (FBS), 100 U/mL penicillin, and 100 µg/mL streptomycin, and maintained at 37 °C in a humidified 5% CO_2_ atmosphere. Cells were seeded at a density of 1 × 10^4^ cells per well in 96-well plates and incubated for 48 h. Test compounds were added at various concentrations, and cells were further incubated for 24 h. All treatments, including controls, contained 0.1% DMSO.

After treatment, 20 µL of MTT solution (5 mg/mL in PBS) was added to each well and incubated at 37 °C for 4 h. The medium was carefully removed, and 100 µL of DMSO was added to solubilize the formazan crystals. Absorbance was measured at 570 nm using a microplate reader (EXL 800, USA).

Cell viability (%) was calculated relative to the negative control using the formula:

$$Cell\;Viability\left( \% \right) = \left( {\frac{{A_{{570\;treated}} - A_{{570\;blank}} }}{{A_{{570\;control}} - A_{{570\;blank}} }}} \right)*100$$

All experiments were conducted in triplicate wells and repeated independently three times (*n* = 3 biological replicates). Viability data at each concentration were pooled and fitted to a four-parameter logistic (4PL) nonlinear regression model for dose–response analysis. IC_50_ values were derived from the resulting regression curves and are reported as mean ± standard error (SE) based on the model fit.

## Molecular docking assessment

Molecular docking studies were performed to evaluate the binding interactions of 14 synthesized quinazolinone derivatives against five key cancer-related protein targets using Molecular Operating Environment (MOE) 2024.06. The selected targets included Topoisomerase II (PDB ID: 5ZAD), Vascular Endothelial Growth Factor Receptor 2 (VEGFR2, PDB ID: 3WZE), Hepatocyte Growth Factor Receptor (c-Met, PDB ID: 3U6I), Epidermal Growth Factor Receptor (EGFR, PDB ID: 1M17), and Estrogen Receptor Alpha (ERα, PDB ID: 3ERT). To validate the docking results, five clinically approved reference inhibitors—doxorubicin, sorafenib, crizotinib, gefitinib, and 4-hydroxytamoxifen—were used as controls, each selectively targeting one of the respective proteins. The docking study aimed to assess whether the synthesized quinazolinone derivatives could establish binding interactions comparable to, or better than, these reference inhibitors.

The crystal structures of the selected receptors were retrieved from the Protein Data Bank (PDB) and prepared for docking using MOE 2024.06. Unlike conventional docking protocols, the co-crystallized ligands were retained in the active sites to enable direct comparative analysis between the synthesized derivatives and the native ligands. Receptor preparation involved protonation at physiological pH using the Protonate 3D tool, followed by energy minimization to eliminate steric clashes while preserving the receptor’s structural integrity. The docking site was explicitly defined based on the coordinates of the co-crystallized ligand to ensure accurate evaluation of ligand–receptor interactions.

A library of 14 quinazolinone derivatives was prepared by energy minimization using the MMFF94x force field. Protonation states were adjusted to physiological pH to maintain proper charge distribution, and the optimized ligand structures were saved in SDF format for docking simulations^65–70^. Docking was performed in MOE 2024.06 using the Triangle Matcher algorithm for ligand placement, followed by Rigid Receptor refinement to maintain receptor stability. The London dG scoring function was used for initial scoring, while GBVI/WSA dG rescoring was applied to account for solvation effects. For each ligand, 100 docking poses were generated, and the top 30 poses were selected for further analysis^65–70^.

Docking results were stored in an .mdb database for systematic analysis. Molecular interaction fingerprints were generated to evaluate hydrogen bonding, hydrophobic interactions, and π–π stacking between the tested compounds and their respective targets. Comparative binding pose visualization was also performed to examine similarities and differences in interactions relative to reference inhibitors and co-crystallized ligands. All docking simulations and analyses were carried out using MOE 2024.06, ensuring a comprehensive assessment of the potential anticancer activity of the synthesized quinazolinone derivatives.

## Molecular dynamics simulations

Molecular dynamics (MD) simulations were conducted using Desmond (Schrödinger Suite, Schrödinger, LLC, New York, NY, USA) to validate the docking results and evaluate the dynamic stability of compound 5 in complex with the five selected receptor targets: Topoisomerase II (5ZAD), VEGFR2 (3WZE), c-Met (3U6I), EGFR (1M17), and ERα (3ERT). Simulations were performed over a 100 ns trajectory under physiological conditions to provide a deeper understanding of the ligand–receptor binding behavior and conformational dynamics^66,70^.

The molecular docking studies provided the initial coordinates for each complex, as the docking procedure had already used structurally optimized protein models from the PDB. Therefore, no additional protein preparation was required. Each complex was solvated using the explicit TIP3P water model, with a 10 Å buffer around the protein to prevent edge effects. The system was neutralized with Na⁺ and Cl⁻ ions, maintaining physiological ionic strength at 0.15 M NaCl. The OPLS4 force field was used to describe molecular interactions, and ligand parameters were generated using the LigPrep module in Schrödinger.

Before the production run, the system was subjected to energy minimization using a combination of Steepest Descent (2000 steps) and Conjugate Gradient algorithms to eliminate steric clashes and stabilize the system. MD simulations were then conducted under NPT ensemble conditions, with temperature controlled at 300 K using the Nose–Hoover thermostat, and pressure maintained at 1 atm using the Martyna–Tobias–Klein barostat. Long-range electrostatic interactions were calculated using the Particle Mesh Ewald (PME) method, and a 9.0 Å cutoff was used for short-range interactions. The SHAKE algorithm was applied to constrain bond lengths involving hydrogen atoms, allowing for a 2-fs time step to improve computational efficiency^66,70^.

After the 100 ns simulation, trajectory analysis was performed using the Simulation Interaction Diagram (SID) module in Desmond. The analysis included Root Mean Square Deviation (RMSD) to assess complex stability, Root Mean Square Fluctuation (RMSF) to evaluate residue flexibility, and Radius of Gyration (Rg) to monitor the compactness of the protein–ligand complexes. In addition, hydrogen bond occupancy analysis was conducted to assess the persistence of key interactions throughout the simulation.

## Pharmacokinetic properties and drug-likeness assessment

The ADME properties of the quinazolinone derivatives were predicted using the SwissADME online tool (http://www.swissadme.ch), which provides computational estimates based on molecular descriptors and machine learning models. Canonical SMILES representations of each compound were submitted to evaluate physicochemical properties (e.g., molecular weight, hydrogen bond donors/acceptors, TPSA), lipophilicity (Log P via iLOGP), and drug-likeness filters (Lipinski, Veber rules). The BOILED-Egg model was used to predict blood-brain barrier (BBB) permeability and gastrointestinal (GI) absorption based on WLOGP vs. TPSA distribution. Additional filters included bioavailability score and identification of P-gp substrate behavior.

## Electronic supplementary material

Below is the link to the electronic supplementary material.

Supplementary Material 1

Supplementary Material 2

Supplementary Material 3

## Data Availability

All data generated or analyzed during this study are included in this published article and its supplementary information files.
