# Supplementary material for: Synthesis, comprehensive in silico studies, and cytotoxicity evaluation of novel quinazolinone derivatives as potential anticancer agents
Source: Sci Rep. 2025 Jul 3;15:23697. doi: 10.1038/s41598-025-08062-7 (PMC12229541; doi:10.1038/s41598-025-08062-7)
Supplement: Supplementary file 1 — Supplementary Material 1 [file 41598_2025_8062_MOESM1_ESM.pdf]

Copies of the spectral data for the synthesized compounds

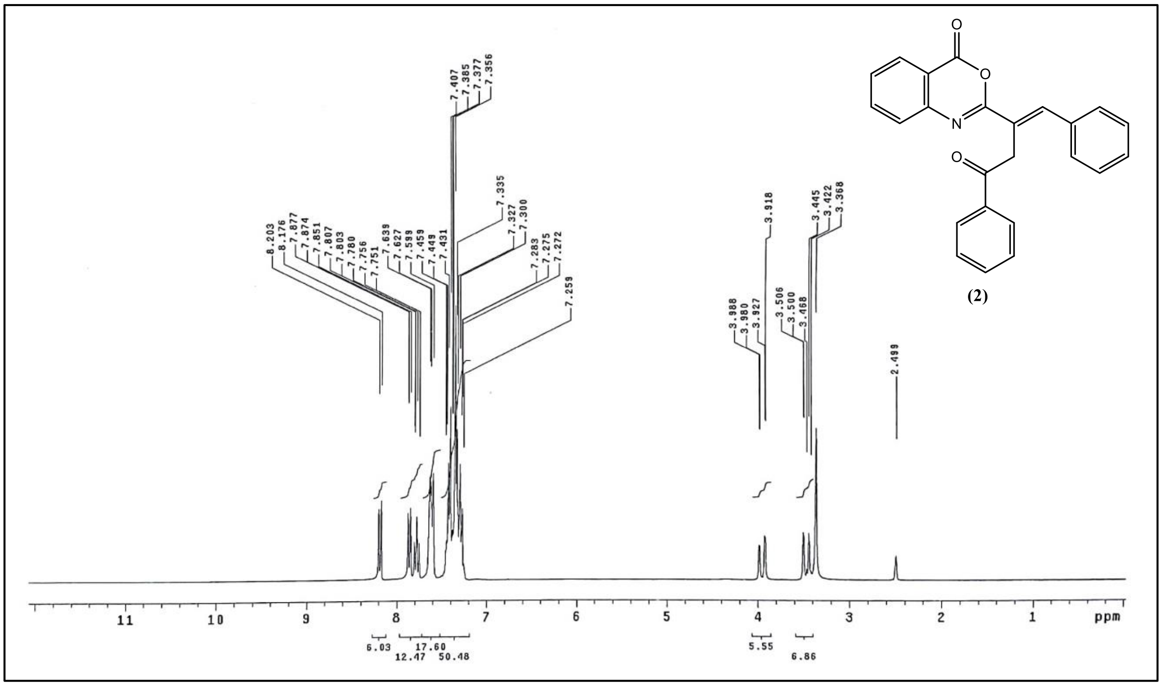

^{1}H-NMR (DMSO-*d6*) spectrum compound of **2**

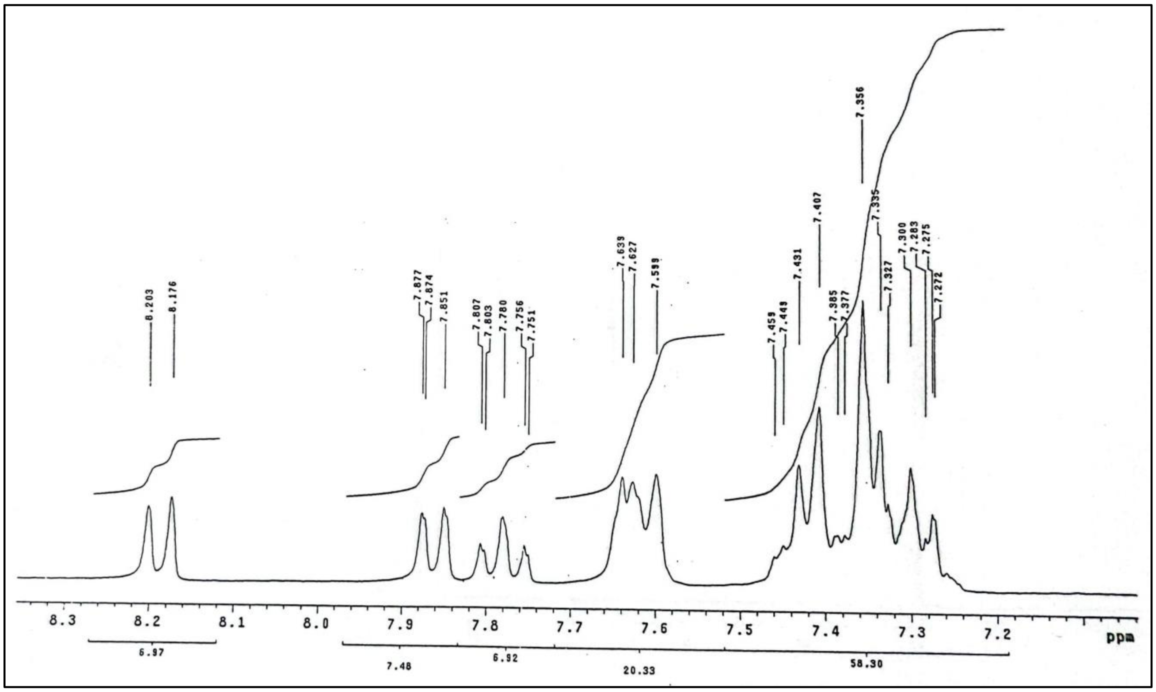

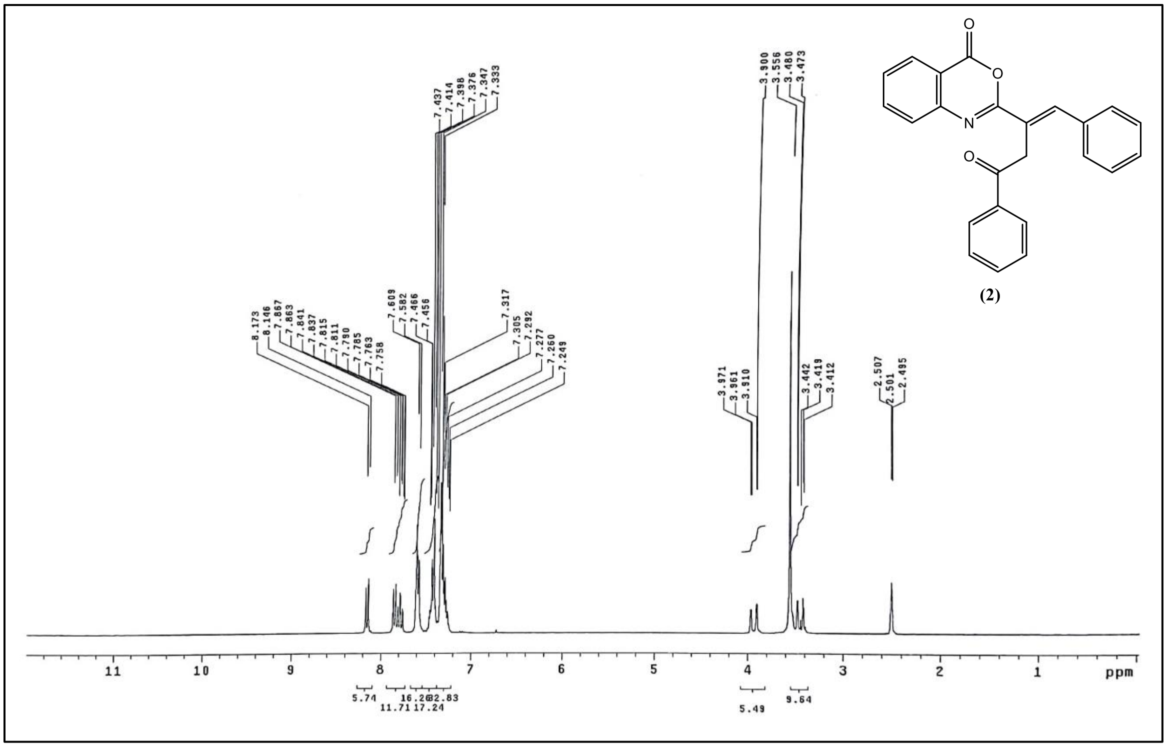

^{1}H-NMR (DMSO-*d6*) + D_2O spectrum compound of **2**

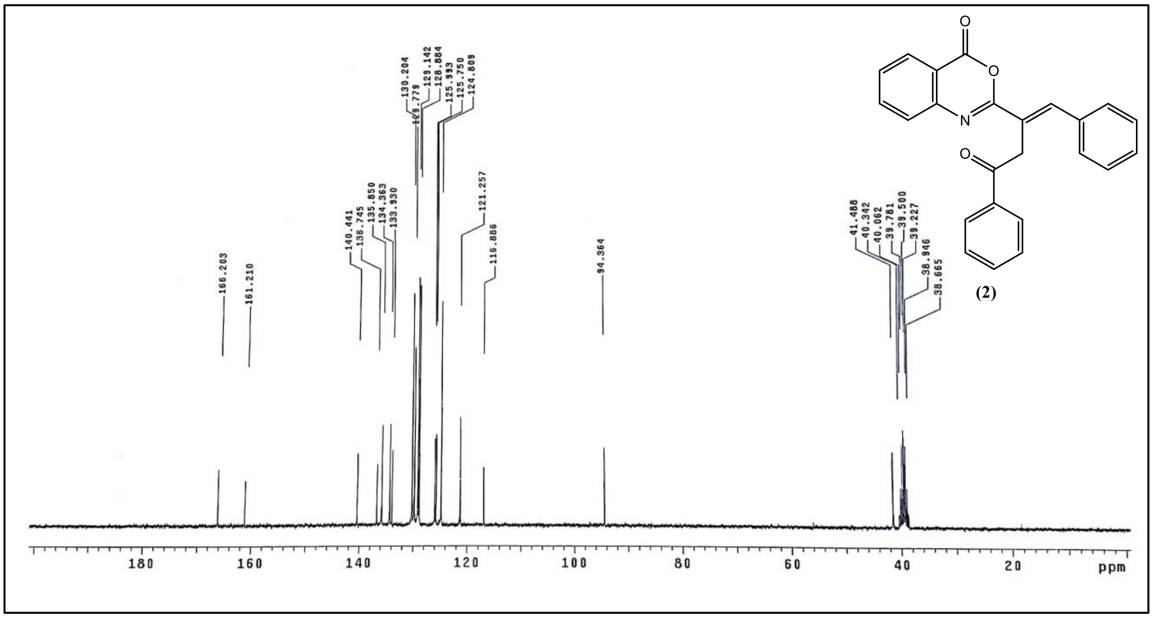

^{13}C-NMR (DMSO-d^6) spectrum compound of **2**

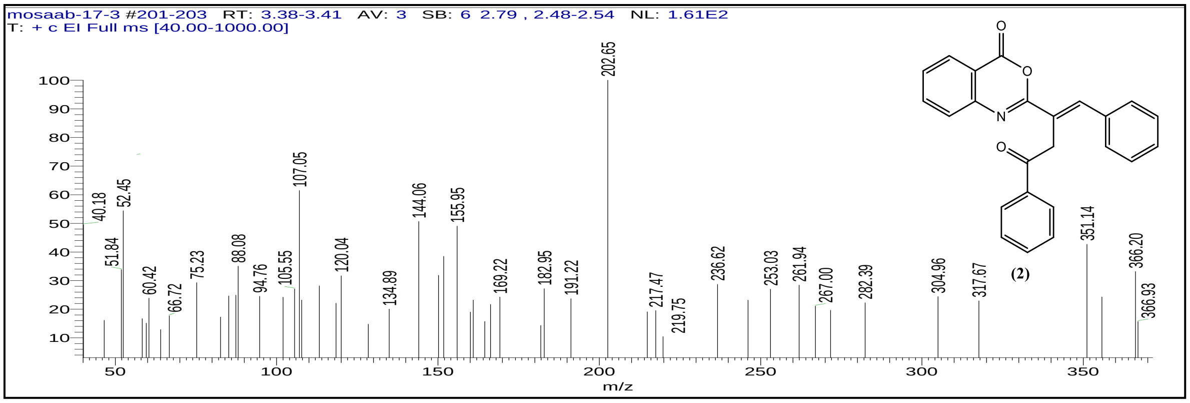

Mass spectrum of compound **2**

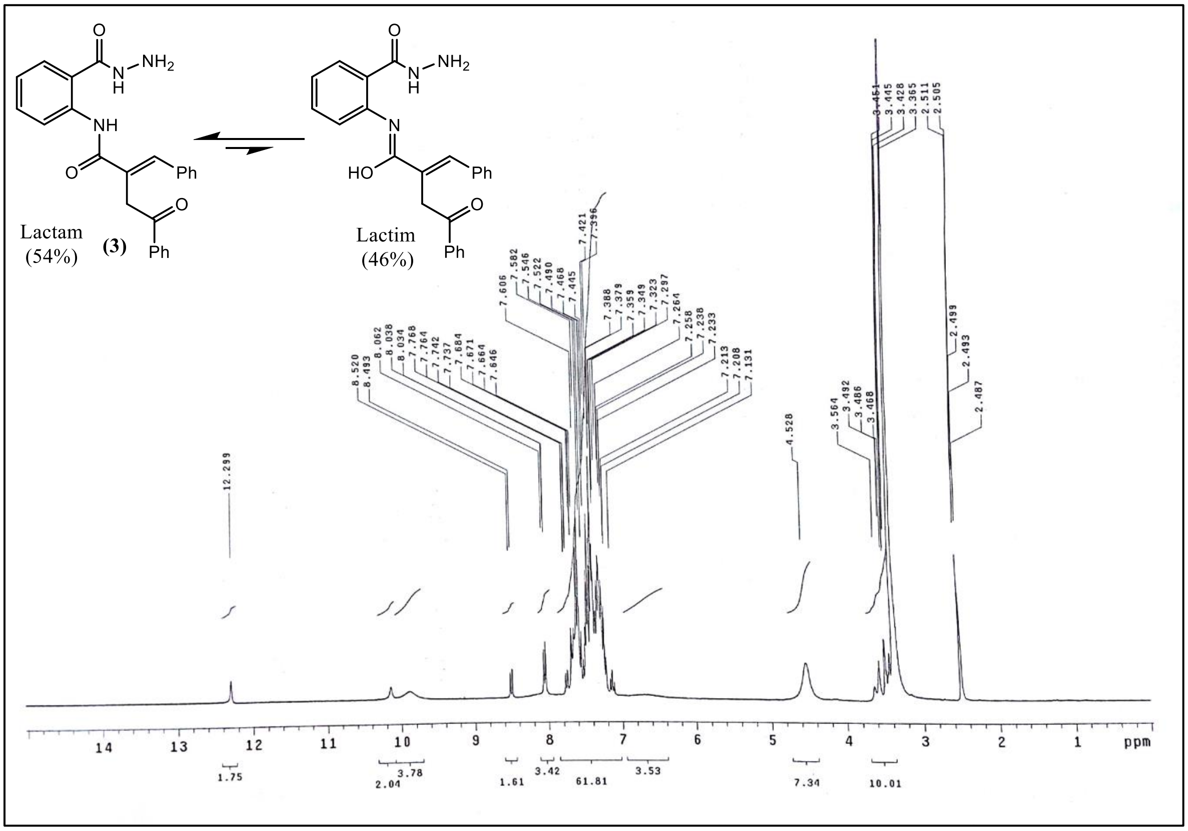

^{1}H-NMR (DMSO-*d6*) spectrum compound of **3**

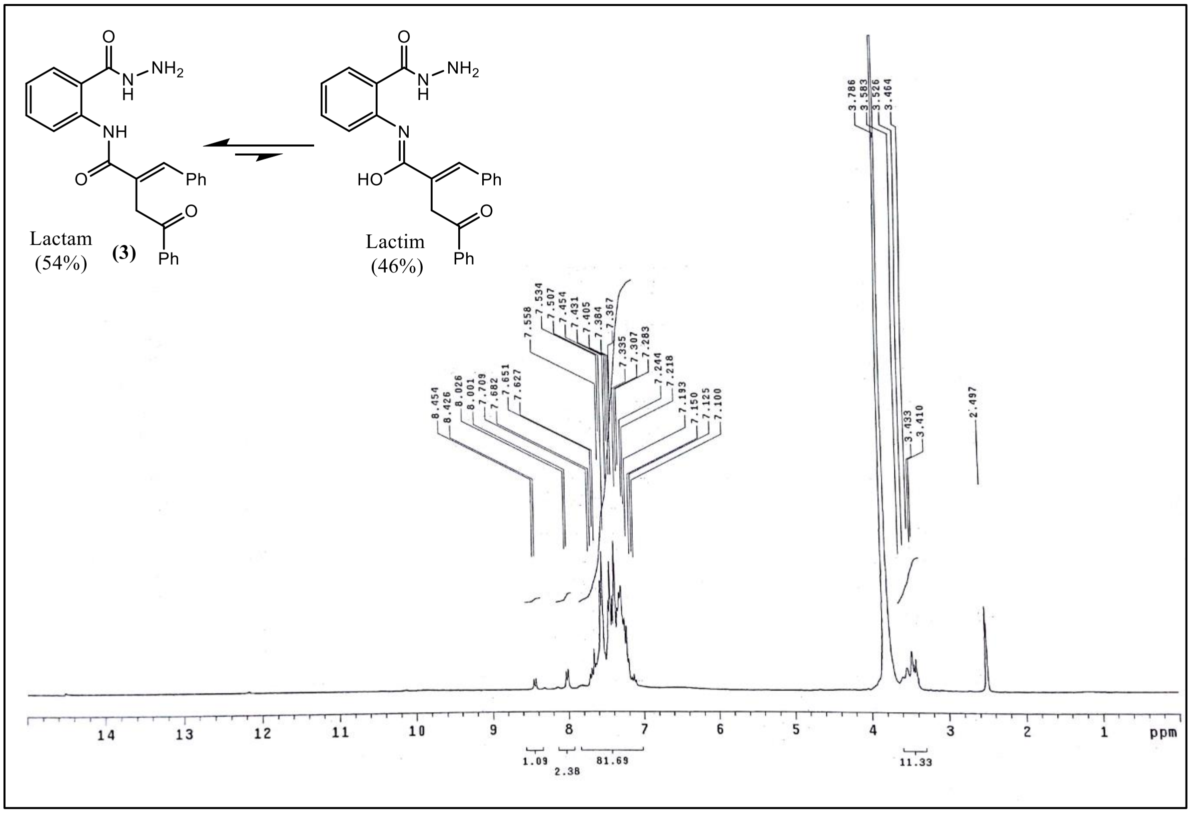

^{1}H-NMR (DMSO-*d6*) + D_2O spectrum compound of **3**

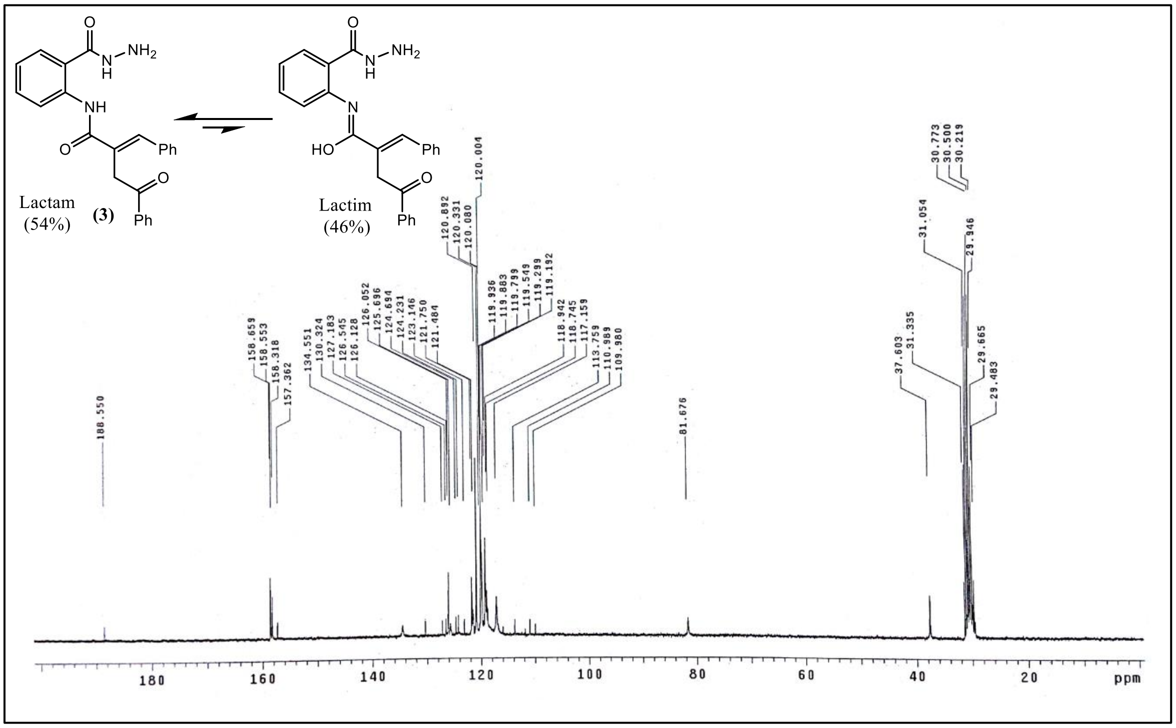

^{13}C-NMR (DMSO-d^6) spectrum compound of **3**

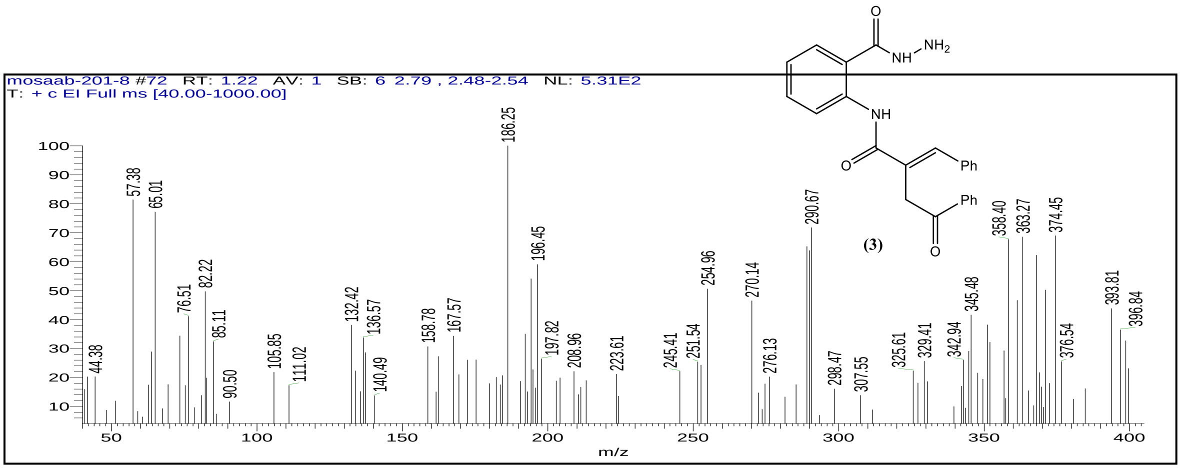

Mass spectrum of compound **3**

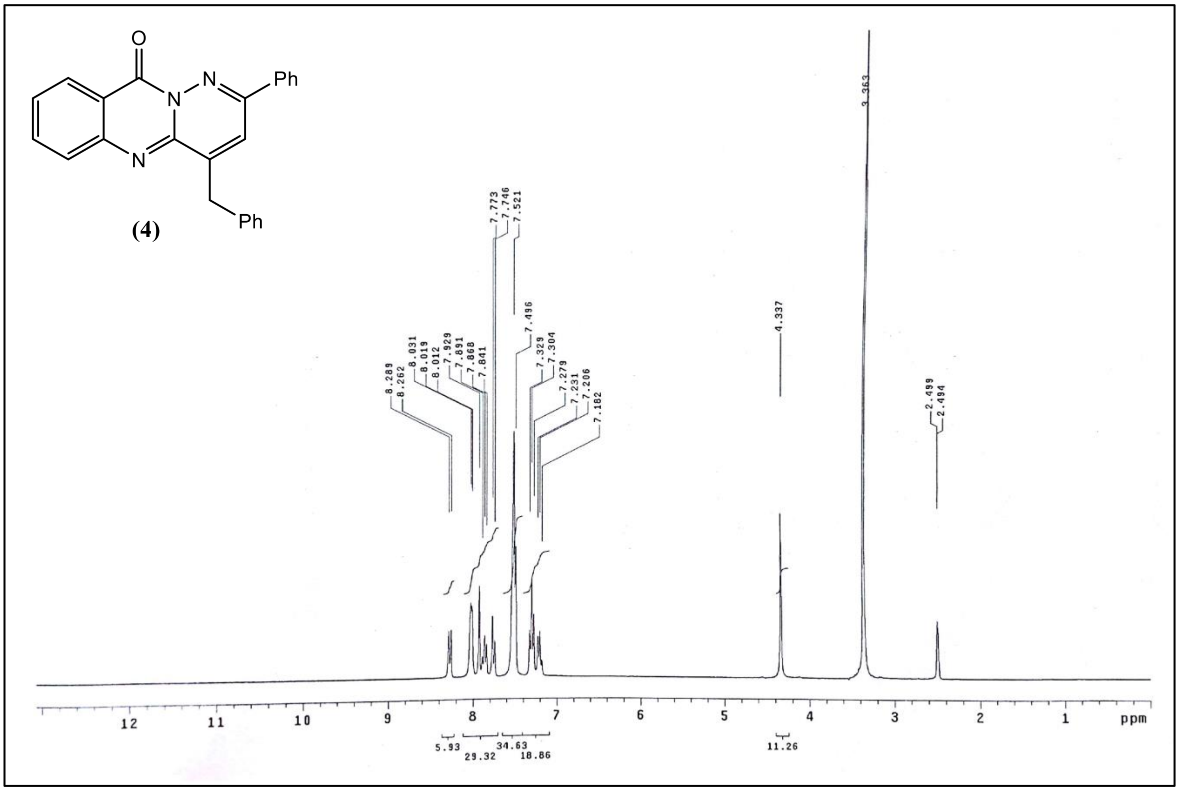

^{1}H-NMR (DMSO-*d6*) spectrum compound of **4**

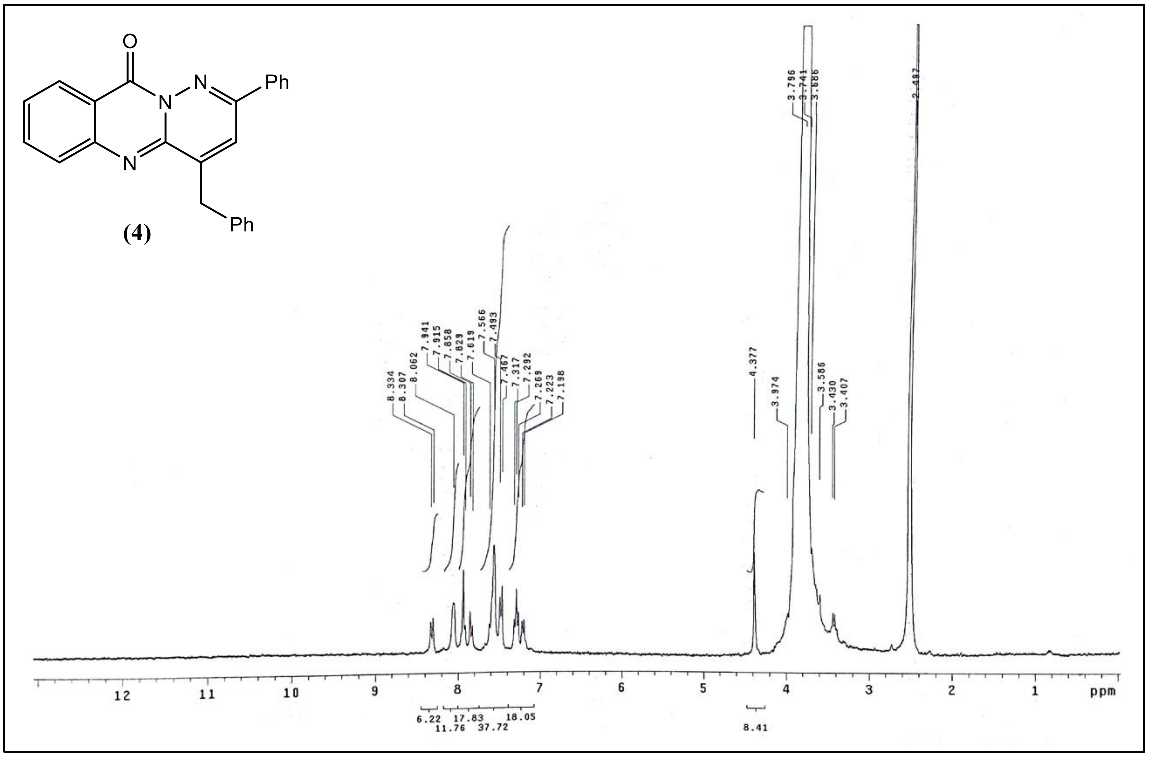

^{1}H-NMR (DMSO-*d6*) + D_2O spectrum compound of **4**

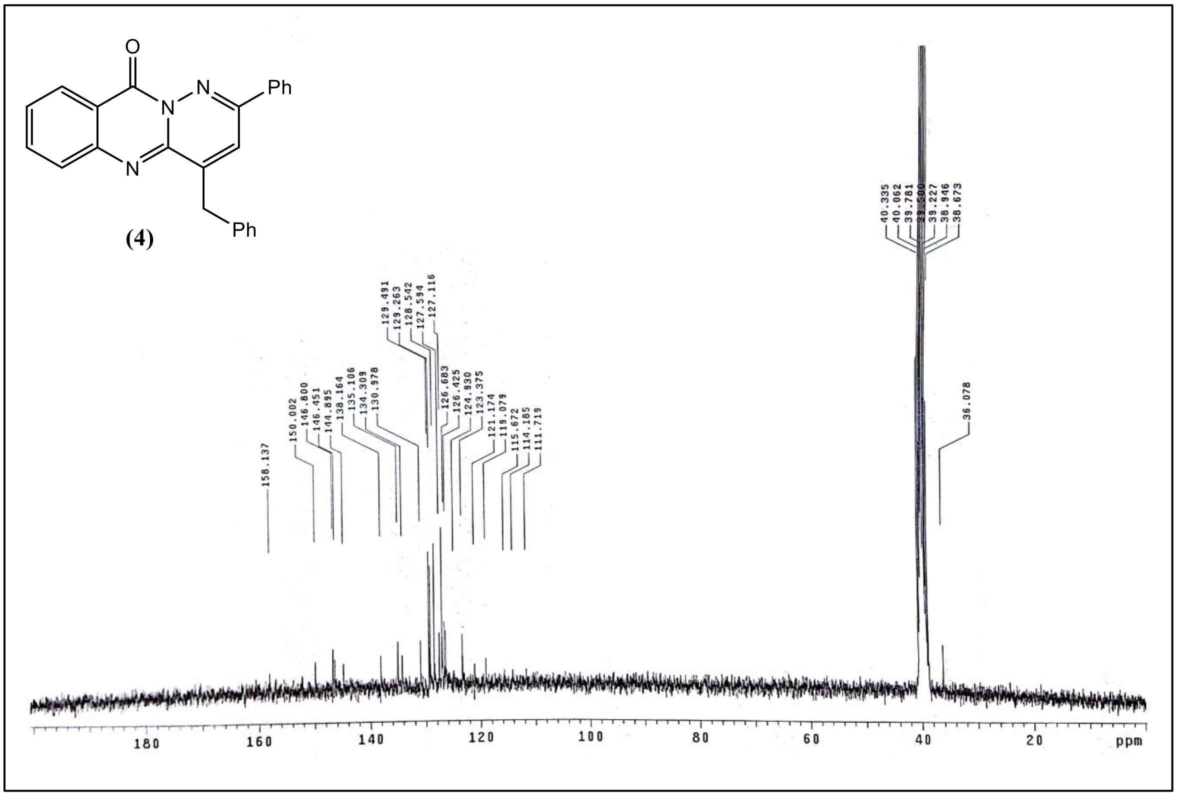

^{13}C-NMR (DMSO-*d*[6]) spectrum compound of **4**

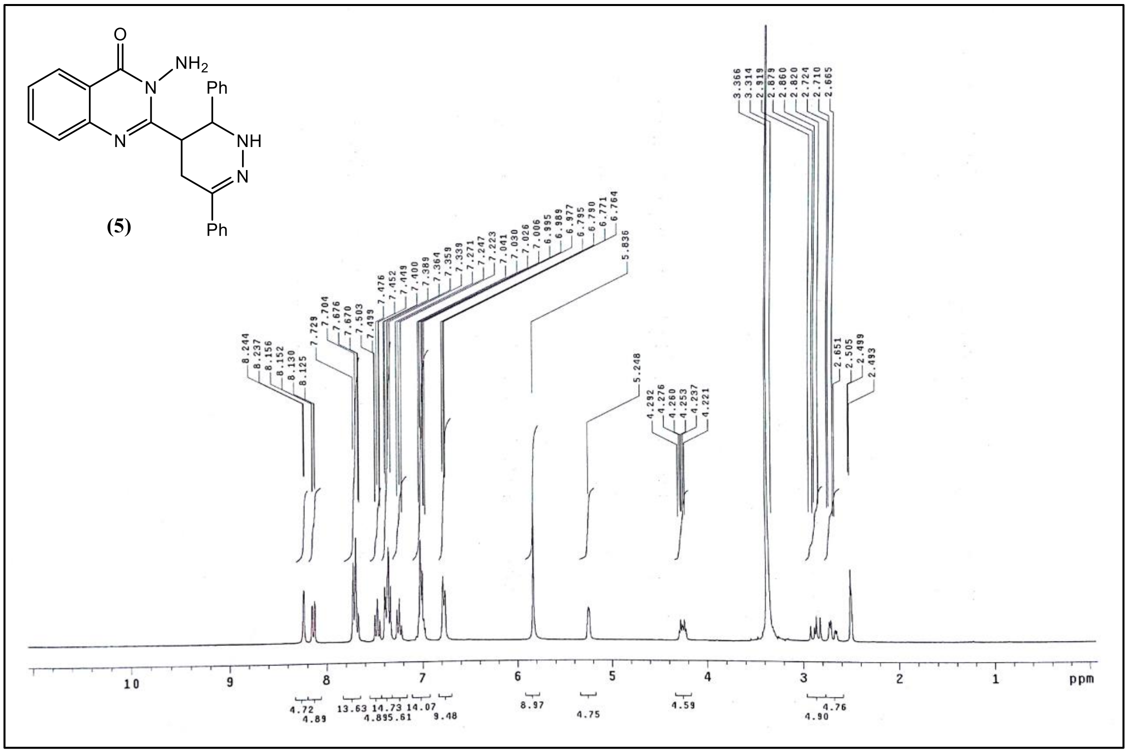

^{1}H-NMR (DMSO-*d6*) spectrum compound of **5**

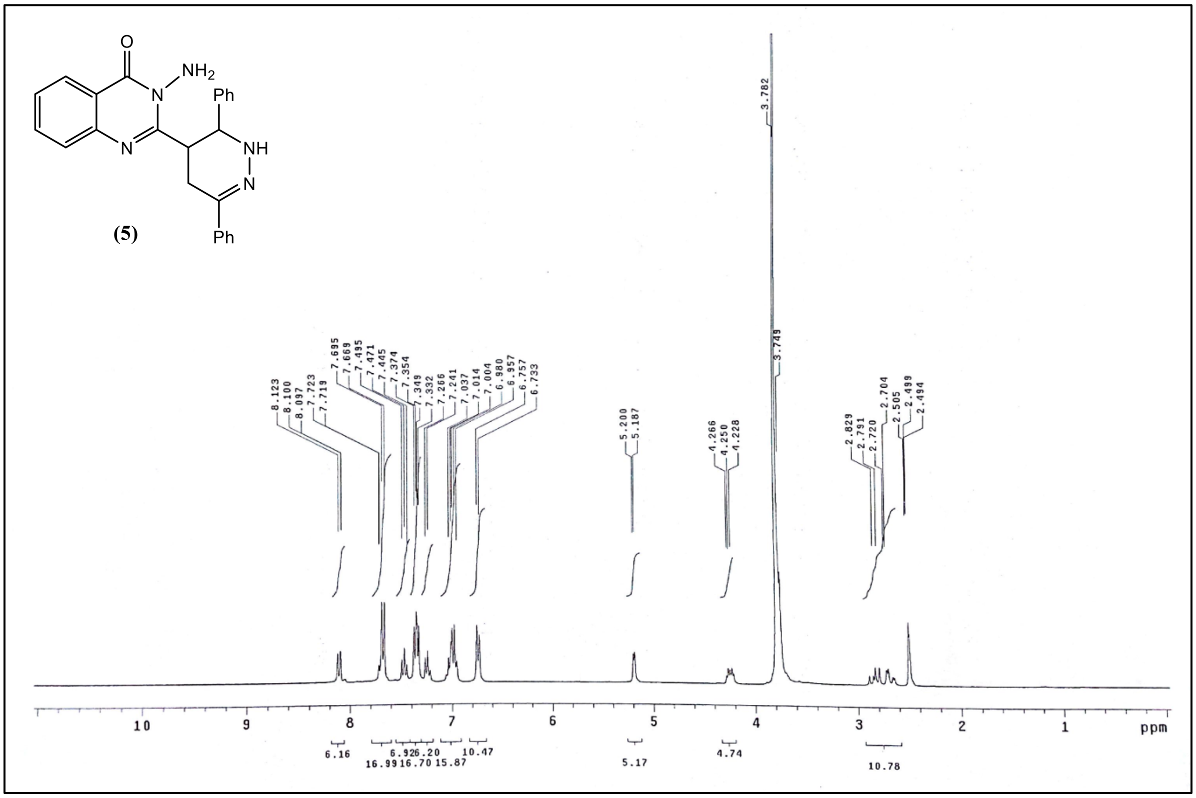

^{1}H-NMR (DMSO-*d6*) + D_2O spectrum compound of **5**

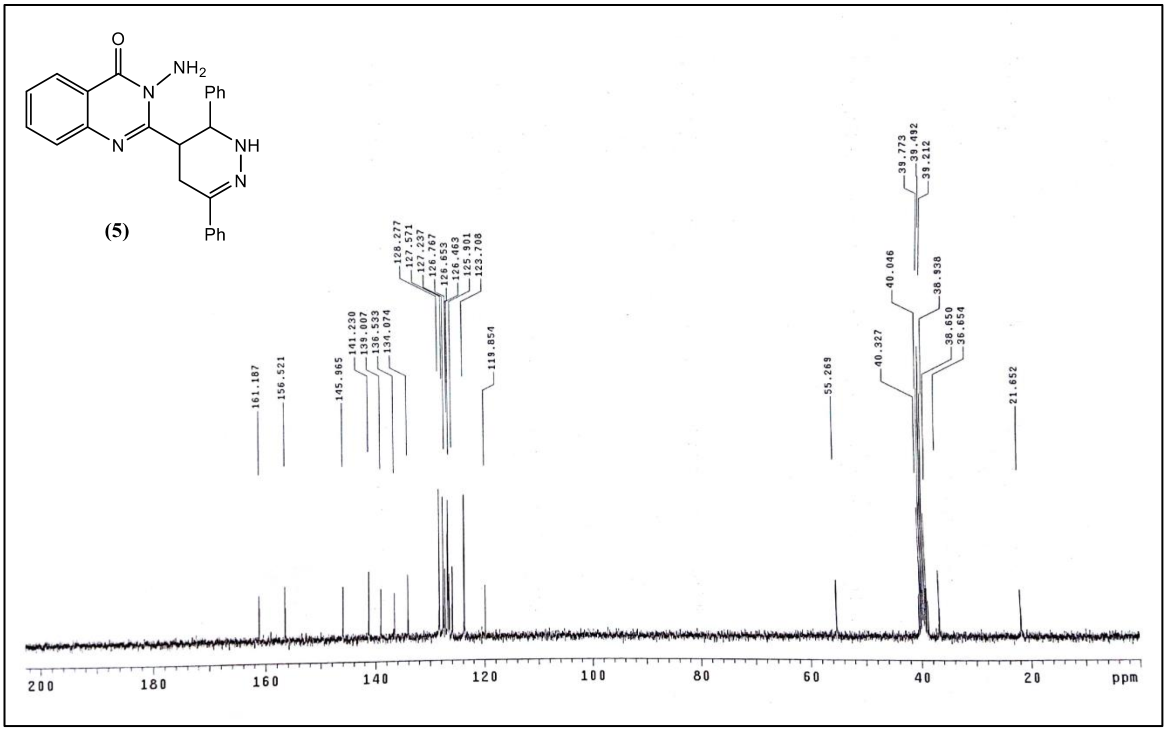

^{13}C-NMR (DMSO-d^6) spectrum compound of **5**

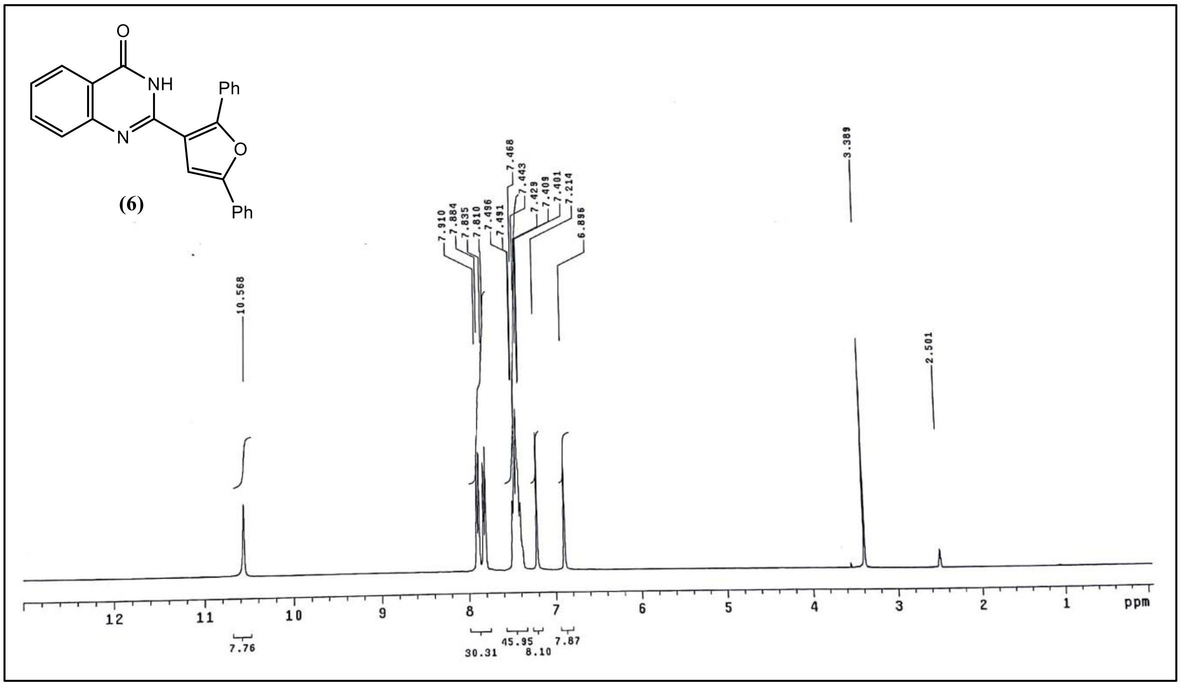

^{1}H-NMR (DMSO-*d6*) spectrum compound of **6**

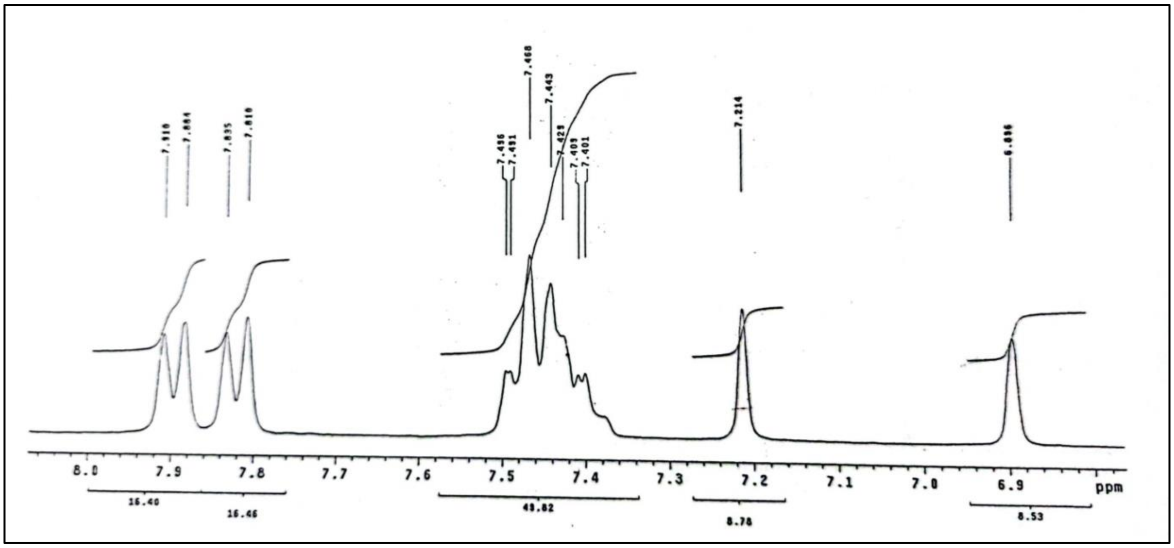

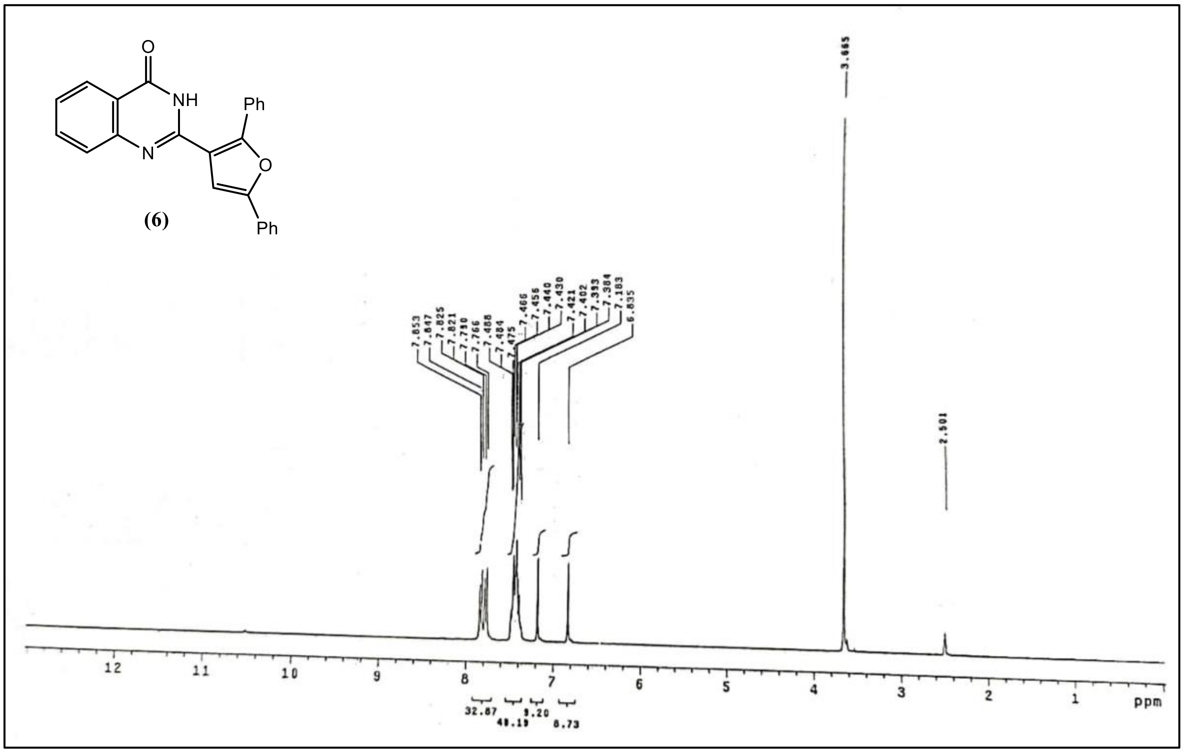

^{1}H-NMR (DMSO-*d6*) + D_2O spectrum compound of **6**

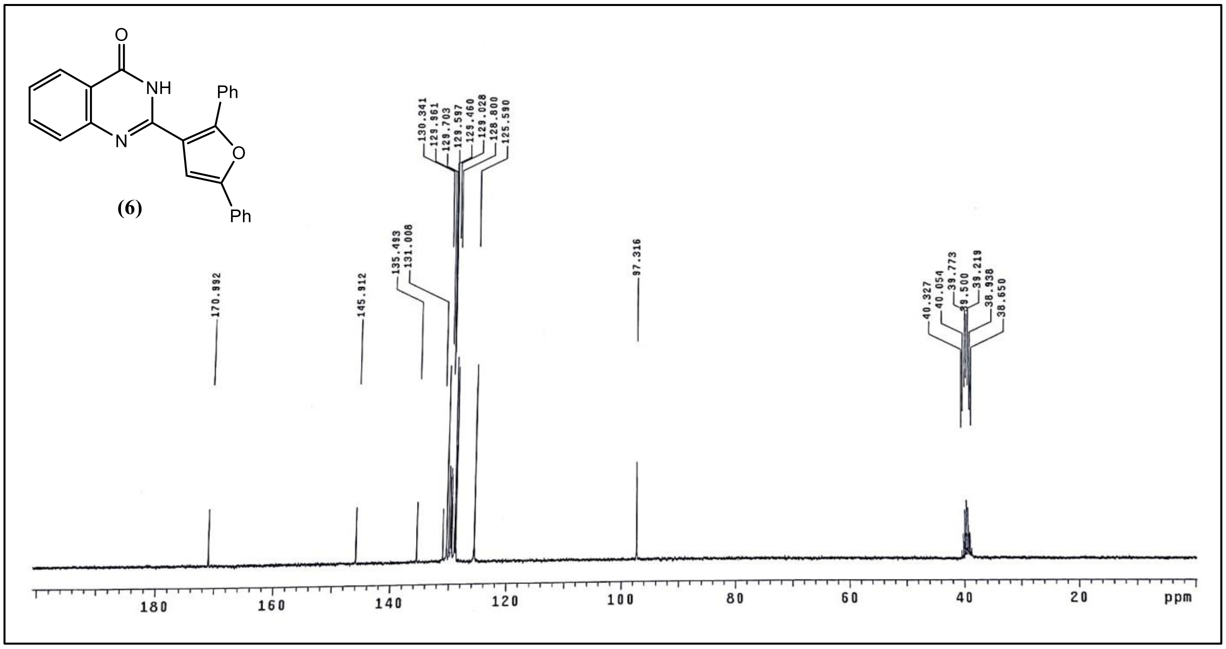

^{13}C-NMR (DMSO-d^{6}) spectrum compound of **6**

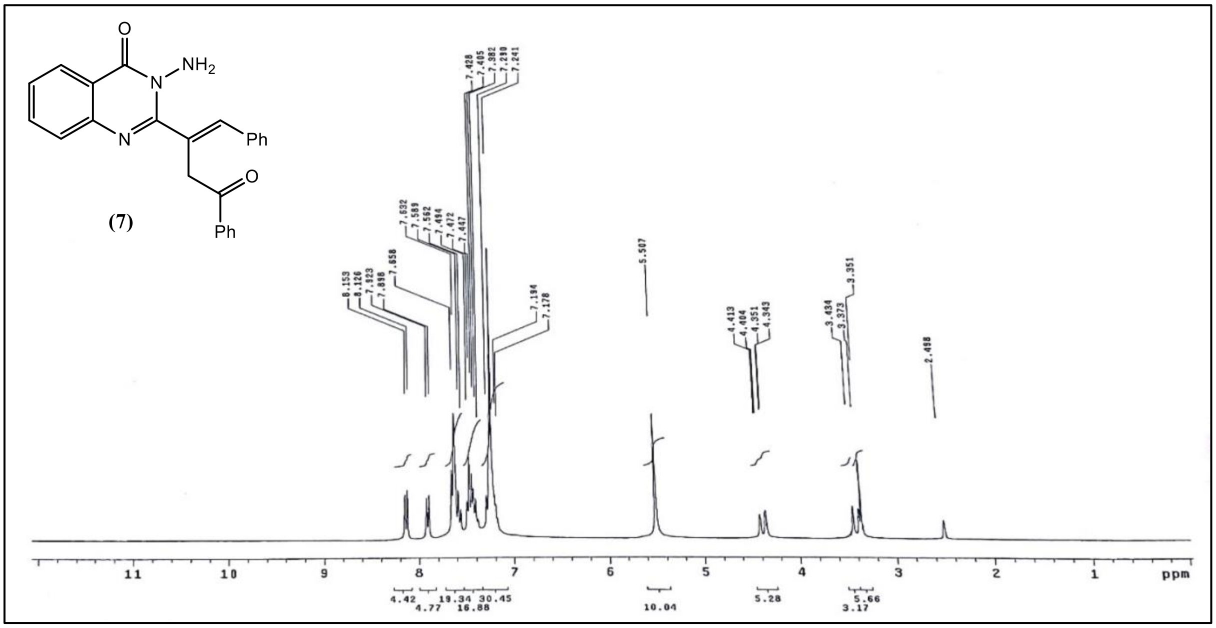

^{1}H-NMR (DMSO-*d6*) spectrum compound of **7**

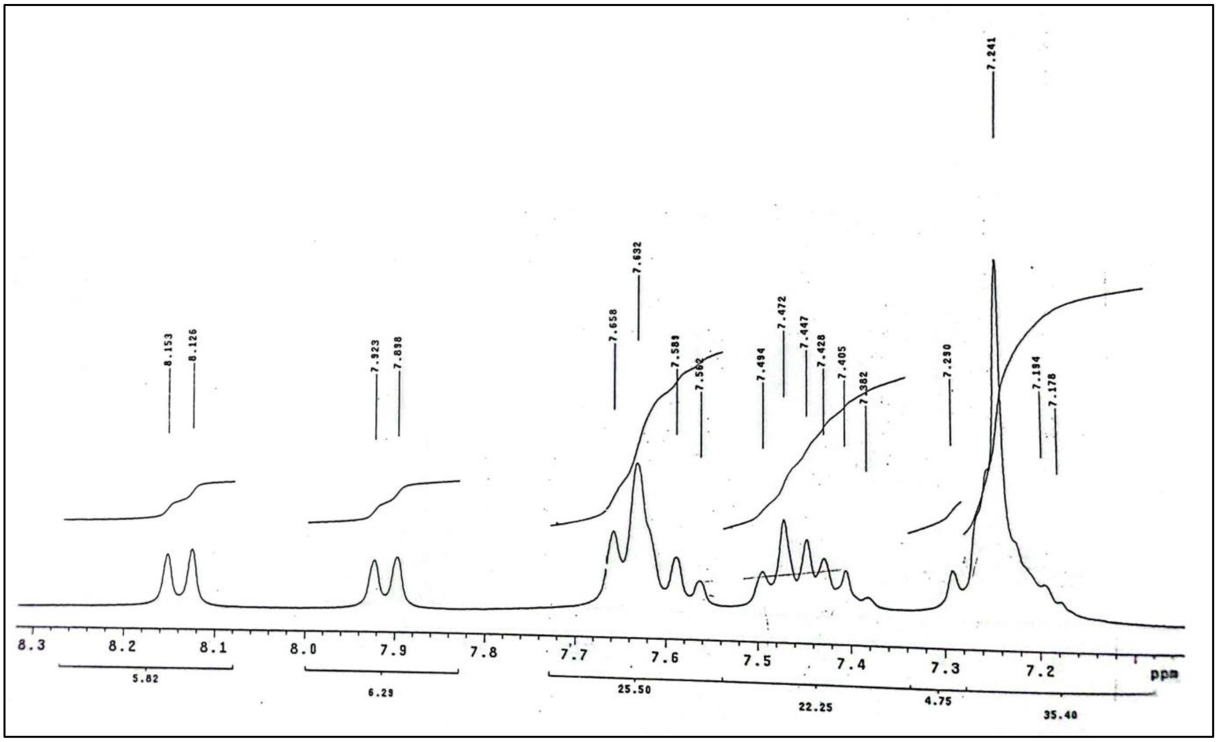

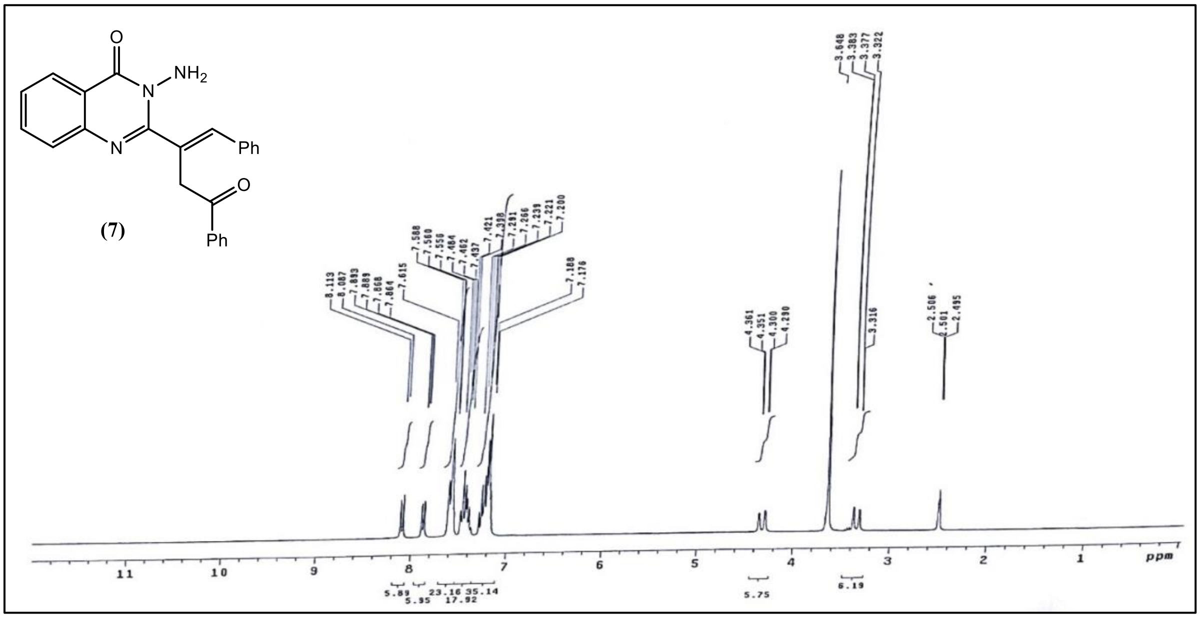

^{1}H-NMR (DMSO-*d6*) + D_2O spectrum compound of **7**

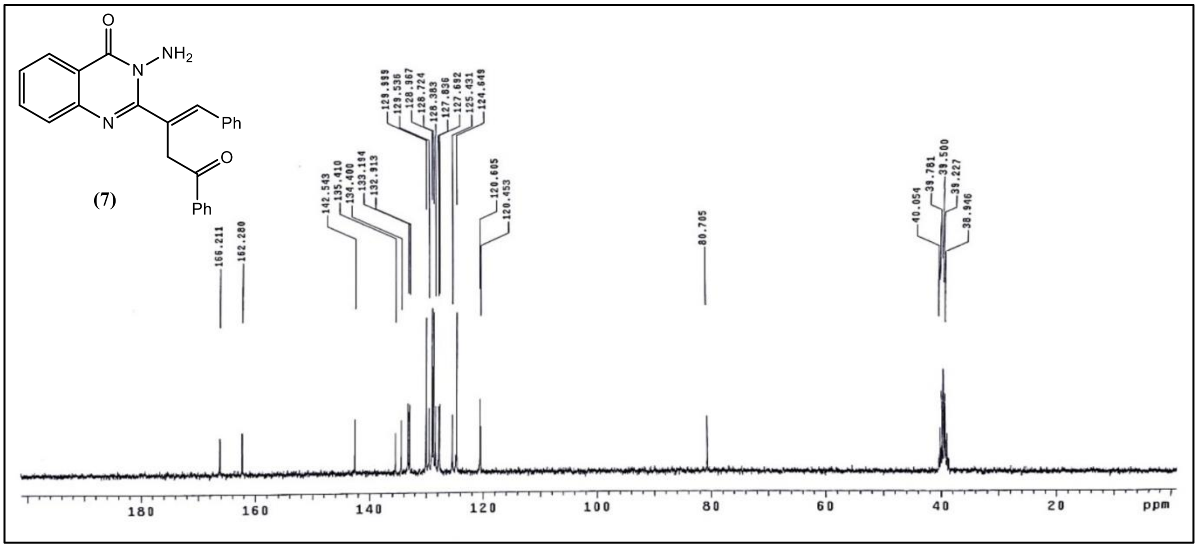

^{13}C-NMR (DMSO-d^6) spectrum compound of **7**

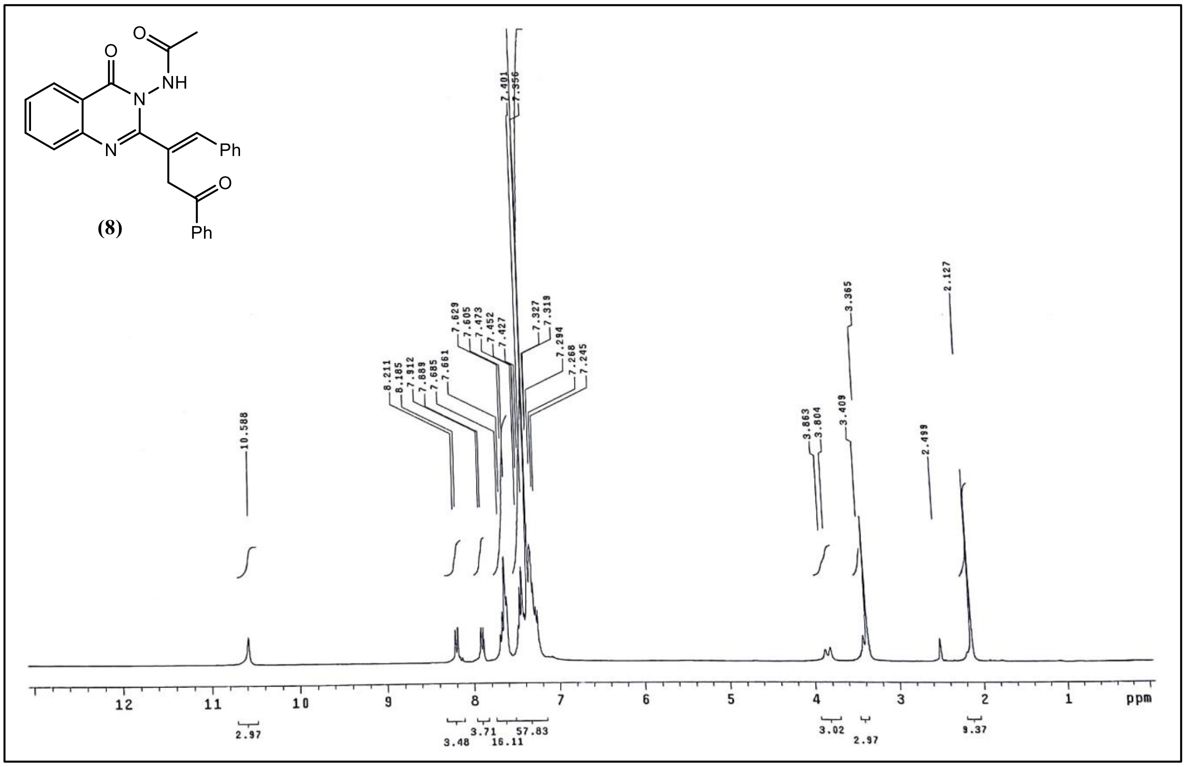

^{1}H-NMR (DMSO-$d6$) spectrum compound of **8**

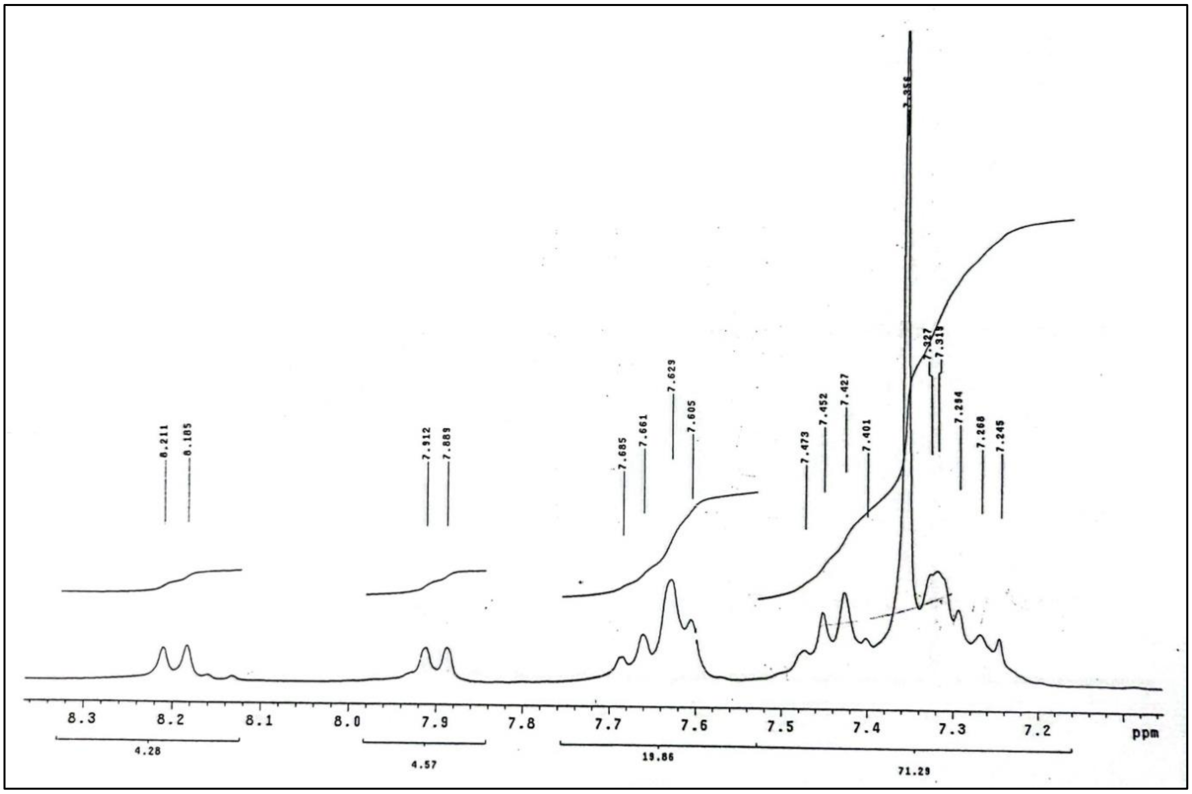

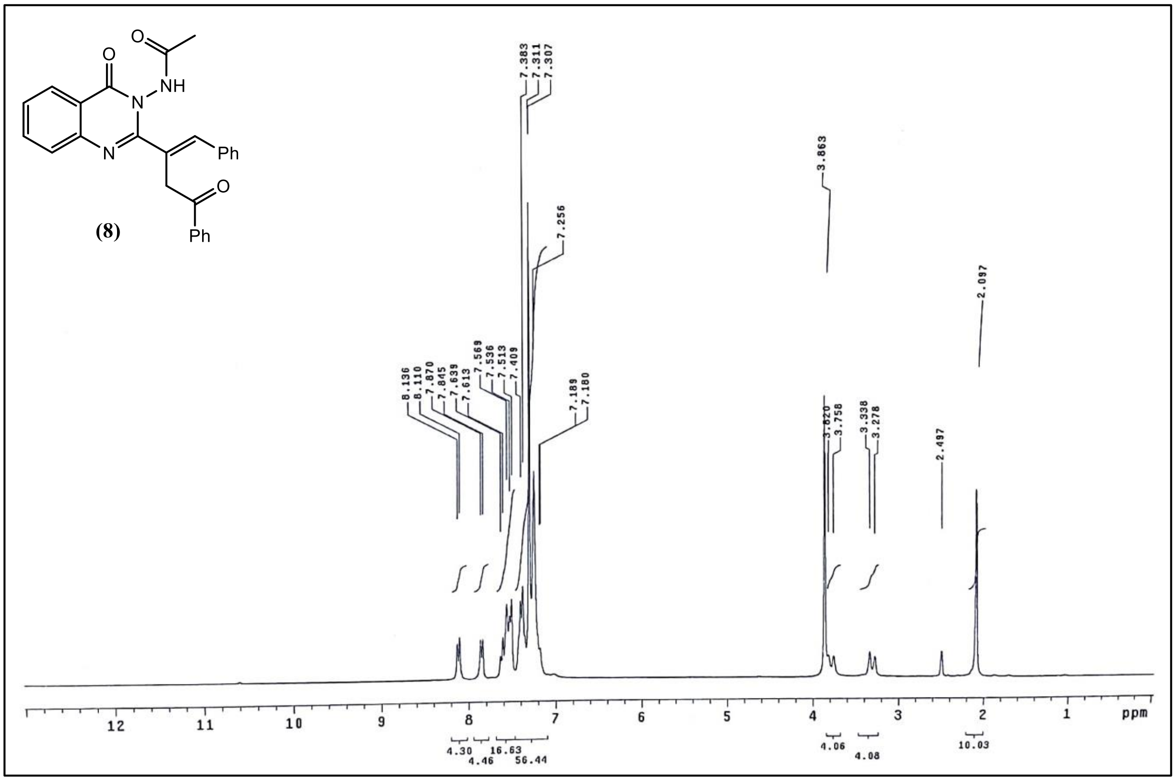

(8)

^{1}H-NMR (DMSO-*d6*) + D_2O spectrum compound of **8**

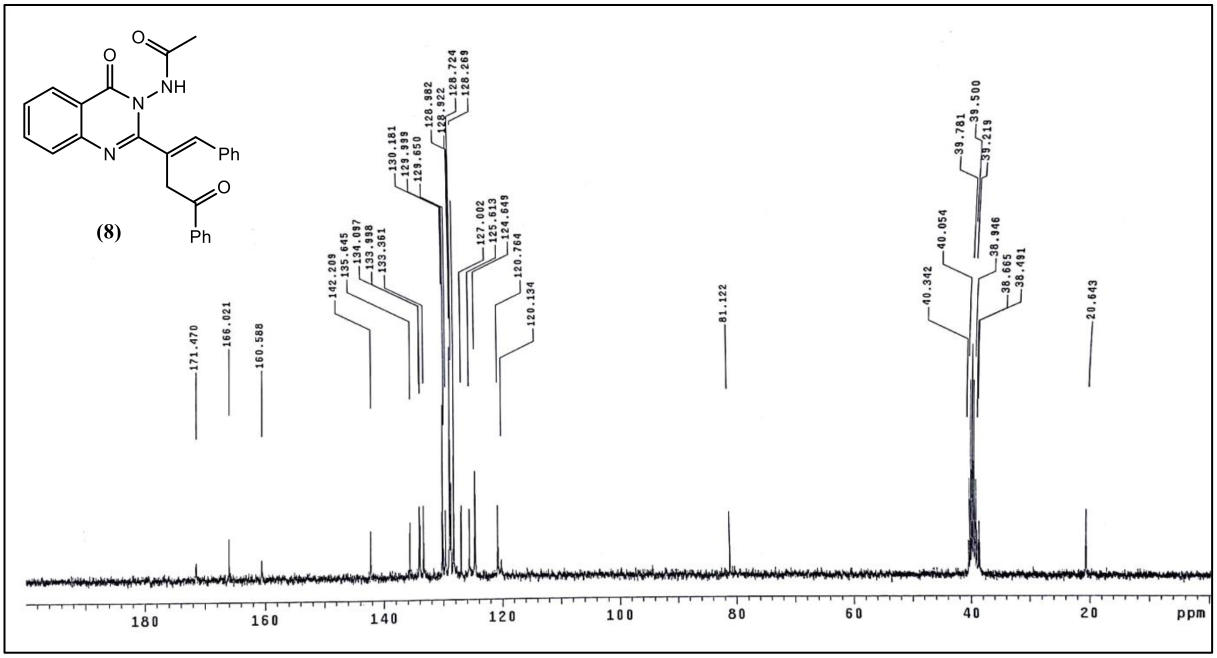

^{13}C-NMR (DMSO-*d*^{6}) spectrum compound of **8**

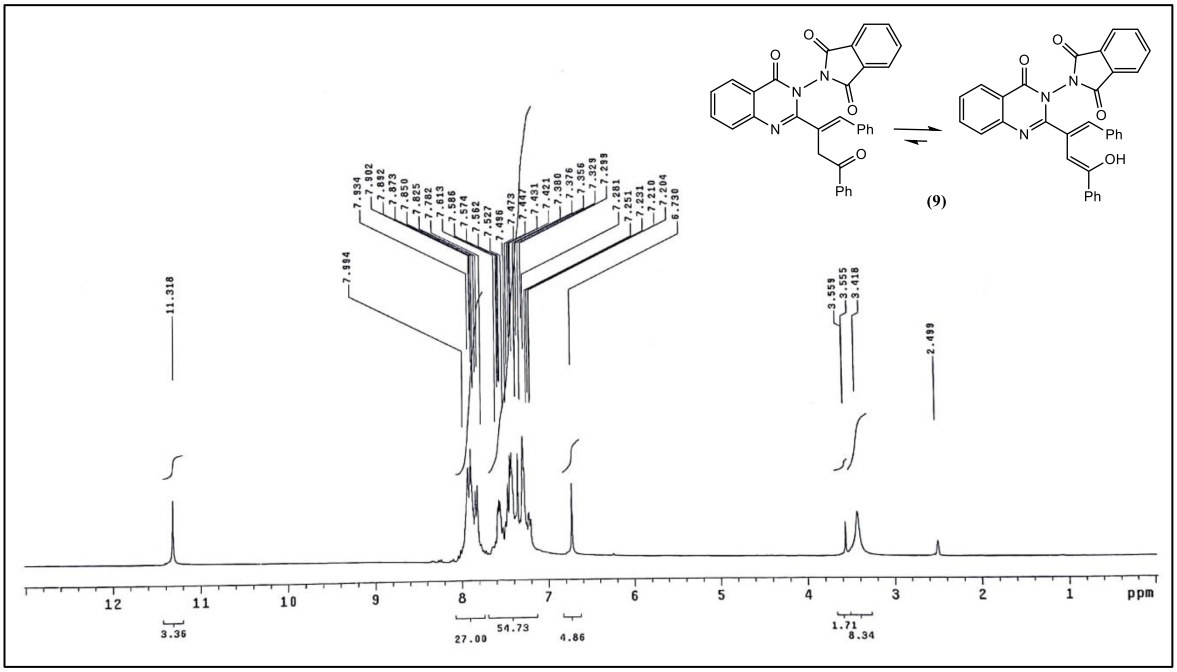

^{1}H-NMR (DMSO-*d6*) spectrum compound of **9**

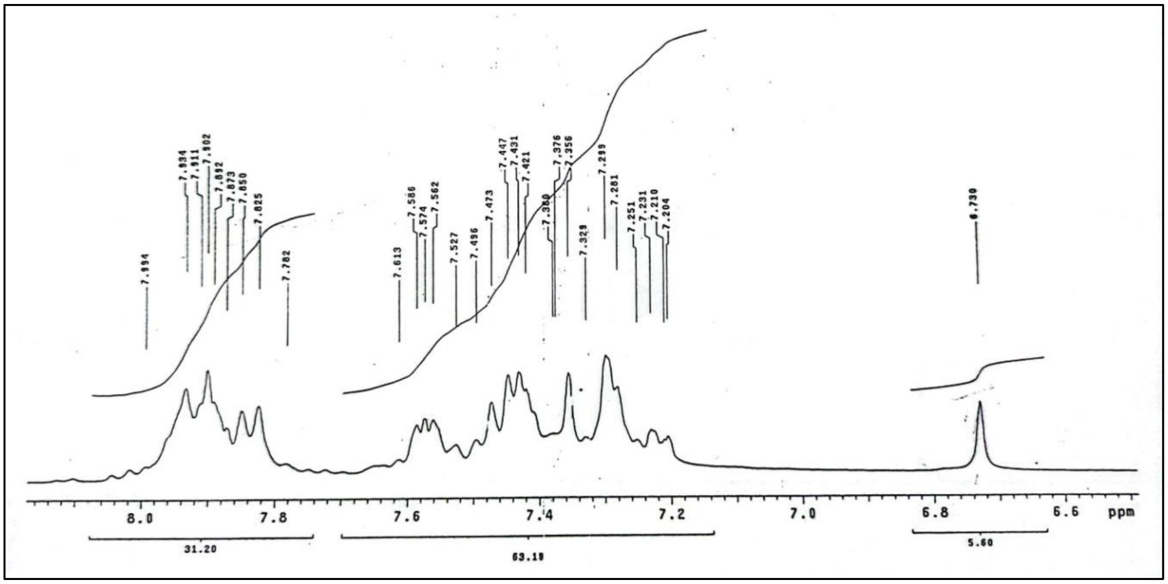

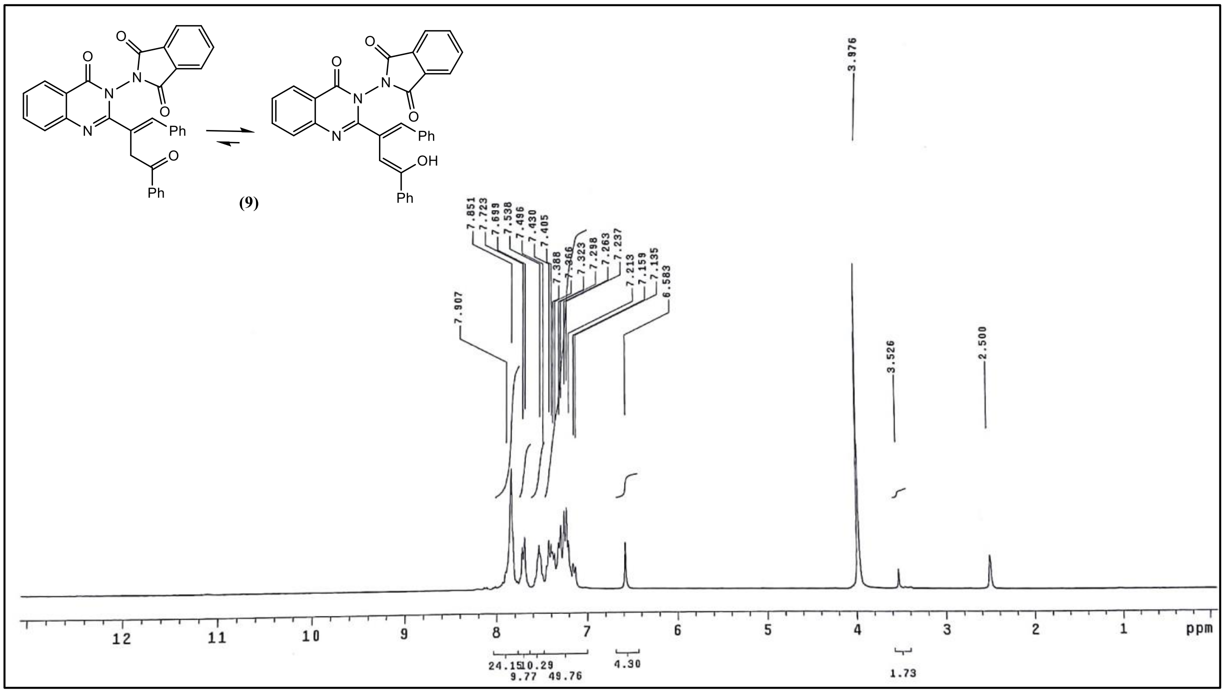

^{1}H-NMR (DMSO-*d6*) + D_2O spectrum compound of **9**

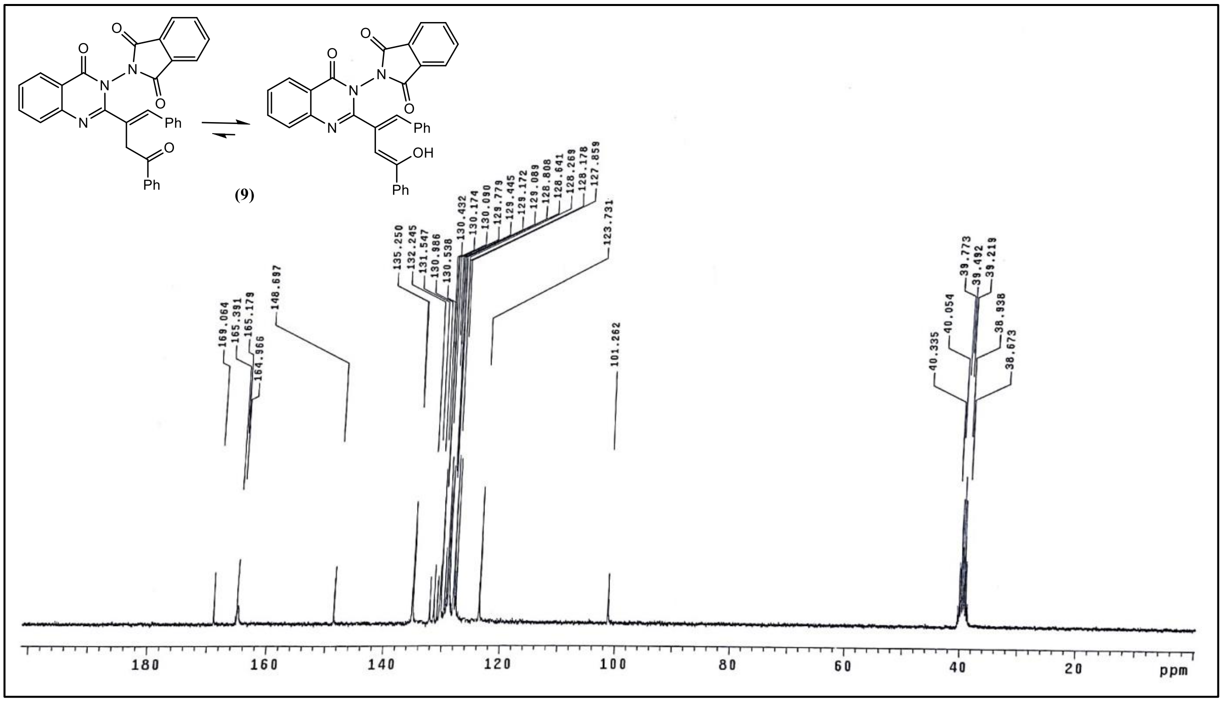

^{13}C-NMR (DMSO-d^6) spectrum compound of **9**

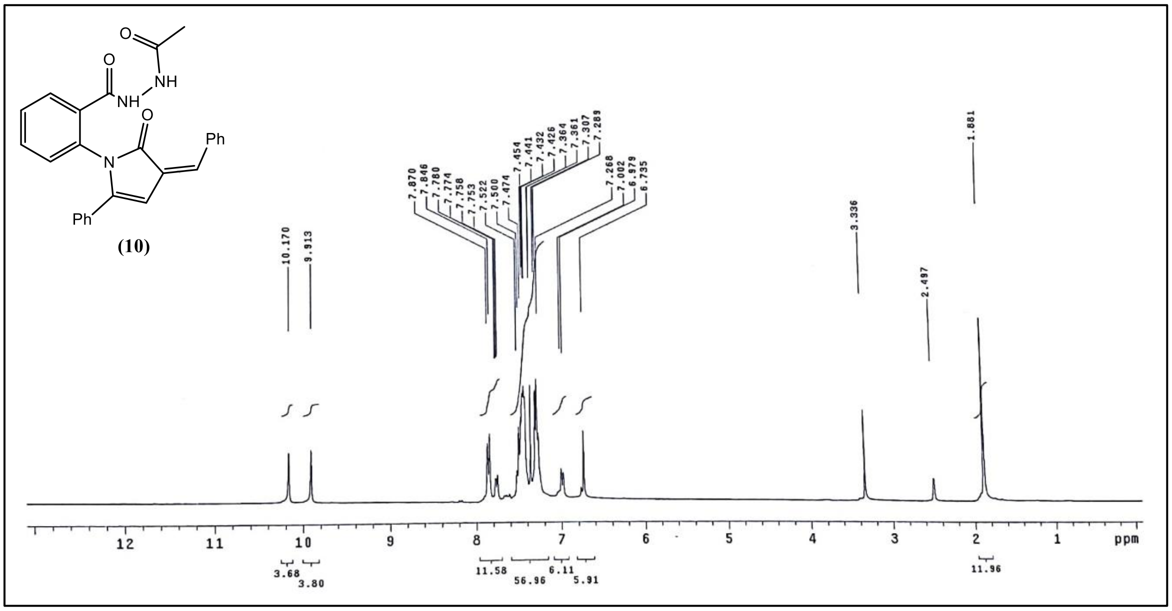

^{1}H-NMR (DMSO-*d6*) spectrum compound of **10**

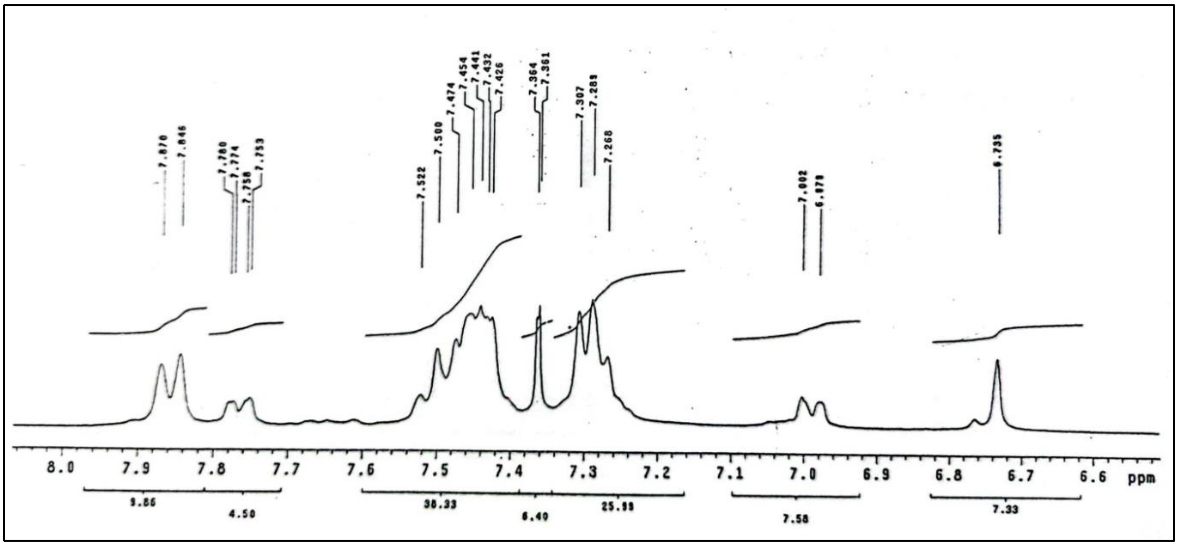

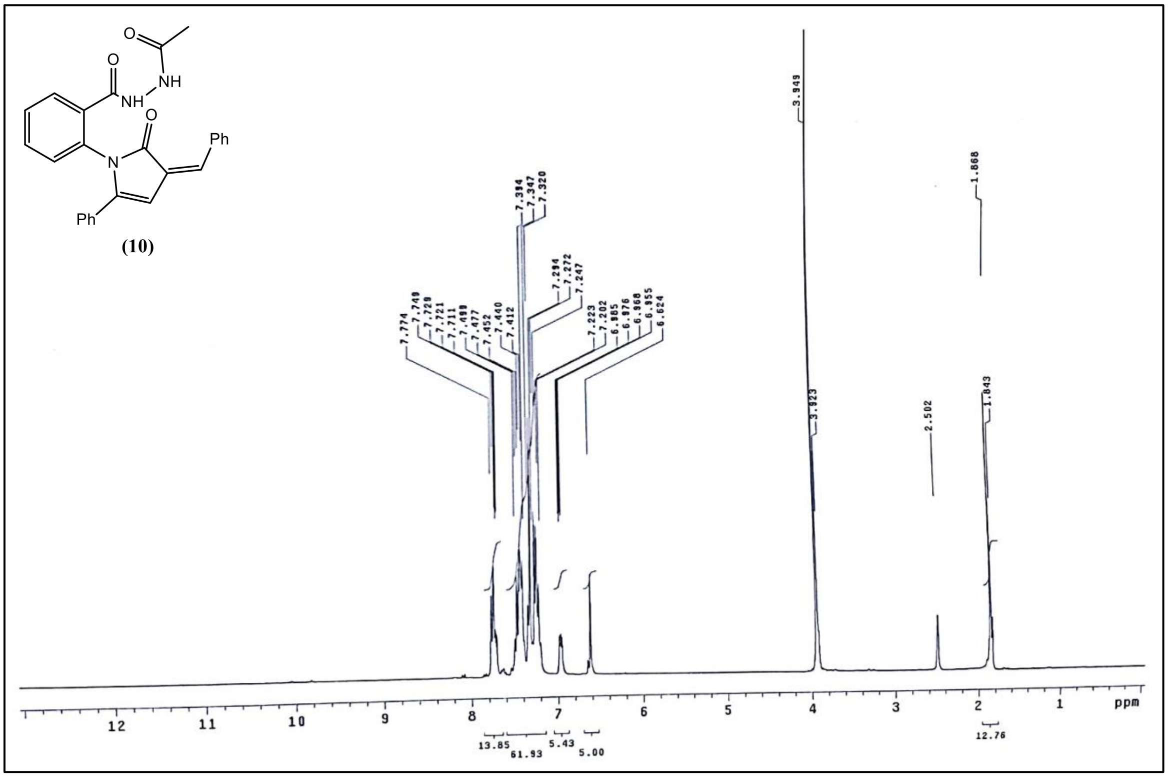

^{1}H-NMR (DMSO-*d6*) + D_2O spectrum compound of **10**

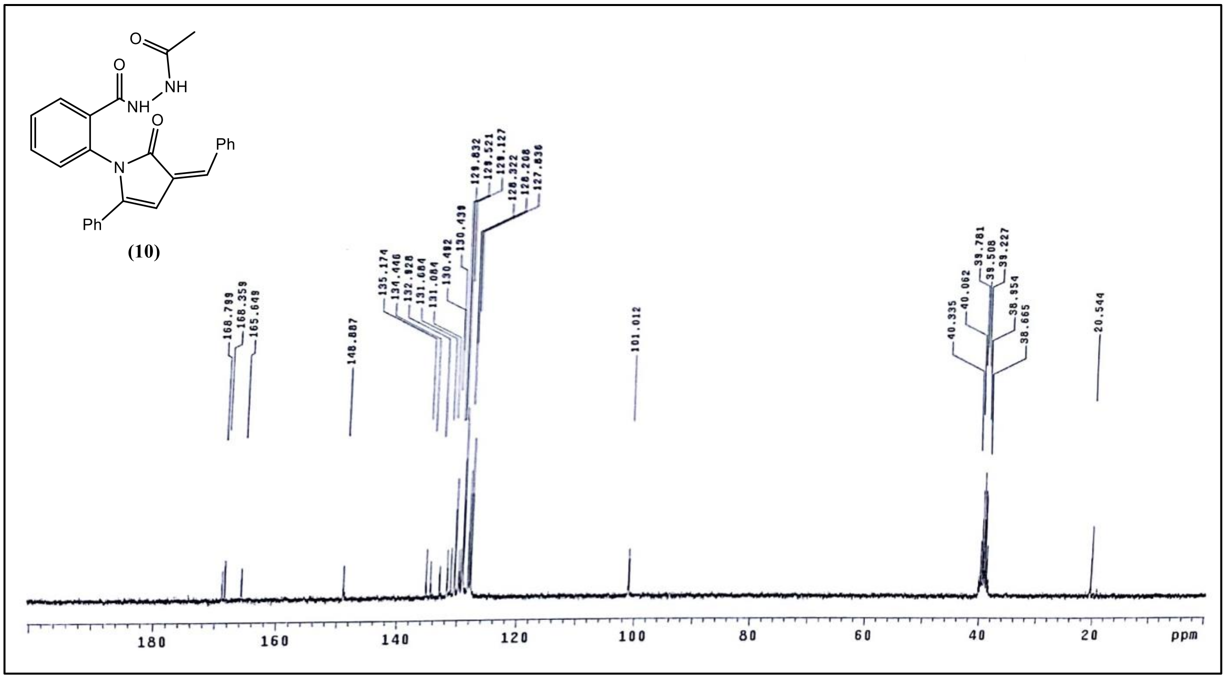

^{13}C-NMR (DMSO-*d*^{6}) spectrum compound of **10**

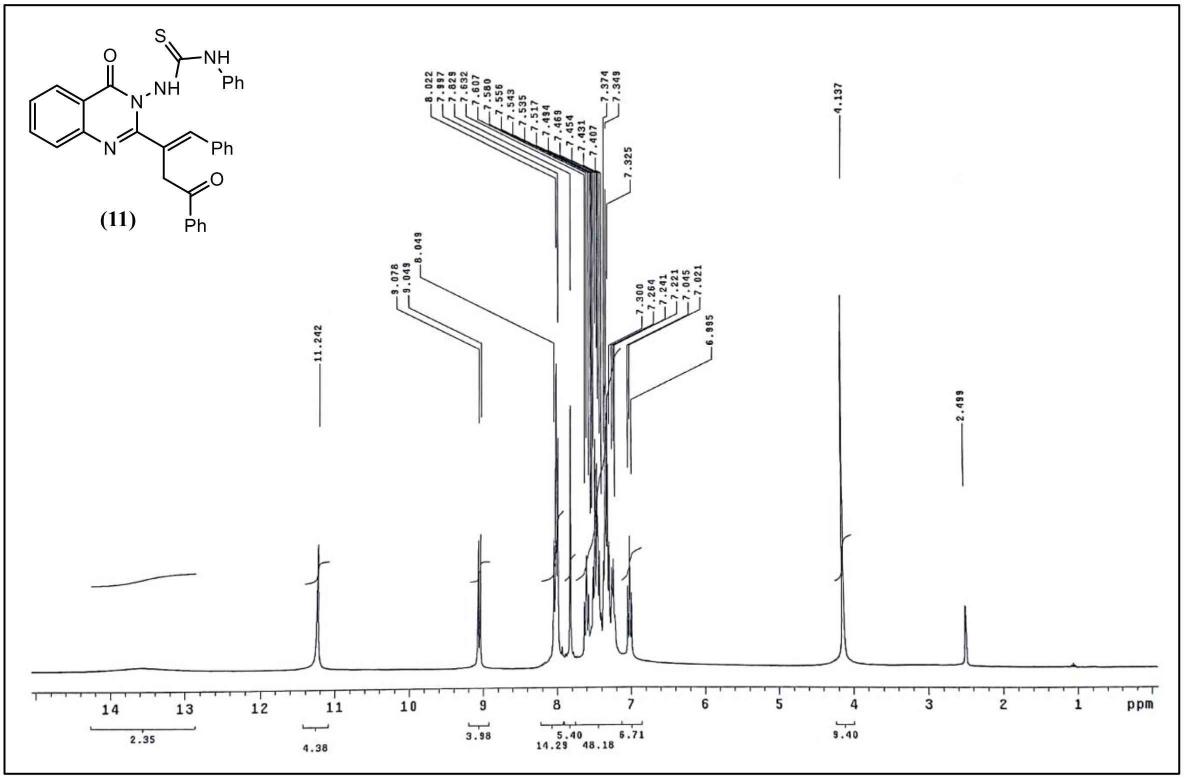

^{1}H-NMR (DMSO-*d6*) spectrum compound of **11**

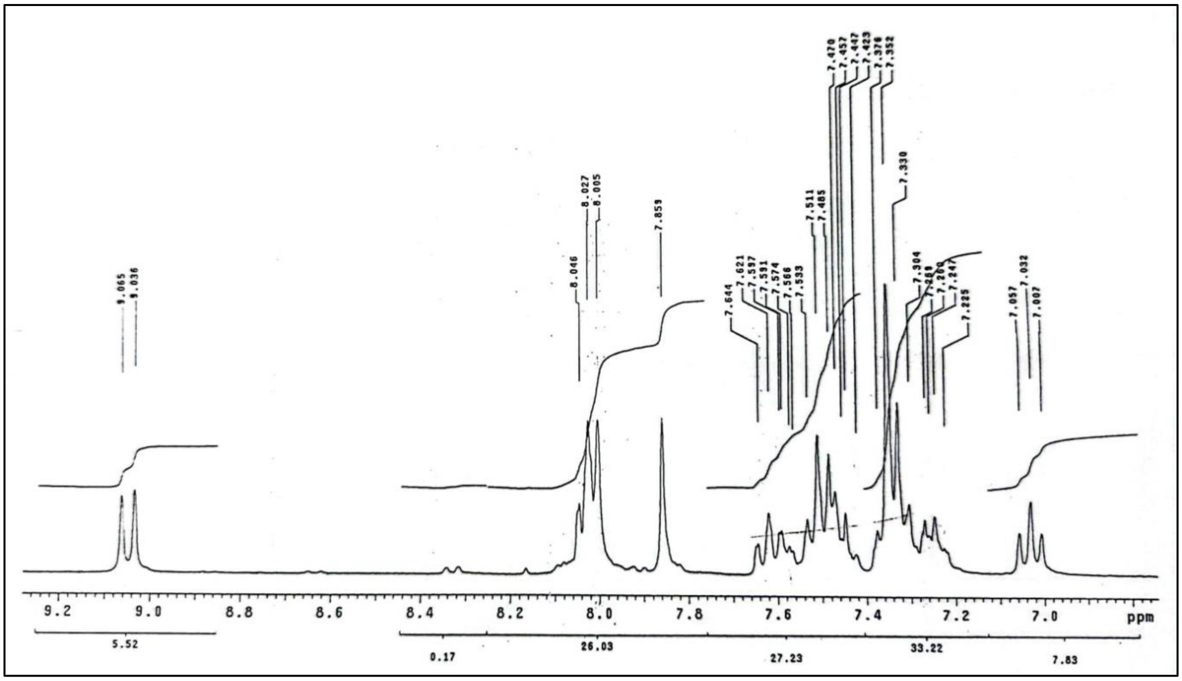

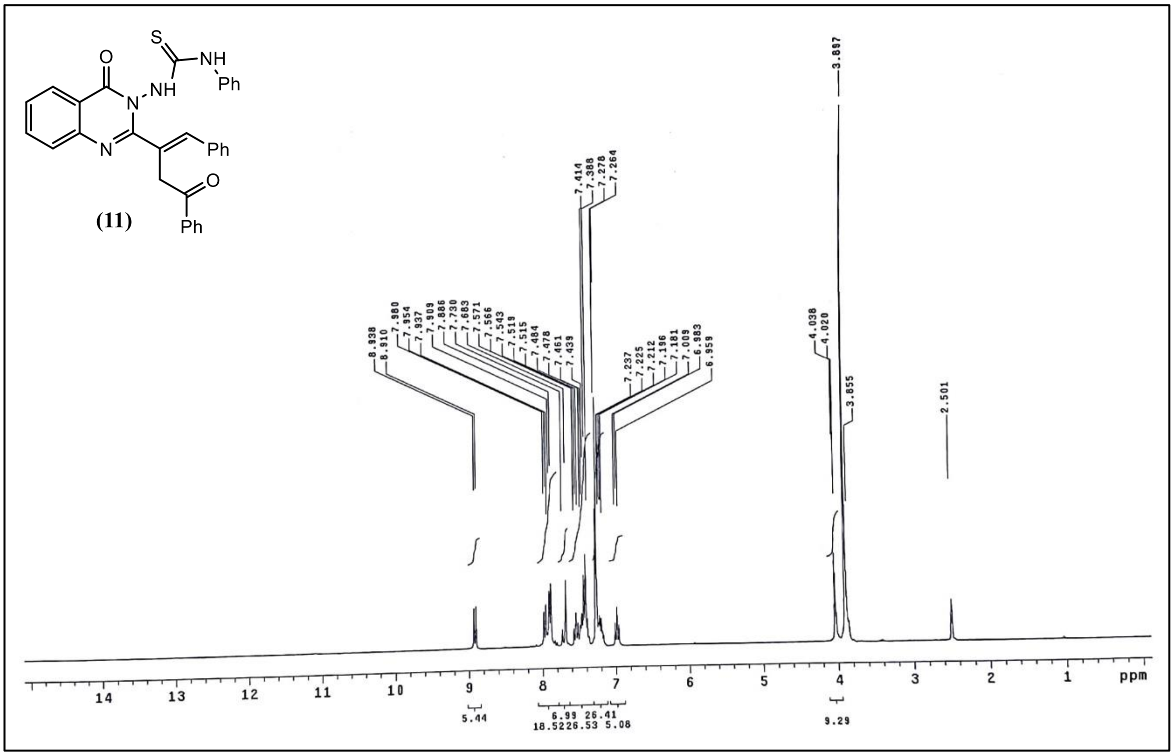

^{1}H-NMR (DMSO-*d6*) + D_2O spectrum compound of **11**

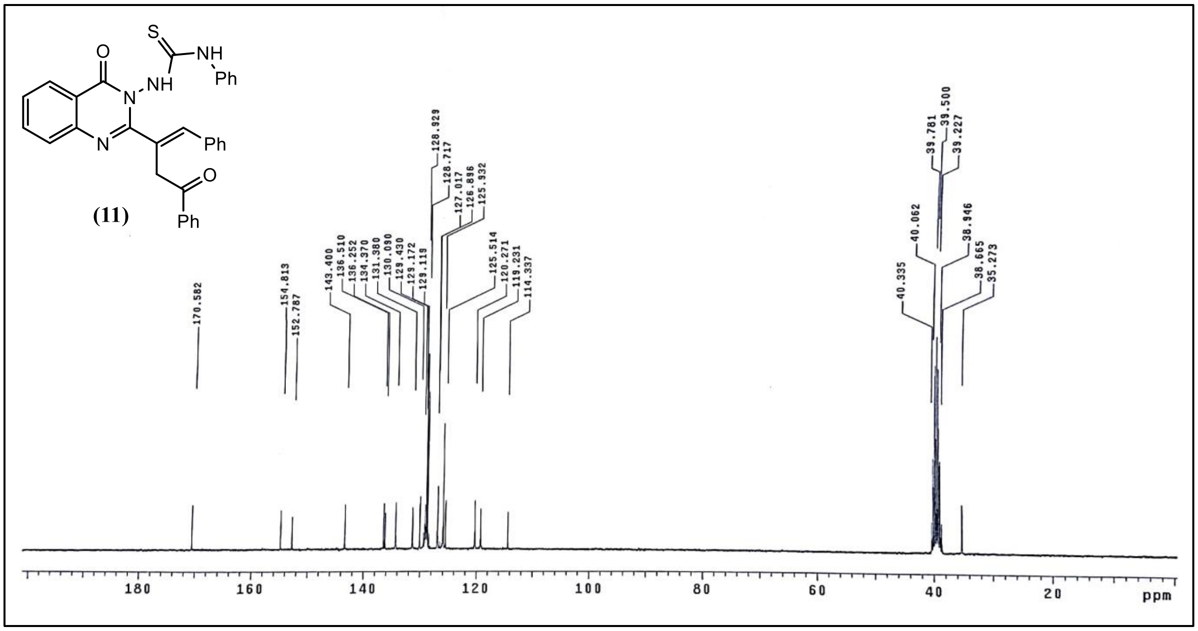

^{13}C-NMR (DMSO-d^6) spectrum compound of **11**

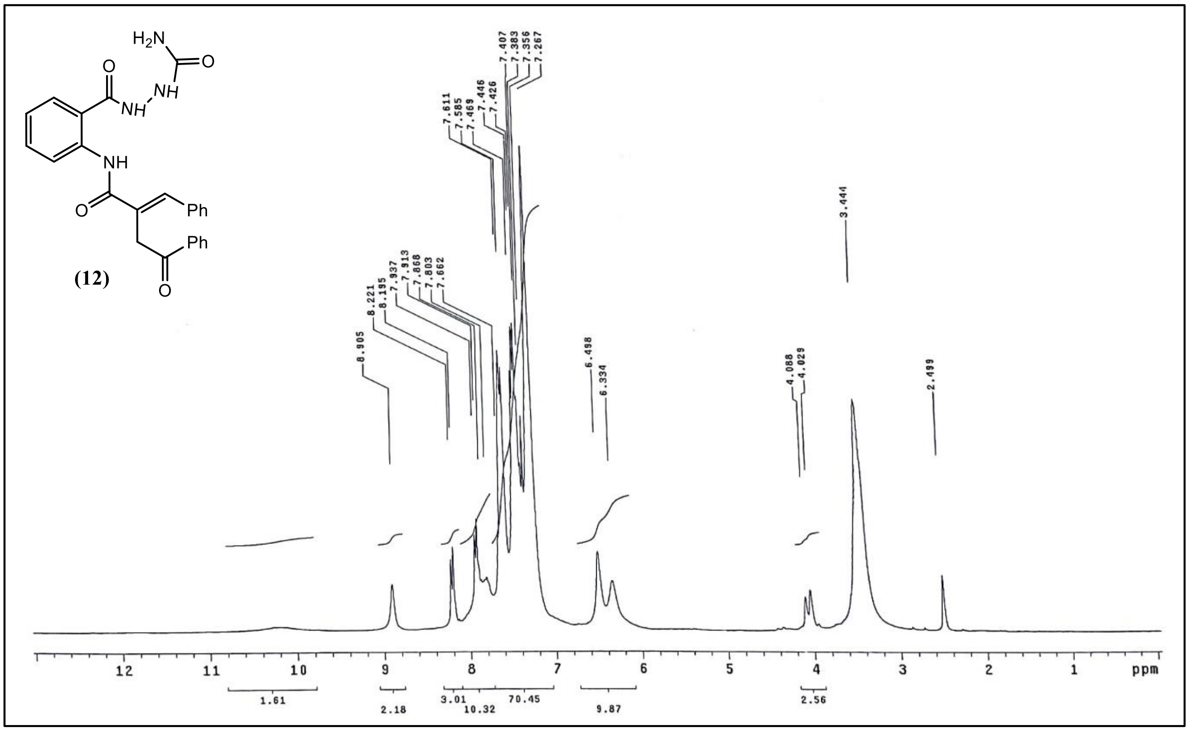

^{1}H-NMR (DMSO-*d6*) spectrum compound of **12**

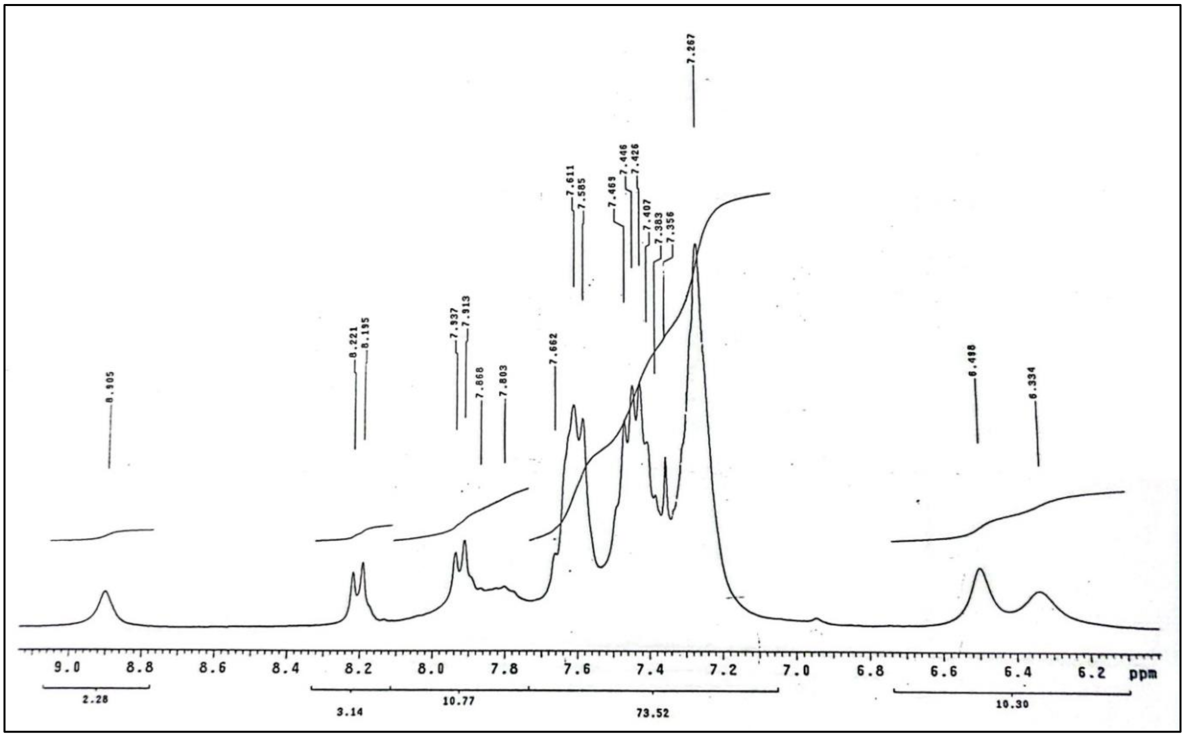

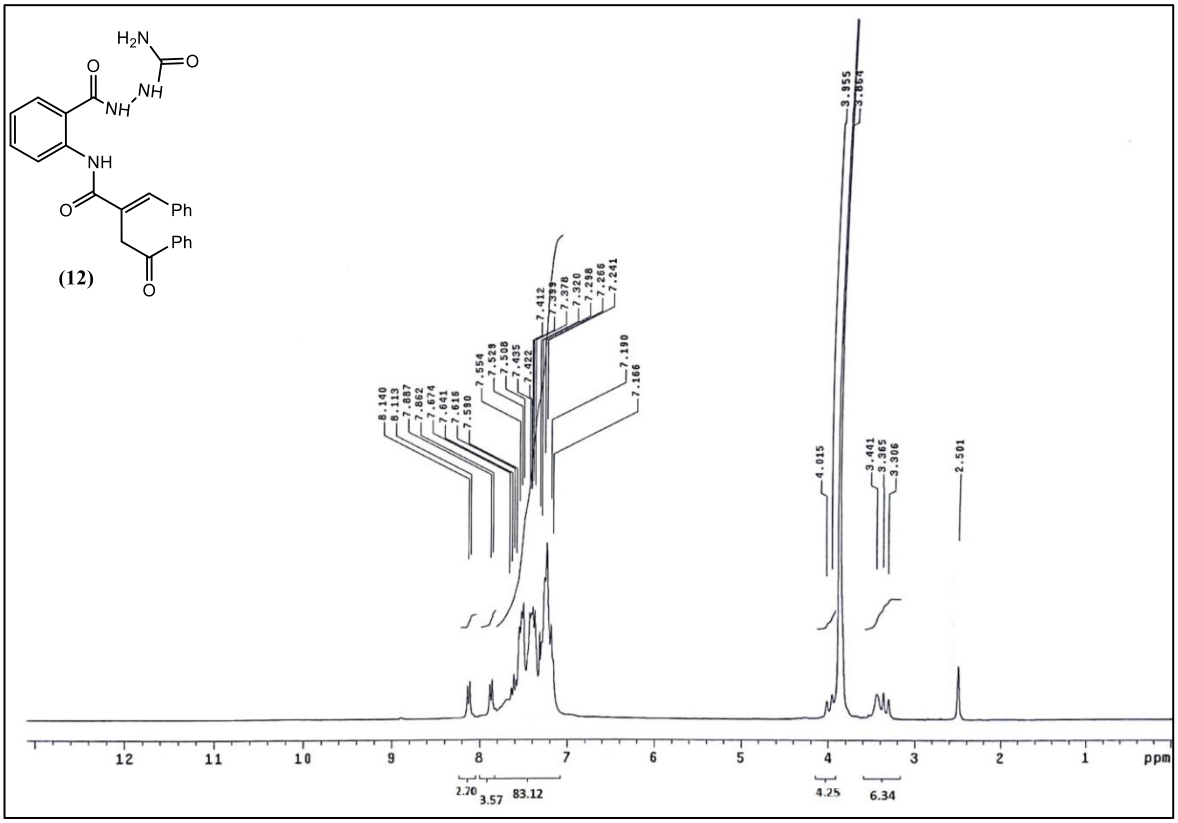

^{1}H-NMR (DMSO-*d6*) + D_2O spectrum compound of **12**

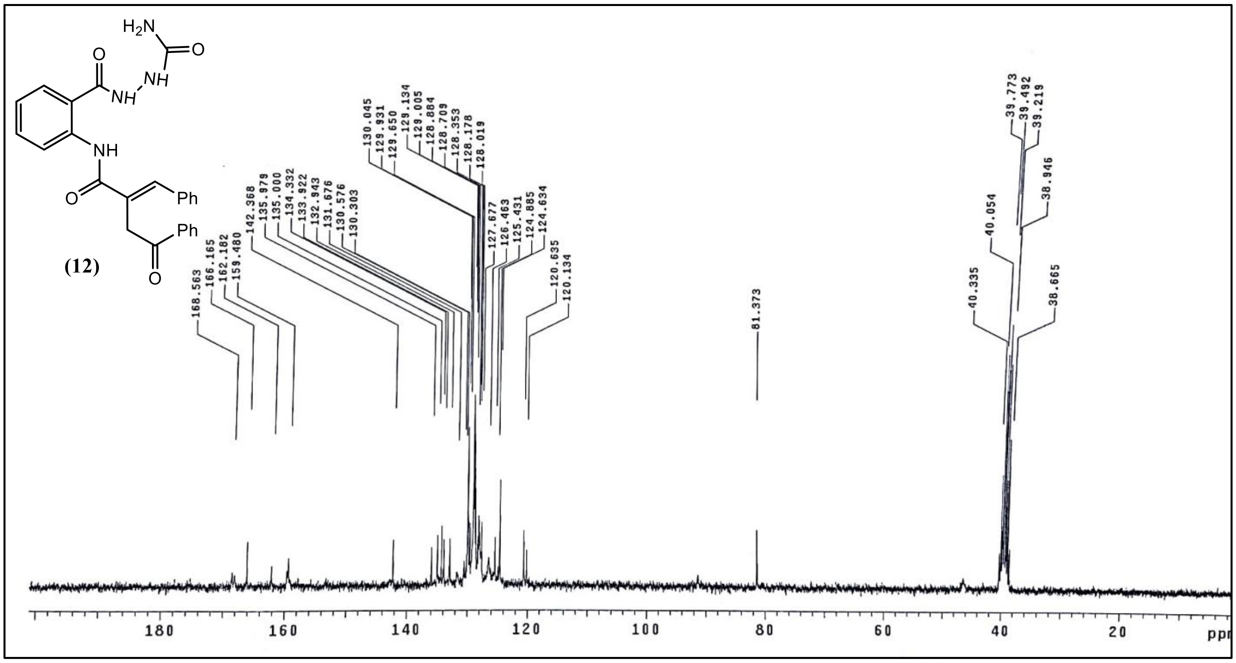

^{13}C-NMR (DMSO-*d*^{6}) spectrum compound of **12**

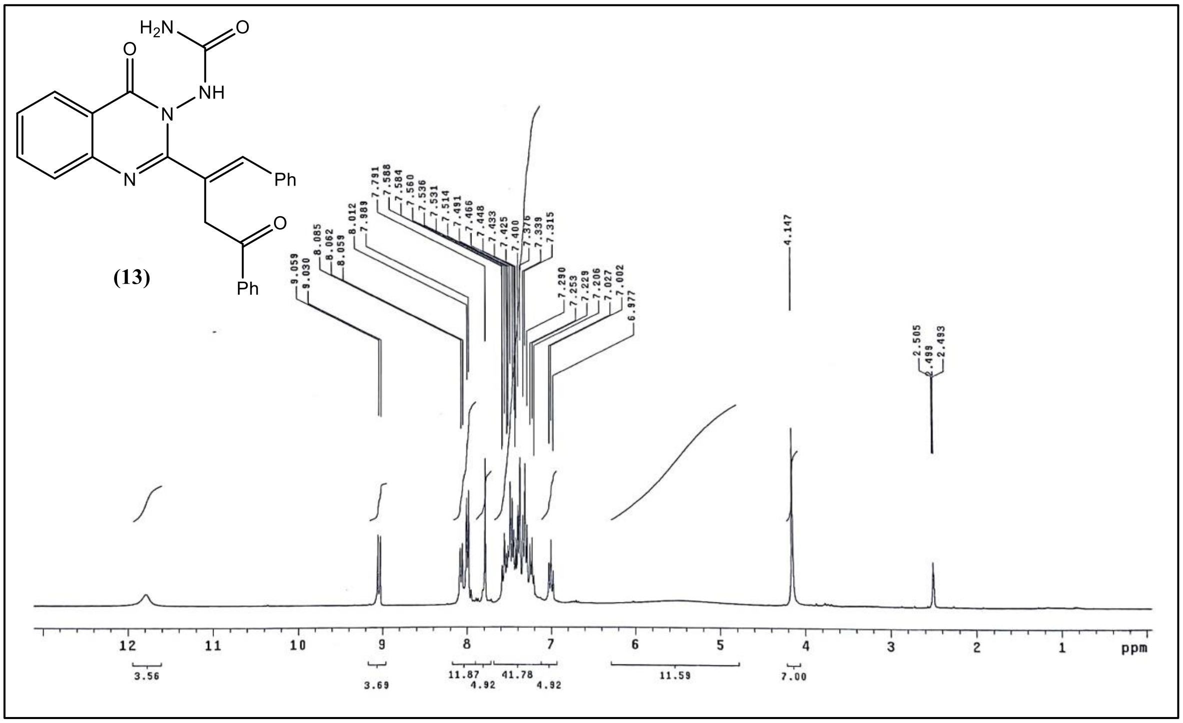

^{1}H-NMR (DMSO-*d6*) spectrum compound of **13**

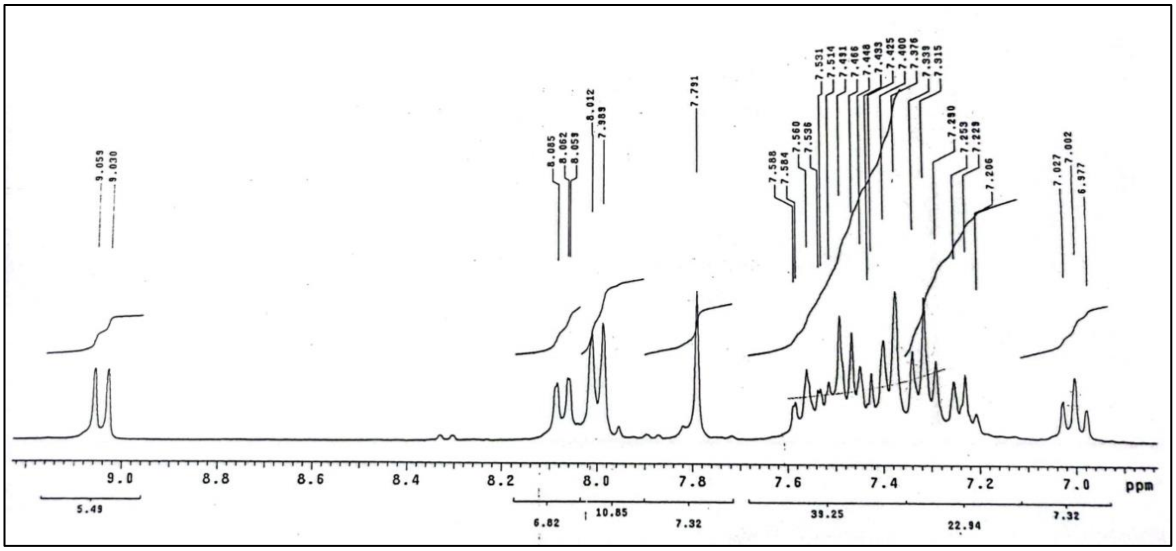

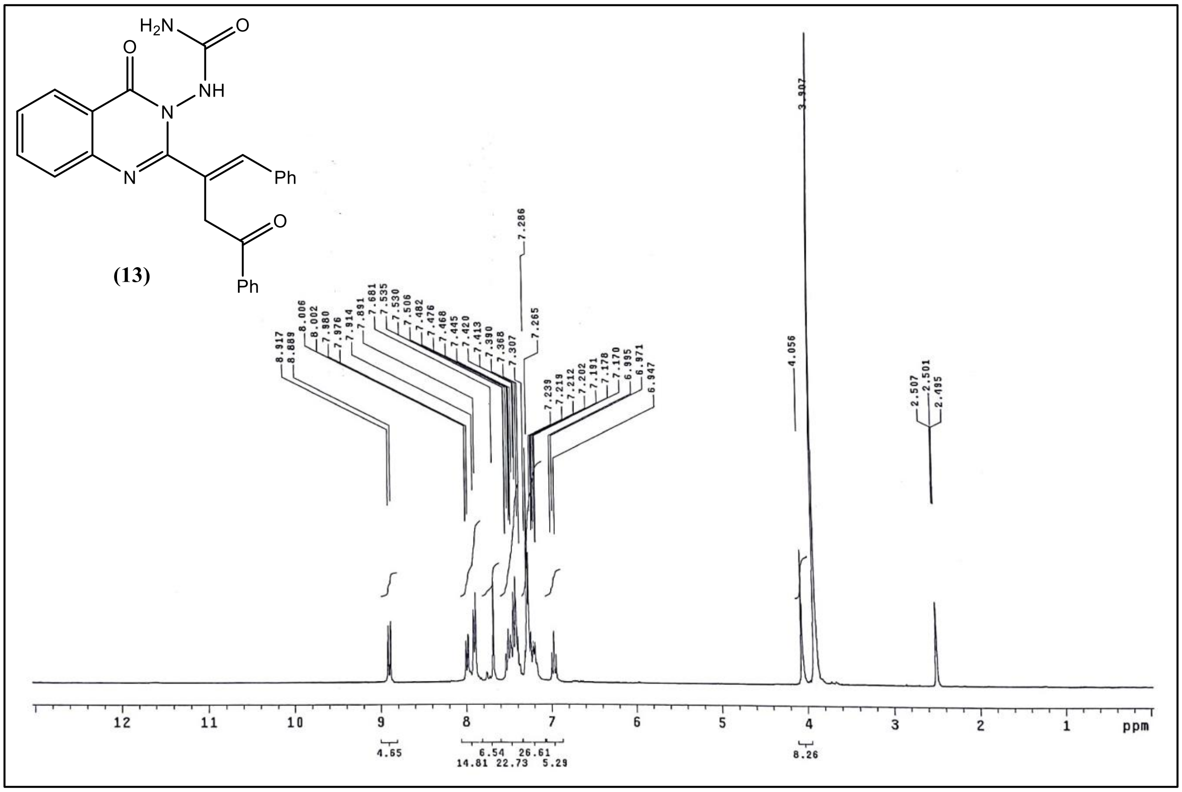

^{1}H-NMR (DMSO-*d6*) + D_2O spectrum compound of **13**

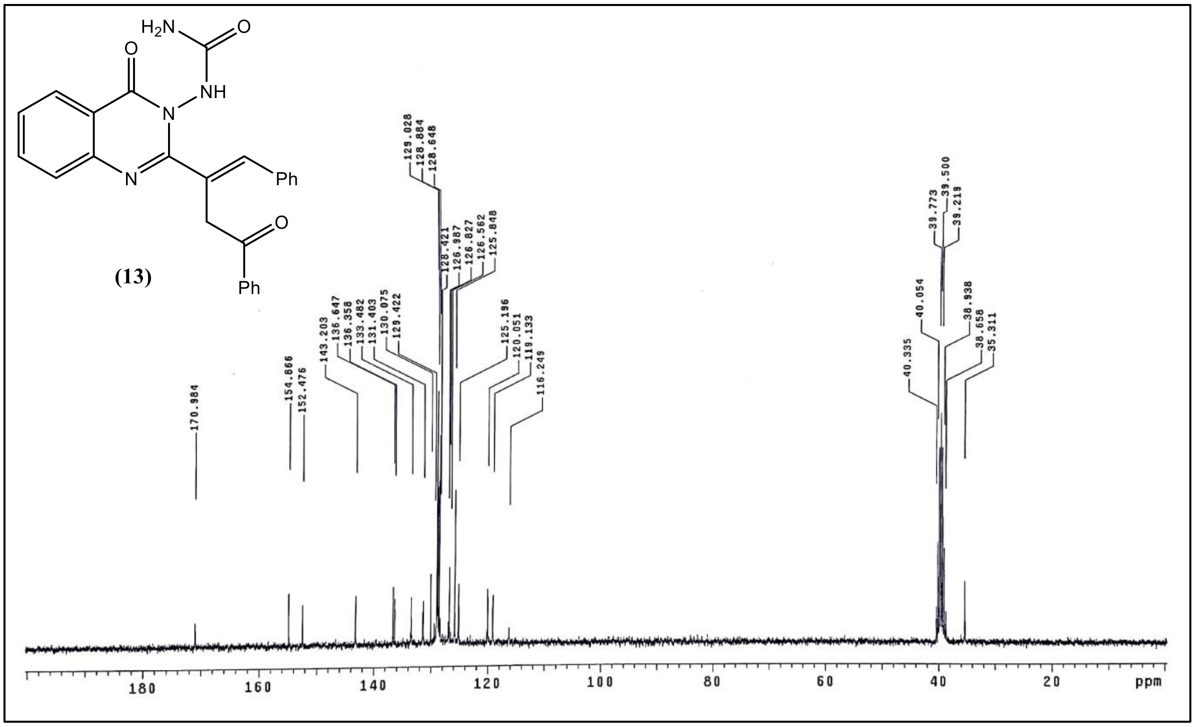

^{13}C-NMR (DMSO-*d*^{6}) spectrum compound of **13**

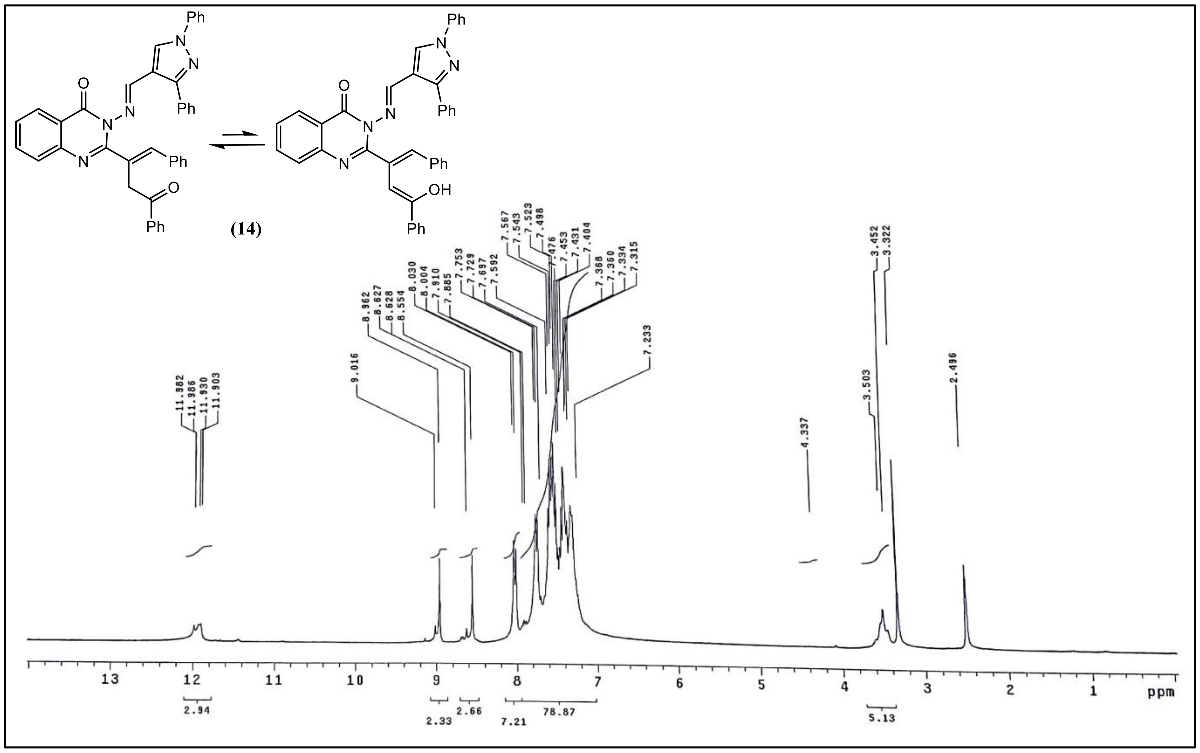

^{1}H-NMR (DMSO-*d6*) spectrum compound of **14**

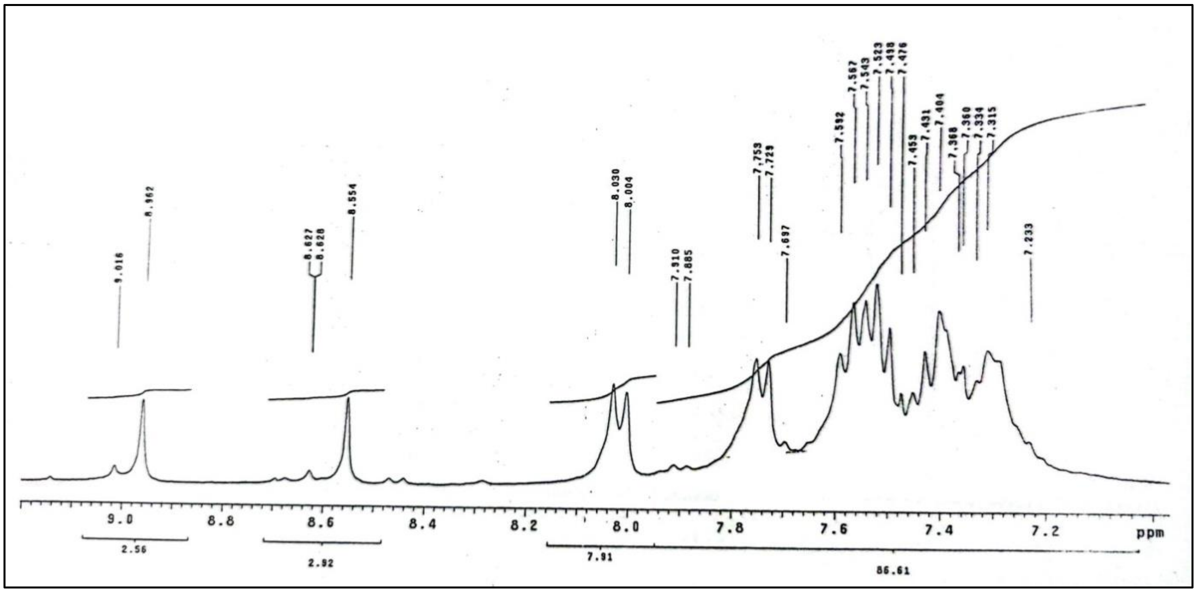

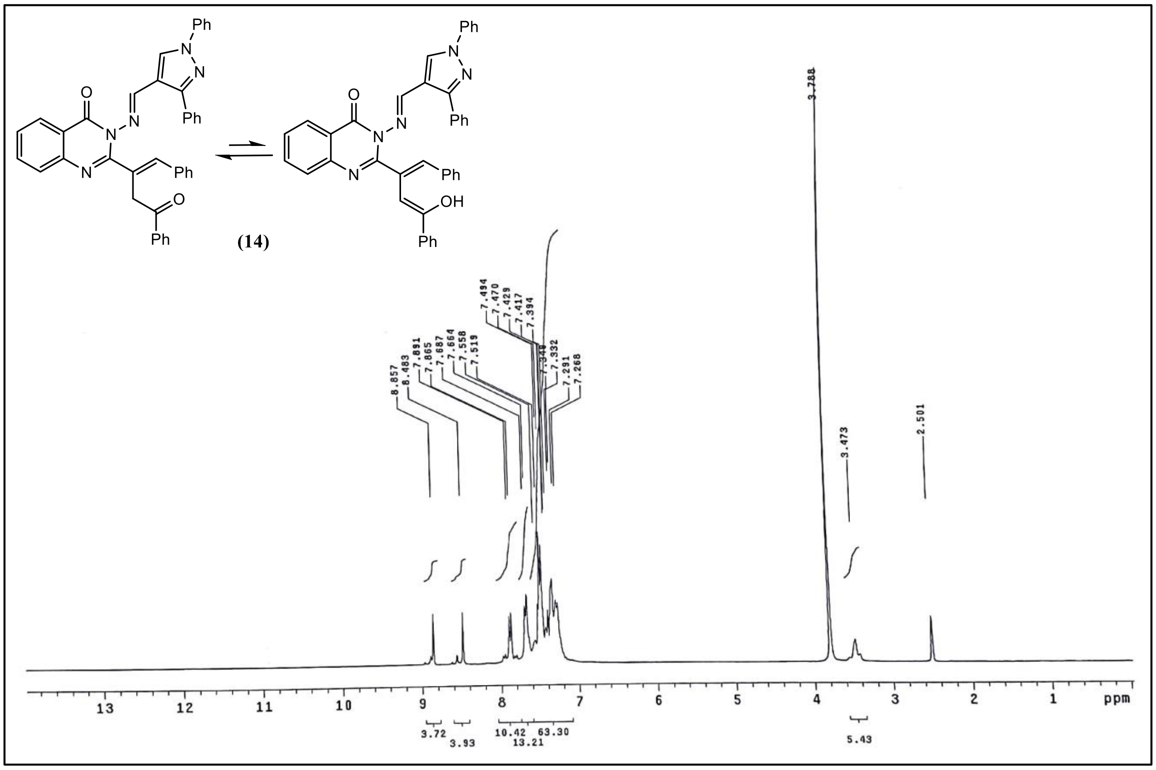

^{1}H-NMR (DMSO-*d6*) + D_2O spectrum compound of **14**

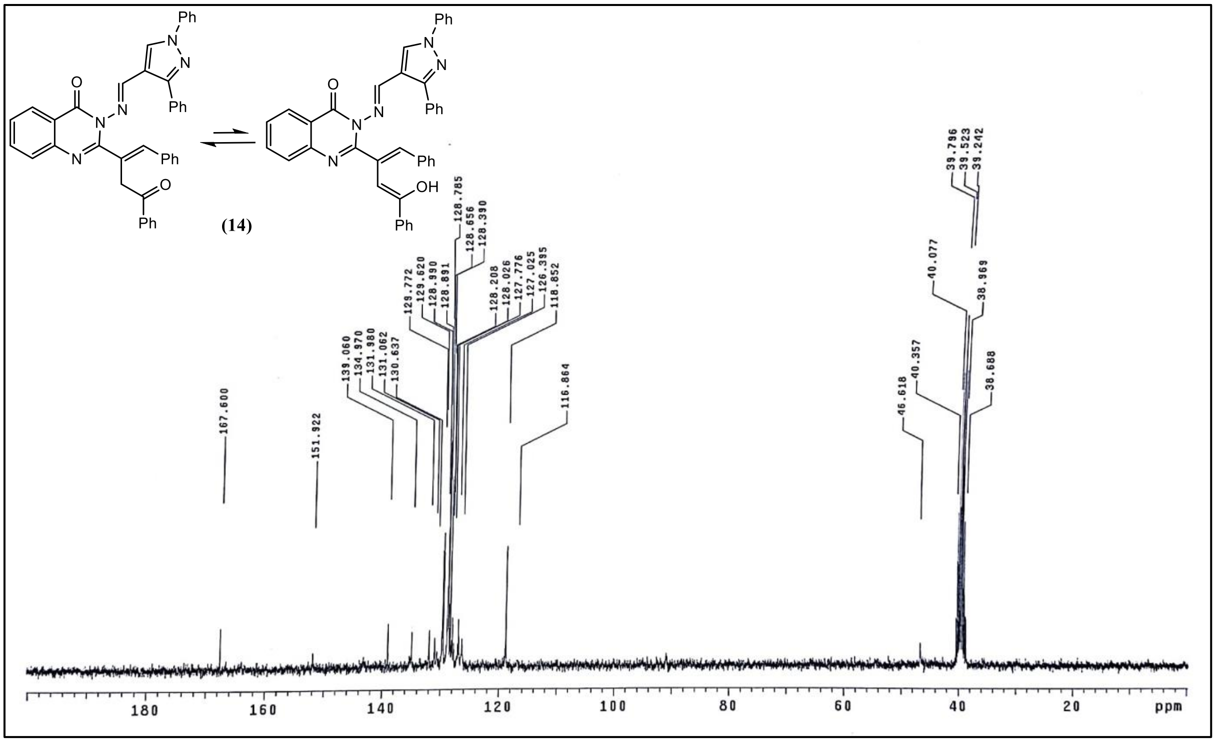

^{13}C-NMR (DMSO-*d*^{6}) spectrum compound of **14**

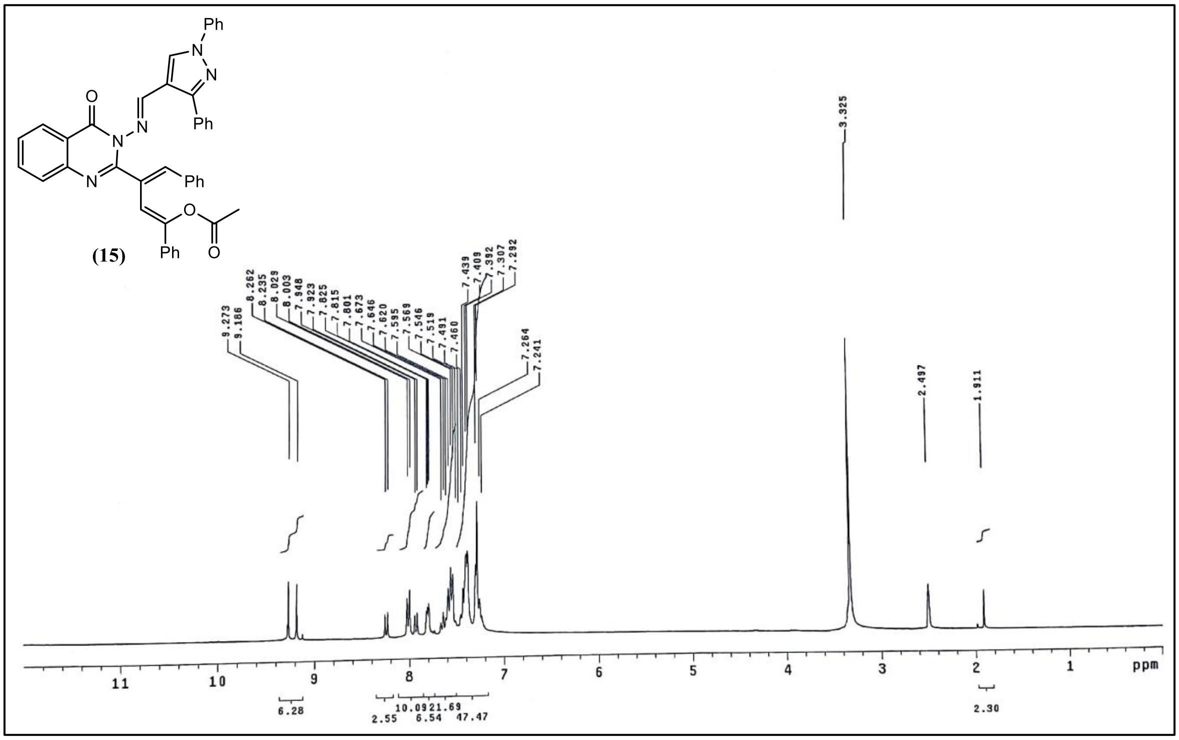

^{1}H-NMR (DMSO-*d6*) spectrum compound of **15**

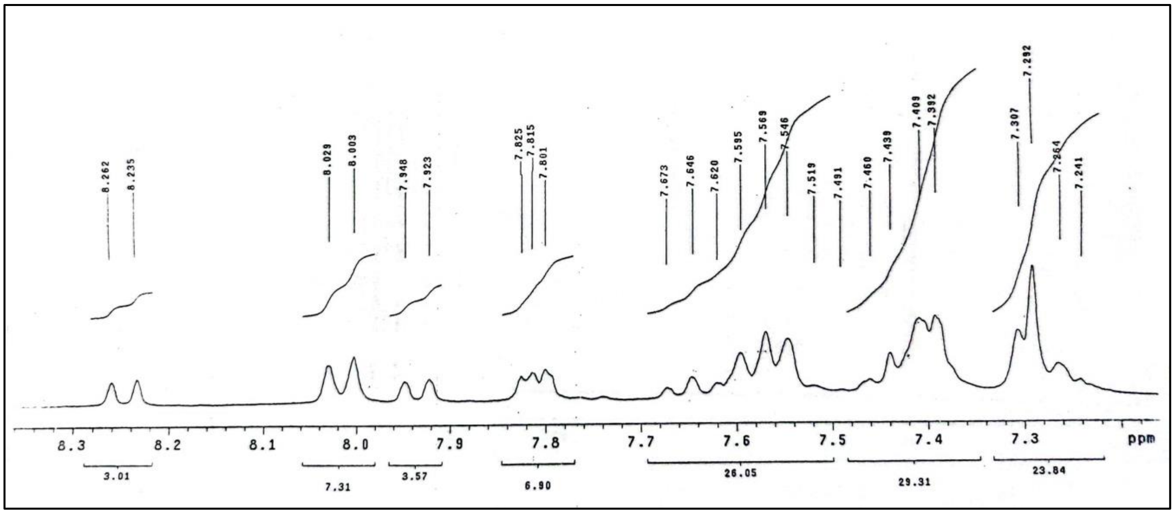

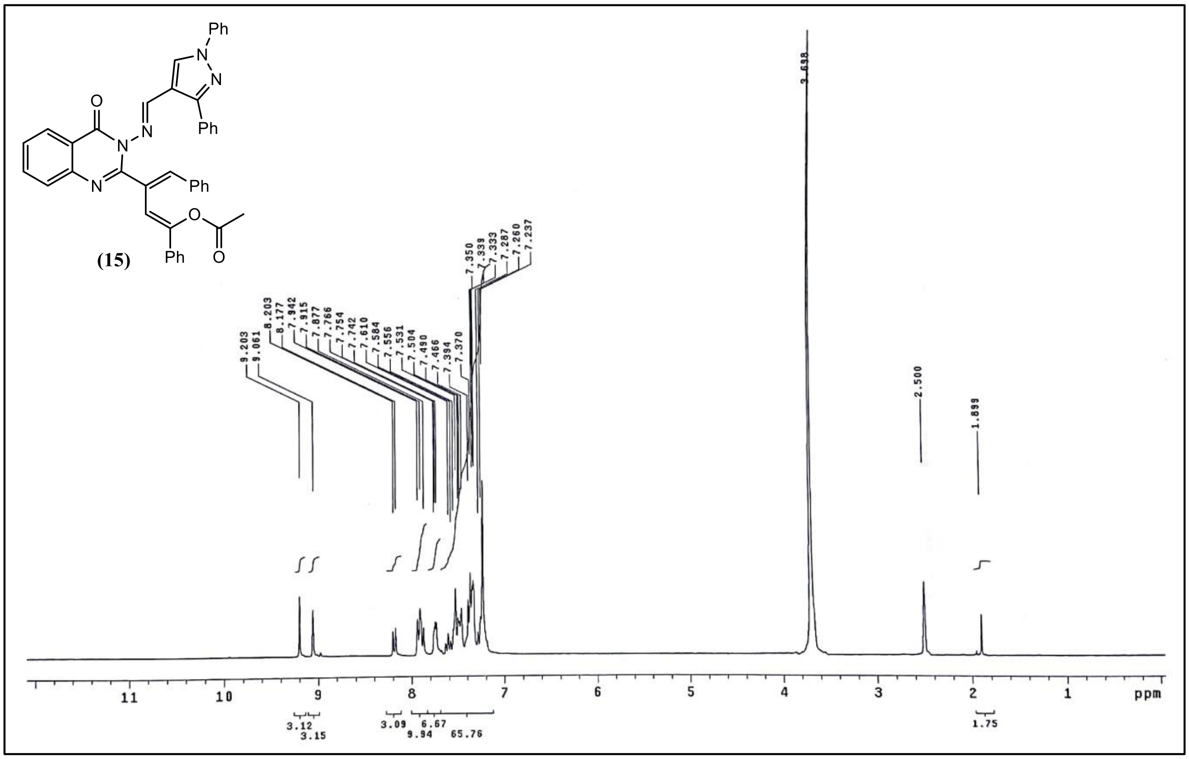

^{1}H-NMR (DMSO-*d6*) + D_2O spectrum compound of **15**

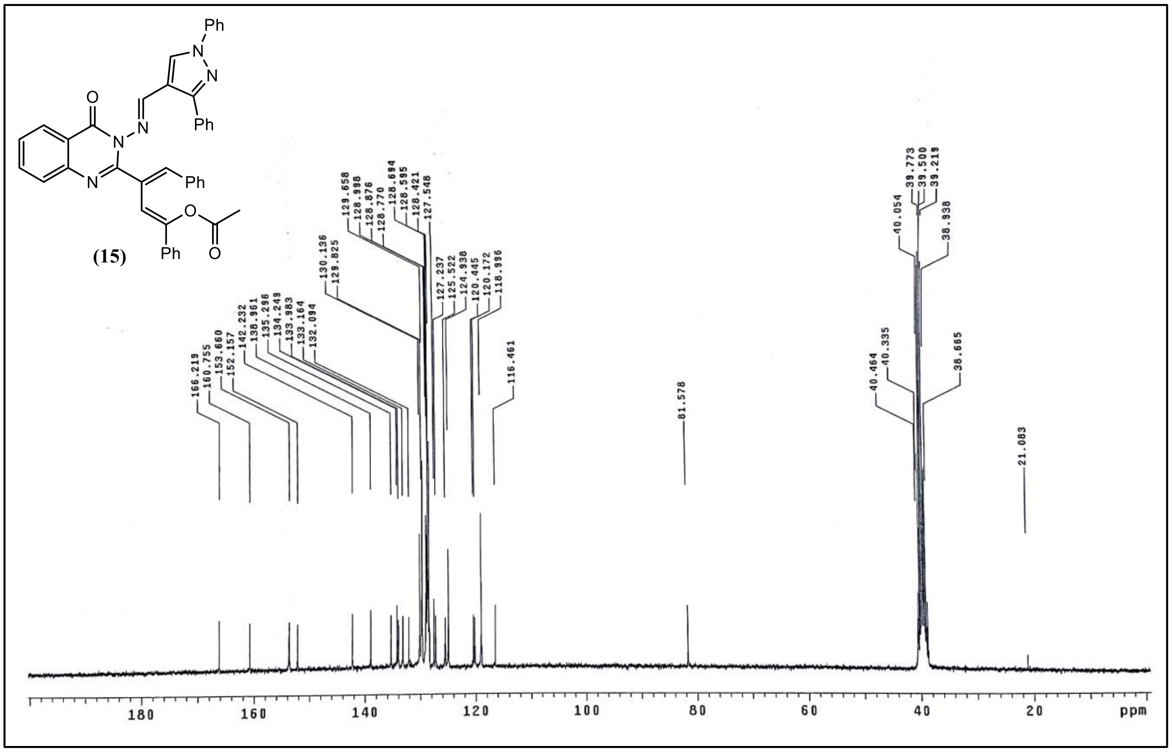

^{13}C-NMR (DMSO-*d*^{6}) spectrum compound of **15**
