# Supplementary material for: Synthesis, comprehensive in silico studies, and cytotoxicity evaluation of novel quinazolinone derivatives as potential anticancer agents
Source: Sci Rep. 2025 Jul 3;15:23697. doi: 10.1038/s41598-025-08062-7 (PMC12229541; doi:10.1038/s41598-025-08062-7)
Supplement: Supplementary file 3 — Supplementary Material 3 [file 41598_2025_8062_MOESM3_ESM.pdf]

Supplementary Data File 2: Molecular Docking Interaction Profile and ADME Evaluation of Novel Quinazolinone Derivatives Against Cancer Targets

This dataset presents the molecular docking interactions of newly synthesized quinazolinone derivatives with key cancer-related targets: Topoisomerase II (5ZAD), VEGFR2 (3WZE), c-Met (3U6I), EGFR (1M17), and Estrogen Receptor Alpha (3ERT). It includes detailed ligand–receptor binding interactions, highlighting hydrogen bonding, π–π stacking, and hydrophobic contacts, along with associated binding energies (E, kcal/mol) and interaction distances (Å). In addition to molecular docking results, the dataset also provides *in silico* ADME profiling of the compounds, encompassing key pharmacokinetic parameters such as lipophilicity, solubility, permeability, metabolic stability, and drug-likeness. These integrated findings offer insights into the molecular mechanisms and pharmacokinetic behavior underlying the anticancer potential of these compounds, supporting their further optimization as promising therapeutic inhibitors.

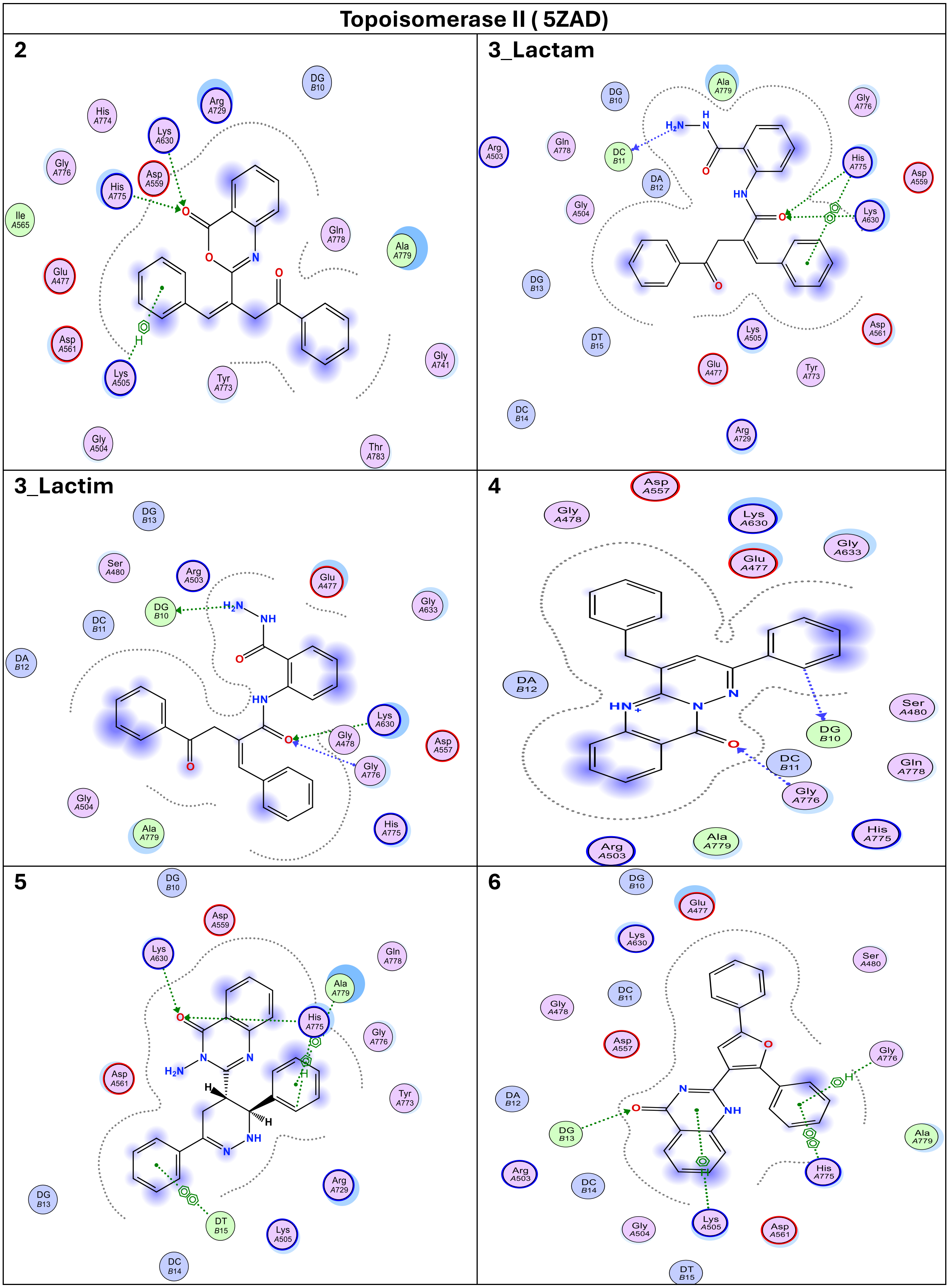

Topoisomerase II (5ZAD)
2
DG B10
Arg A729
His A774
Lys A630
Gly A776
Asp A559
His A775
Ile A565
Gln A778
Ala A779
Glu A477
Asp A561
Lys A505
Tyr A773
Gly A741
Gly A504
Thr A783
3_Lactam
Ala A779
DG B10
Gly A776
Arg A503
Gln A778
DC B11
His A775
Asp A559
DA B12
Gly A504
Lys A630
DG B13
DT B15
Lys A505
Asp A561
Glu A477
Tyr A773
DC B14
Arg A729
3_Lactim
DG B13
Ser A480
Arg A503
Glu A477
Gly A633
DG B10
DC B11
DA B12
Lys A630
Gly A478
Asp A557
Gly A776
Gly A504
Ala A779
His A775
4
Asp A557
Gly A478
Lys A630
Gly A633
Glu A477
DA B12
Ser A480
DG B10
DC B11
Gln A778
Gly A776
Arg A503
Ala A779
His A775
5
DG B10
Asp A559
Lys A630
Gln A778
Ala A779
His A775
Gly A776
Asp A561
Tyr A773
Arg A729
DG B13
DT B15
Lys A505
DC B14
6
DG B10
Glu A477
Lys A630
Ser A480
DC B11
Gly A478
Asp A557
Gly A776
DA B12
DG B13
His A775
Ala A779
Arg A503
DC B14
Lys A505
Asp A561
Gly A504
DT B15

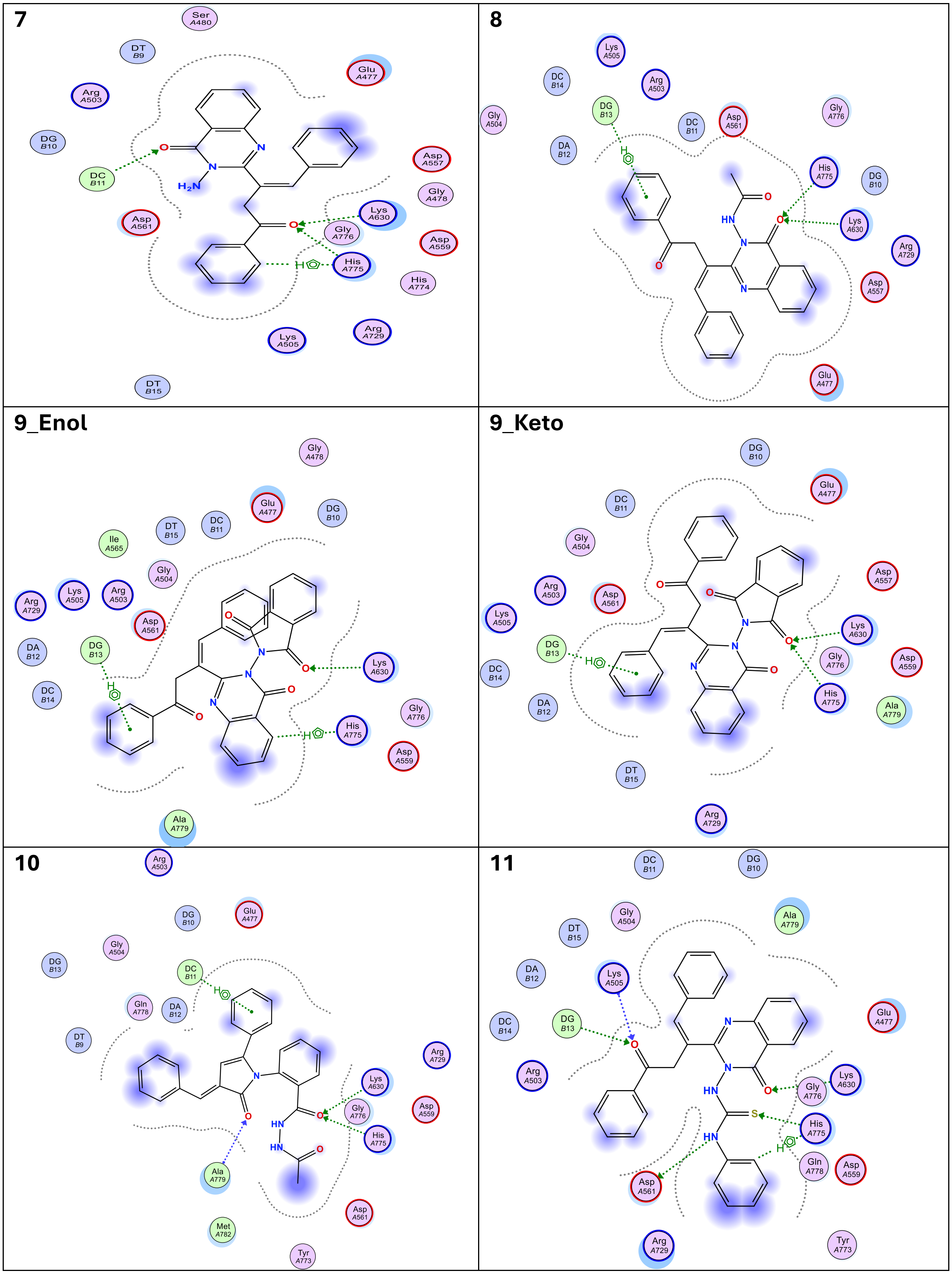

7
Ser A480
DT B9
Glu A477
Arg A503
DG B10
DC B11
Asp A557
Gly A478
Lys A630
Asp A561
Gly A776
Asp A559
His A775
His A774
Lys A505
Arg A729
DT B15
8
Lys A505
DC B14
Arg A503
DG B13
Gly A504
DC B11
Asp A561
Gly A776
DA B12
His A775
DG B10
Lys A630
Arg A729
Asp A557
Glu A477
9_Enol
Gly A478
Glu A477
DG B10
DC B11
DT B15
Ile A565
Gly A504
Arg A729
Lys A505
Arg A503
Asp A561
DA B12
DG B13
Lys A630
DC B14
Gly A776
His A775
Asp A559
Ala A779
9_Keto
DG B10
Glu A477
DC B11
Gly A504
Asp A557
Arg A503
Asp A561
Lys A505
Lys A630
DG B13
Gly A776
Asp A559
DC B14
His A775
DA B12
Ala A779
DT B15
Arg A729
10
Arg A503
DG B10
Glu A477
Gly A504
DG B13
DC B11
Gln A778
DA B12
DT B9
Arg A729
Lys A630
Asp A559
Gly A776
His A775
Ala A779
Asp A561
Met A782
Tyr A773
11
DC B11
DG B10
Gly A504
Ala A779
DT B15
DA B12
Lys A505
Glu A477
DC B14
DG B13
Arg A503
Lys A630
Gly A776
His A775
Gln A778
Asp A559
Asp A561
Arg A729
Tyr A773

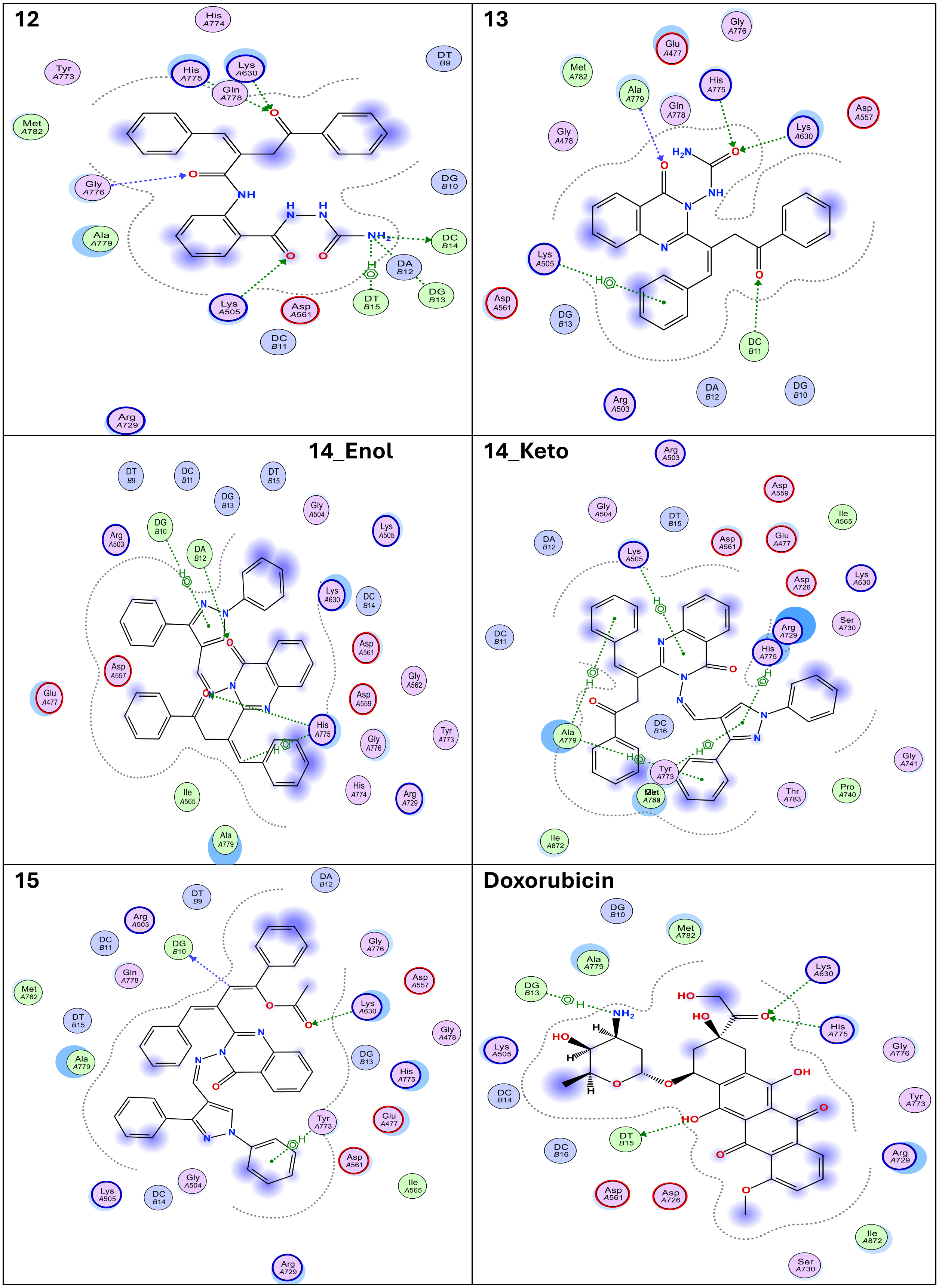
12
His A774
Tyr A773
His A775
Lys A630
Gln A778
DT B9
Met A782
Gly A776
DG B10
Ala A779
DC B14
DA B12
DG B13
DT B15
Lys A505
Asp A561
DC B11
Arg A729
13
Gly A776
Glu A477
Met A782
Ala A779
His A775
Gln A778
Asp A557
Lys A630
Gly A478
Lys A505
Asp A561
DG B13
DC B11
DA B12
DG B10
Arg A503
14_Enol
DT B9
DC B11
DT B15
DG B13
Gly A504
Lys A505
Arg A503
DG B10
DA B12
Lys A630
DC B14
Asp A561
Asp A557
Gly A562
Glu A477
Asp A559
His A775
Gly A776
Tyr A773
Ile A565
His A774
Arg A729
Ala A779
14_Keto
Arg A503
Asp A559
Gly A504
DT B15
Ile A565
DA B12
Lys A505
Asp A561
Glu A477
Asp A726
Lys A630
Arg A729
Ser A730
DC B11
His A775
DC B16
Ala A779
Tyr A773
Gly A741
Thr A783
Pro A740
Ile A872
15
DA B12
DT B9
Arg A503
DC B11
DG B10
Gly A776
Gln A778
Asp A557
Met A782
Lys A630
Gly A478
DT B15
DG B13
Ala A779
His A775
Tyr A773
Glu A477
Asp A561
Ile A565
Lys A505
DC B14
Gly A504
Arg A729
Doxorubicin
DG B10
Met A782
Ala A779
Lys A630
DG B13
His A775
Lys A505
Gly A776
DC B14
Tyr A773
DC B16
DT B15
Arg A729
Asp A561
Asp A726
Ile A872
Ser A730

Topoisomerase II (5ZAD)				
**Ligand**	**Residues**	**Interaction Type**	**Distance (Å)**	**E (kcal/mol)**
2	LYS 630 (A)	H-acceptor	2.98	-11.5
	HIS 775 (A)	H-acceptor	2.92	-5.7
	LYS 505 (A)	pi-H	3.86	-1.1
3_Lactam	DC 11 (F)	H-donor	3.22	-1.9
	LYS 630 (A)	H-acceptor	3.47	-1.6
	HIS 775 (A)	H-acceptor	2.9	-7.1
	HIS 775 (A)	pi-pi	4.09	
3_Lactim	DG 10 (F)	H-donor	3.57	-0.5
	LYS 630 (A)	H-acceptor	3.48	-0.8
	GLY 776 (A)	H-acceptor	2.98	-0.8
4	DG 10 (F)	H-donor	3.25	-0.5
	GLY 776 (A)	H-acceptor	3.17	-0.5
5	LYS 630 (A)	H-acceptor	2.86	-15.8
	HIS 775 (A)	H-acceptor	2.98	-4.9
	HIS 775 (A)	H-pi	3.61	-0.5
	ALA 779 (A)	pi-H	4.24	-0.5
	DT 15 (F)	pi-pi	4.15	
6	DG 13 (F)	H-acceptor	3.2	-1.6
	DG 13 (F)	H-acceptor	2.87	-2.7
	LYS 505 (A)	pi-H	4.17	-0.5
	LYS 505 (A)	pi-H	4.51	-1.7
	GLY 776 (A)	pi-H	4.94	-0.6
	HIS 775 (A)	pi-pi	4.18	
7	DC 11 (F)	H-acceptor	2.77	-4.2
	LYS 630 (A)	H-acceptor	3.12	-2.8
	HIS 775 (A)	H-acceptor	2.86	-1.9
	HIS 775 (A)	H-pi	4.05	-0.5
8	LYS 630 (A)	H-acceptor	3.51	-1.2
	HIS 775 (A)	H-acceptor	2.86	-5.3
	DG 13 (F)	pi-H	3.59	-0.8
9_Enol	LYS 630 (A)	H-acceptor	3.35	-2.3
	HIS 775 (A)	H-pi	3.82	-0.5
	DG 13 (F)	pi-H	4.01	-1.1
9_Keto	LYS 630 (A)	H-acceptor	3.07	-6.9
	HIS 775 (A)	H-acceptor	3.04	-3
	DG 13 (F)	pi-H	3.6	-0.5
10	LYS 630 (A)	H-acceptor	2.99	-9.8
	HIS 775 (A)	H-acceptor	3	-3.7

	ALA 779 (A)	H-acceptor	3.45	-1.2
	DC 11 (F)	pi-H	4.26	-0.5
11	ASP 561 (A)	H-donor	3.27	-0.6
	LYS 630 (A)	H-acceptor	3.09	-0.8
	LYS 505 (A)	H-acceptor	3.1	-2.5
	DG 13 (F)	H-acceptor	3.08	-2.8
	HIS 775 (A)	H-acceptor	3.88	-0.7
	HIS 775 (A)	H-pi	3.8	-0.7
12	DC 14 (F)	H-donor	2.83	-1.1
	LYS 505 (A)	H-acceptor	3.43	-0.6
	GLY 776 (A)	H-acceptor	3.31	-1.3
	LYS 630 (A)	H-acceptor	2.89	-3
	HIS 775 (A)	H-acceptor	3.16	-5
	DG 13 (F)	H-pi	4.67	-1.2
	DT 15 (F)	H-pi	3.44	-0.8
13	DC 11 (F)	H-acceptor	3.15	-0.8
	ALA 779 (A)	H-acceptor	3.46	-0.7
	LYS 630 (A)	H-acceptor	3.38	-3.3
	HIS 775 (A)	H-acceptor	3.3	-2.4
	LYS 505 (A)	pi-H	4.54	-0.8
14_Enol	DA 12 (F)	H-acceptor	3.12	-1.4
	HIS 775 (A)	H-acceptor	3.04	-0.7
	HIS 775 (A)	H-pi	4.1	-1.6
	DG 10 (F)	pi-H	3.68	-1.1
14_Keto	LYS 505 (A)	pi-H	4.27	-0.6
	ARG 729 (A)	pi-H	4.48	-1.3
	TYR 773 (A)	pi-H	4.06	-1.6
	ALA 779 (A)	pi-H	3.75	-0.9
	ALA 779 (A)	pi-H	4	-0.8
15	DG 10 (F)	H-donor	3.4	-0.7
	LYS 630 (A)	H-acceptor	2.92	-4.1
	TYR 773 (A)	pi-H	4.08	-0.7
Doxorubicin	DT 15 (F)	H-donor	2.94	-1.2
	LYS 630 (A)	H-acceptor	3.48	-2
	HIS 775 (A)	H-acceptor	2.93	-5.2
	DG 13 (F)	H-pi	4.85	-0.5

VEGFR2 (3WZE)

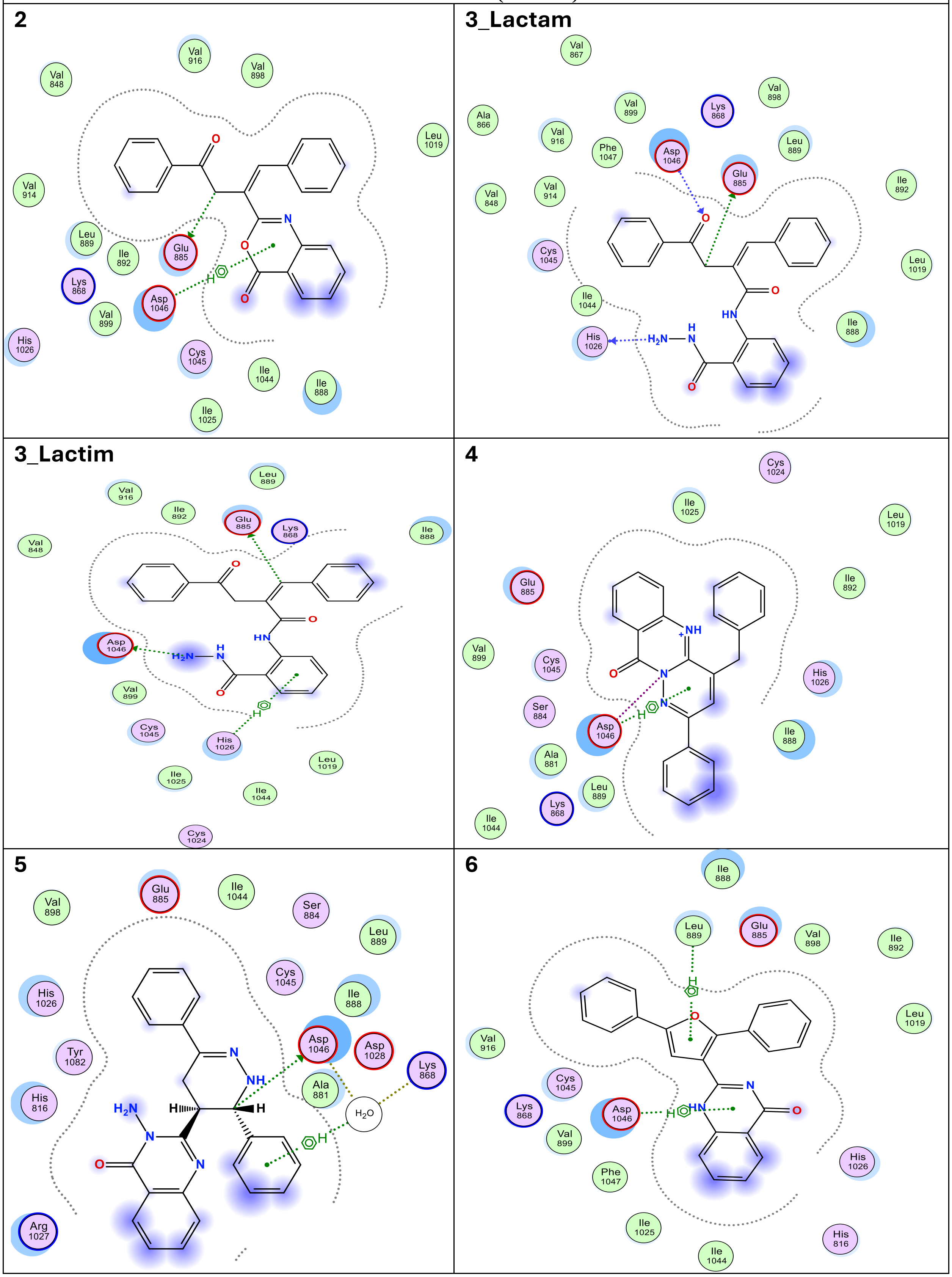
2
Val 916
Val 898
Val 848
Leu 1019
Val 914
Leu 889
Ile 892
Glu 885
Lys 868
Asp 1046
Val 899
His 1026
Cys 1045
Ile 1044
Ile 888
Ile 1025
3_Lactam
Val 867
Val 899
Lys 868
Val 898
Ala 866
Val 916
Phe 1047
Asp 1046
Leu 889
Glu 885
Ile 892
Val 848
Val 914
Cys 1045
Leu 1019
Ile 1044
Ile 888
His 1026
3_Lactim
Leu 889
Val 916
Ile 892
Glu 885
Lys 868
Ile 888
Val 848
Asp 1046
Val 899
Cys 1045
His 1026
Leu 1019
Ile 1025
Ile 1044
Cys 1024
4
Cys 1024
Ile 1025
Leu 1019
Glu 885
Ile 892
Val 899
Cys 1045
His 1026
Ser 884
Asp 1046
Ile 888
Ala 881
Leu 889
Lys 868
Ile 1044
5
Val 898
Glu 885
Ile 1044
Ser 884
Leu 889
Cys 1045
His 1026
Ile 888
Tyr 1082
Asp 1046
Asp 1028
Lys 868
Ala 881
His 816
H2O
Arg 1027
6
Ile 888
Leu 889
Glu 885
Val 898
Ile 892
Leu 1019
Val 916
Cys 1045
Lys 868
Asp 1046
Val 899
His 1026
Phe 1047
Ile 1025
His 816
Ile 1044

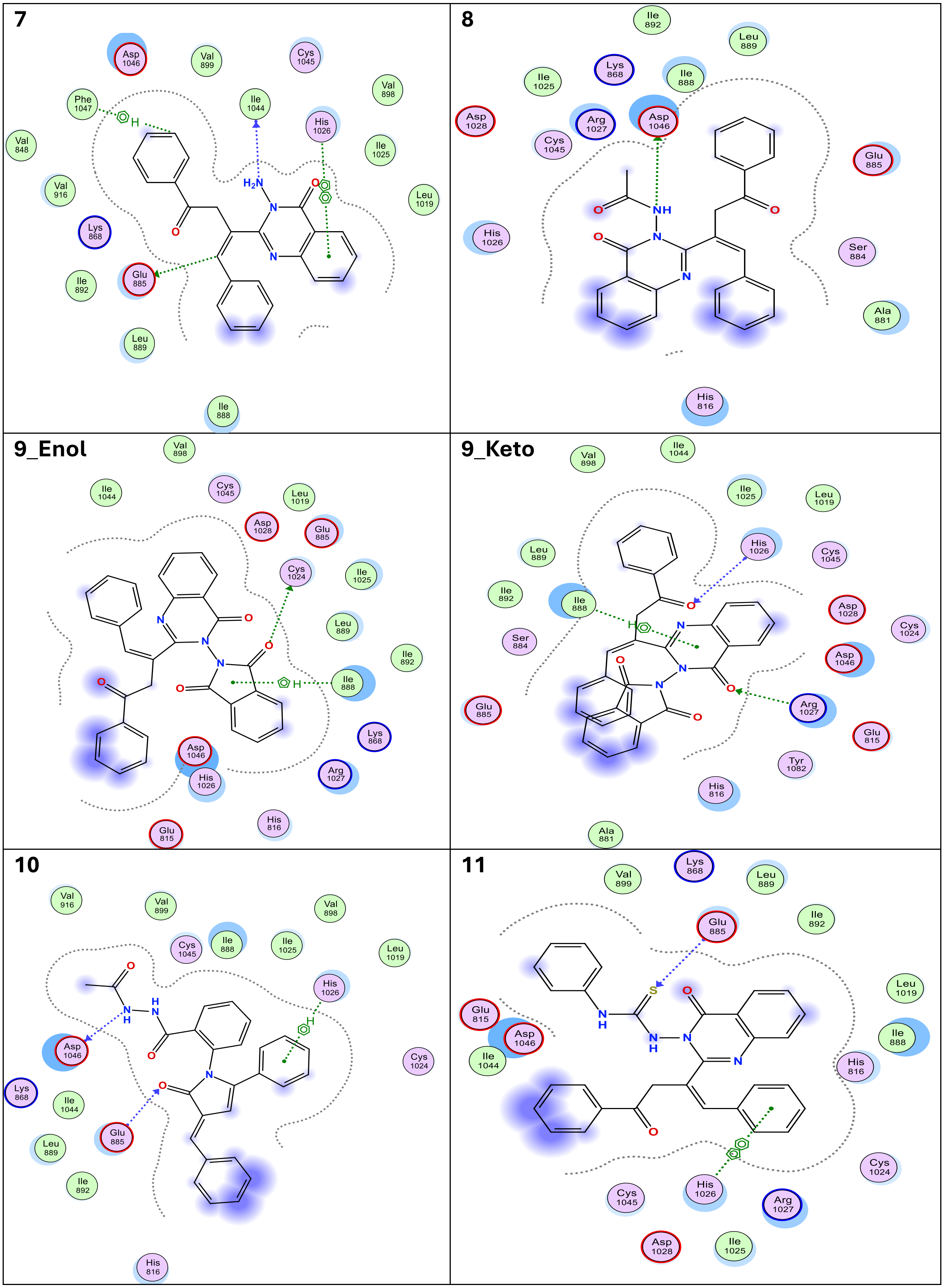
7
Asp 1046
Val 899
Cys 1045
Val 898
Phe 1047
Ile 1044
His 1026
Ile 1025
Val 848
Val 916
Leu 1019
Lys 868
Glu 885
Ile 892
Leu 889
Ile 888
8
Ile 892
Leu 889
Lys 868
Ile 888
Ile 1025
Asp 1028
Arg 1027
Asp 1046
Cys 1045
Glu 885
His 1026
Ser 884
Ala 881
His 816
9_Enol
Val 898
Ile 1044
Cys 1045
Leu 1019
Asp 1028
Glu 885
Cys 1024
Ile 1025
Leu 889
Ile 892
Ile 888
Lys 868
Asp 1046
His 1026
Arg 1027
His 816
Glu 815
9_Keto
Val 898
Ile 1044
Ile 1025
Leu 1019
His 1026
Cys 1045
Leu 889
Ile 892
Ile 888
Asp 1028
Cys 1024
Ser 884
Asp 1046
Glu 885
Arg 1027
Glu 815
Tyr 1082
His 816
Ala 881
10
Val 916
Val 899
Val 898
Cys 1045
Ile 888
Ile 1025
Leu 1019
His 1026
Asp 1046
Cys 1024
Lys 868
Ile 1044
Glu 885
Leu 889
Ile 892
His 816
11
Lys 868
Val 899
Leu 889
Glu 885
Ile 892
Leu 1019
Glu 815
Asp 1046
Ile 888
Ile 1044
His 816
Cys 1024
Cys 1045
His 1026
Arg 1027
Asp 1028
Ile 1025

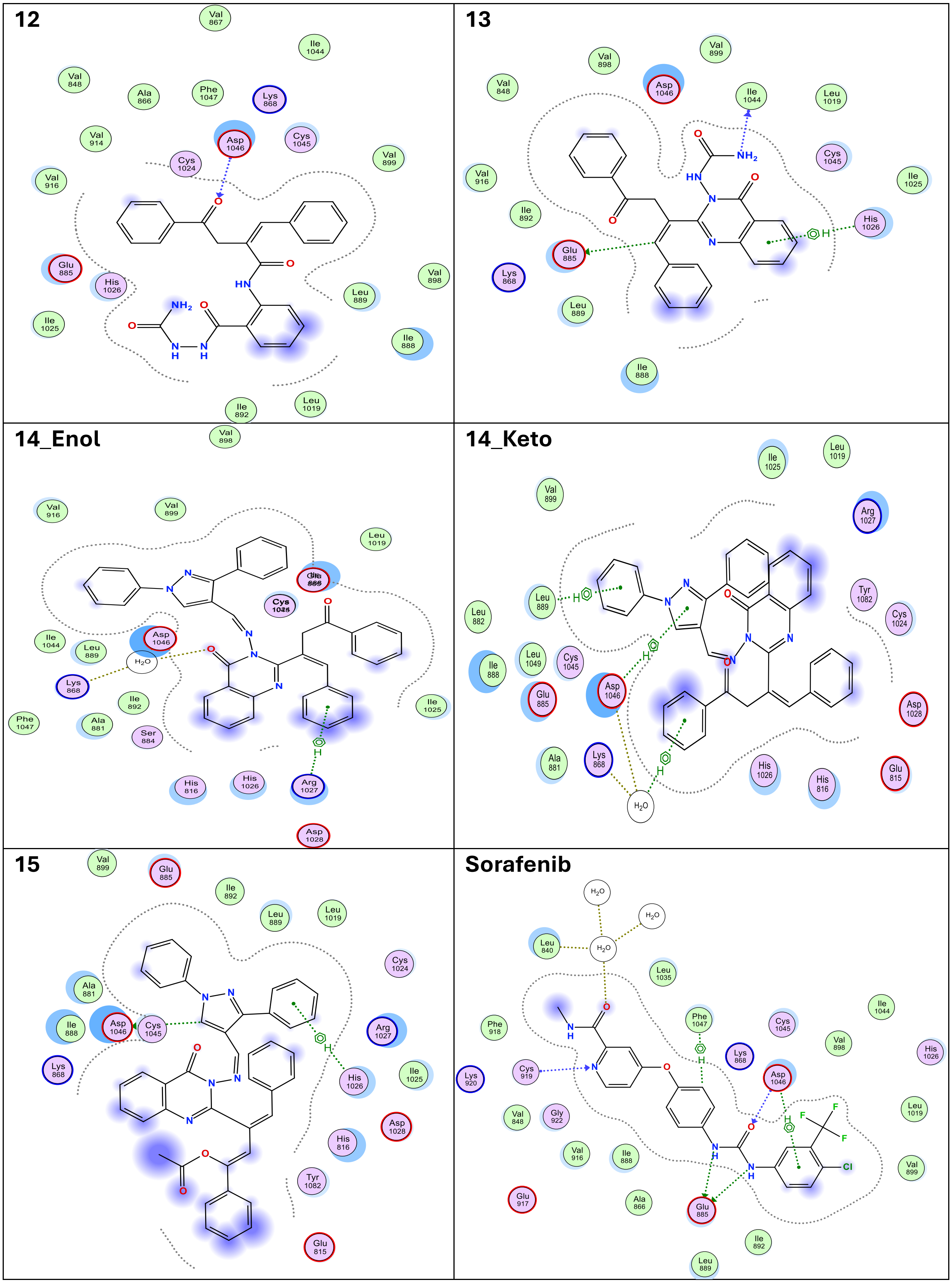

12
Val 867
Ile 1044
Val 848
Ala 866
Phe 1047
Lys 868
Val 914
Asp 1046
Cys 1045
Cys 1024
Val 899
Val 916
Glu 885
His 1026
Val 898
Leu 889
Ile 1025
Ile 888
Ile 892
Leu 1019
13
Val 899
Val 898
Val 848
Asp 1046
Ile 1044
Leu 1019
Cys 1045
Val 916
Ile 1025
Ile 892
His 1026
Glu 885
Lys 868
Leu 889
Ile 888
14_Enol
Val 898
Val 916
Val 899
Leu 1019
Cys 1045
Ile 1044
Leu 889
Asp 1046
H_2O
Lys 868
Ile 892
Phe 1047
Ala 881
Ser 884
His 816
His 1026
Arg 1027
Ile 1025
Asp 1028
14_Keto
Ile 1025
Leu 1019
Val 899
Arg 1027
Tyr 1082
Leu 889
Cys 1024
Leu 882
Leu 1049
Cys 1045
Ile 888
Asp 1046
Glu 885
Asp 1028
Ala 881
Lys 868
His 1026
His 816
Glu 815
H_2O
15
Val 899
Glu 885
Ile 892
Leu 889
Leu 1019
Cys 1024
Ala 881
Ile 888
Asp 1046
Cys 1045
Arg 1027
Lys 868
His 1026
Ile 1025
Asp 1028
His 816
Tyr 1082
Glu 815
Sorafenib
H_2O
Leu 840
Leu 1035
Ile 1044
Phe 918
Phe 1047
Cys 1045
Val 898
His 1026
Lys 920
Cys 919
Lys 868
Asp 1046
Leu 1019
Val 848
Gly 922
Val 916
Ile 888
Val 899
Glu 917
Ala 866
Glu 885
Ile 892
Leu 889

VEGFR2 (3WZE)				
Ligand	Residues	Interaction Type	Distance (Å)	E (kcal/mol)
2	GLU 885 (A)	H-donor	3.1	-0.6
	ASP 1046 (A)	pi-H	3.94	-0.6
3_Lactam	HIS 1026 (A)	H-donor	2.89	-2.7
	GLU 885 (A)	H-donor	3.1	-1.1
	ASP 1046 (A)	H-acceptor	2.75	-4.2
3_Lactim	ASP 1046 (A)	H-donor	3.02	-2.4
	GLU 885 (A)	H-donor	3.17	-0.5
	HIS 1026 (A)	pi-H	4.31	-0.5
4	ASP 1046 (A)	Ionic	3.8	-0.9
	ASP 1046 (A)	pi-H	4.35	-0.5
5	ASP 1046 (A)	H-donor	3.48	-0.7
	HOH 3064 (A)	pi-H	4.7	-0.5
6	LEU 889 (A)	pi-H	4.36	-0.6
	ASP 1046 (A)	pi-H	4.33	-0.5
7	ILE 1044 (A)	H-donor	3.39	-0.5
	GLU 885 (A)	H-donor	3.01	-0.6
	PHE 1047 (A)	H-pi	4.14	-0.5
	HIS 1026 (A)	pi-pi	4.34	
8	ASP 1046 (A)	H-donor	3.11	-3.1
9_Enol	CYS 1024 (A)	H-donor	3.64	-1.4
	ILE 888 (A)	pi-H	4.03	-0.6
9_Keto	ARG 1027 (A)	H-acceptor	2.98	-0.7
	ARG 1027 (A)	H-acceptor	3.01	-2.7
	HIS 1026 (A)	H-acceptor	3.31	-0.5
	ILE 888 (A)	pi-H	4.02	-0.5
10	ASP 1046 (A)	H-donor	3.03	-0.8
	GLU 885 (A)	H-acceptor	3.18	-0.5
	HIS 1026 (A)	pi-H	3.74	-0.9
11	GLU 885 (A)	H-acceptor	3.61	-0.9
	HIS 1026 (A)	pi-pi	4.08	
12	ASP 1046 (A)	H-acceptor	2.8	-4
13	GLU 885 (A)	H-donor	3.21	-0.6
	ILE 1044 (A)	H-donor	2.99	-1.3
	HIS 1026 (A)	pi-H	3.98	-0.5
14_Enol	HOH 3064 (A)	H-acceptor	2.81	-1.3
	ARG 1027 (A)	pi-H	3.76	-0.9
14_Keto	LEU 889 (A)	pi-H	4.02	-0.6
	ASP 1046 (A)	pi-H	3.71	-0.6
	HOH 3064 (A)	pi-H	4.35	-0.6

**15**	**ASP 1046 (A)**	**H-donor**	**3.31**	**-0.8**
	**HIS 1026 (A)**	**pi-H**	**4.14**	**0**
**Sorafenib**	**GLU 885 (A)**	**H-donor**	**2.84**	**-5**
	**GLU 885 (A)**	**H-donor**	**3.02**	**-4.8**
	**ASP 1046 (A)**	**H-acceptor**	**3**	**-1.2**
	**HOH 3089 (A)**	**H-acceptor**	**2.82**	**-1.7**
	**CYS 919 (A)**	**H-acceptor**	**3.28**	**-1.8**
	**PHE 1047 (A)**	**H-pi**	**3.8**	**-0.8**
	**ASP 1046 (A)**	**pi-H**	**4.22**	**-0.5**

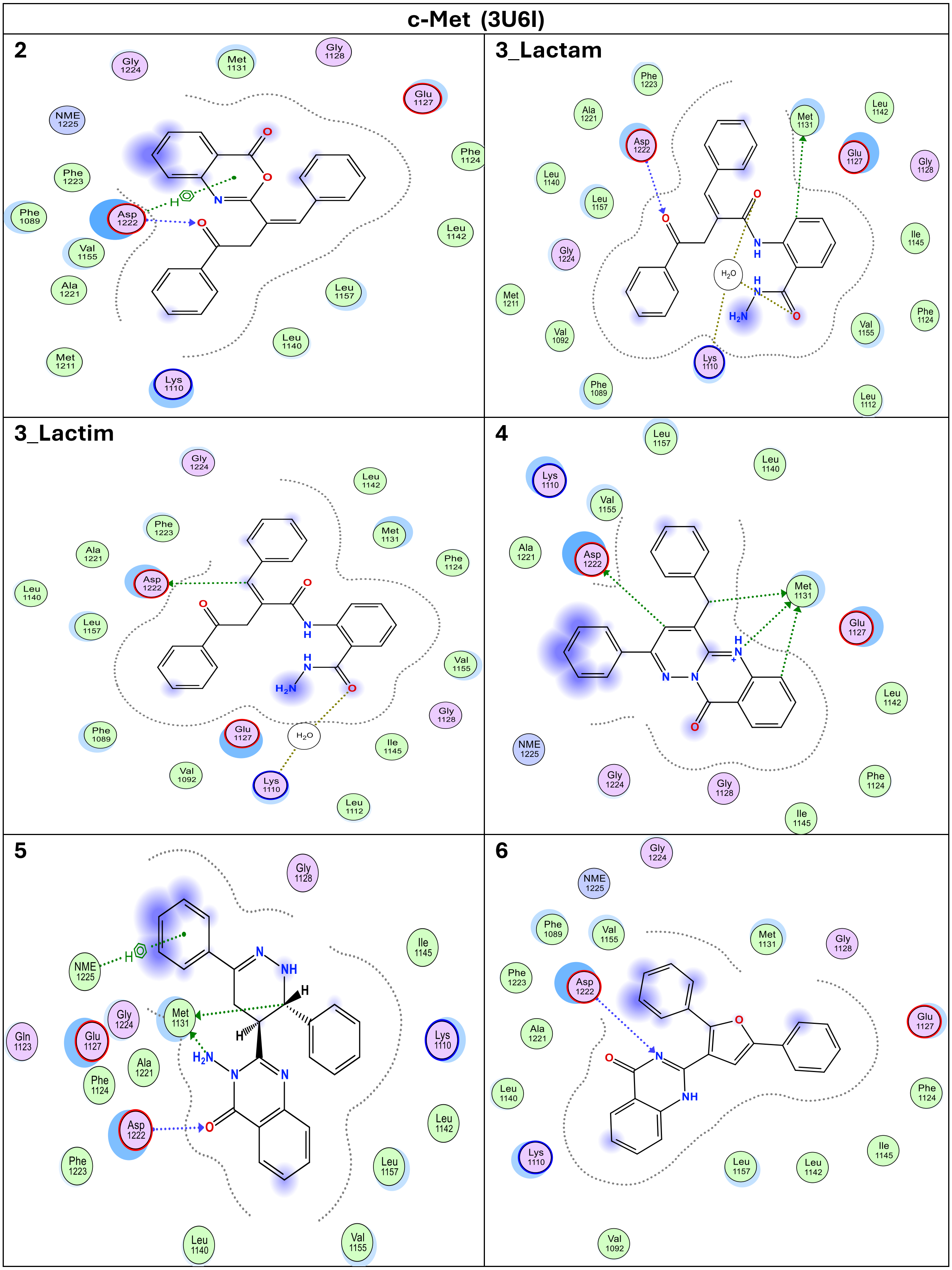

c-Met (3U6I)
2
Gly 1224
Met 1131
Gly 1128
Glu 1127
NME 1225
Phe 1223
Phe 1089
Asp 1222
Val 1155
Ala 1221
Met 1211
Lys 1110
Leu 1140
Leu 1157
Leu 1142
Phe 1124
3_Lactam
Phe 1223
Ala 1221
Asp 1222
Met 1131
Leu 1142
Glu 1127
Gly 1128
Leu 1140
Leu 1157
Gly 1224
Ile 1145
Met 1211
Val 1092
Lys 1110
Val 1155
Phe 1124
Phe 1089
Leu 1112
H$_2$O
3_Lactim
Gly 1224
Leu 1142
Phe 1223
Met 1131
Ala 1221
Phe 1124
Asp 1222
Leu 1140
Leu 1157
Val 1155
Gly 1128
Phe 1089
Glu 1127
H$_2$O
Ile 1145
Val 1092
Lys 1110
Leu 1112
4
Leu 1157
Leu 1140
Lys 1110
Val 1155
Ala 1221
Asp 1222
Met 1131
Glu 1127
Leu 1142
NME 1225
Gly 1224
Gly 1128
Phe 1124
Ile 1145
5
Gly 1128
Ile 1145
NME 1225
Gly 1224
Met 1131
Gln 1123
Glu 1127
Lys 1110
Ala 1221
Phe 1124
Asp 1222
Leu 1142
Phe 1223
Leu 1157
Leu 1140
Val 1155
6
Gly 1224
NME 1225
Phe 1089
Val 1155
Met 1131
Gly 1128
Phe 1223
Asp 1222
Glu 1127
Ala 1221
Phe 1124
Leu 1140
Lys 1110
Leu 1157
Leu 1142
Ile 1145
Val 1092

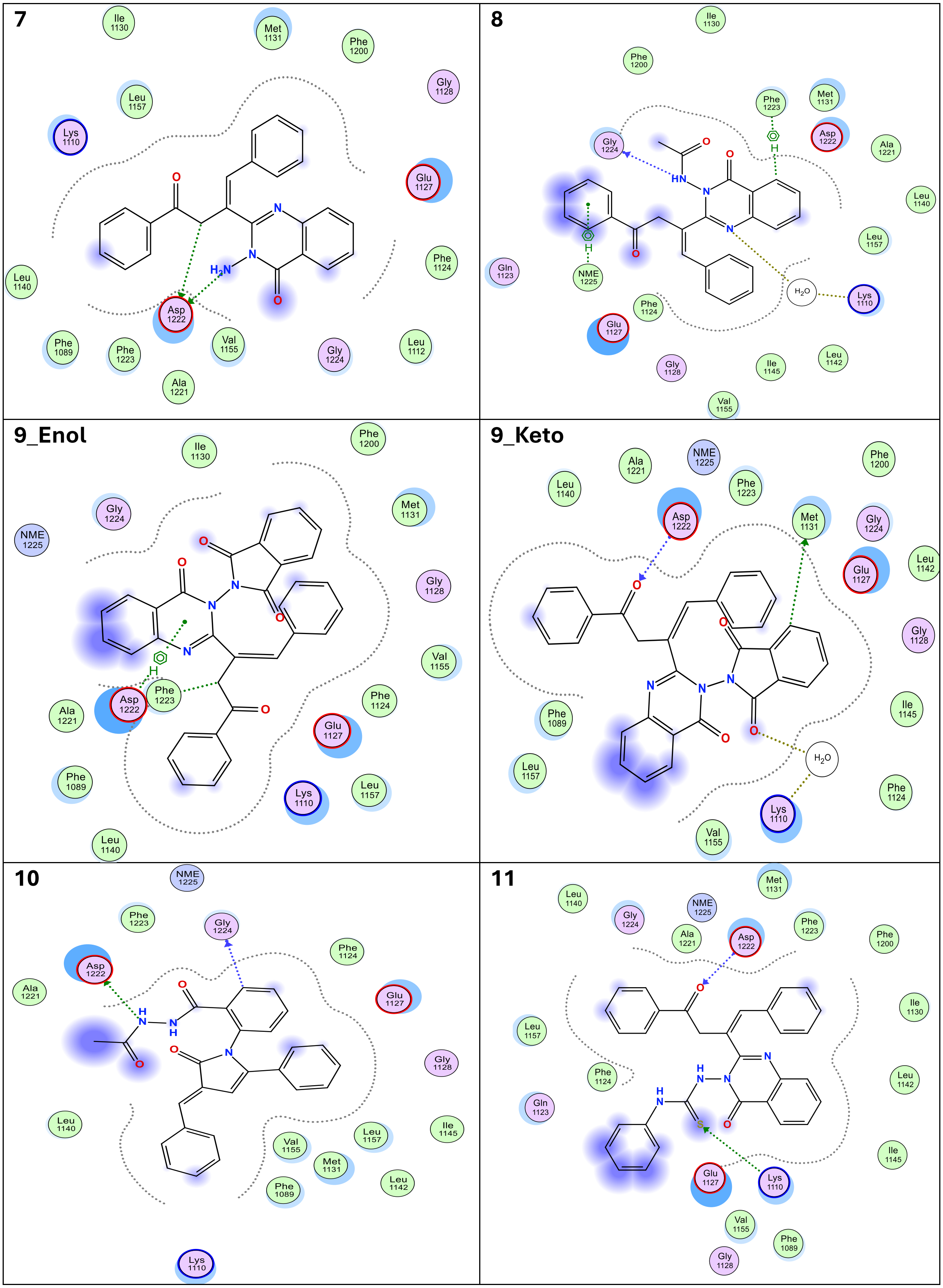

7
Ile 1130
Met 1131
Phe 1200
Gly 1128
Leu 1157
Lys 1110
Glu 1127
Phe 1124
Leu 1140
Asp 1222
Val 1155
Gly 1224
Leu 1112
Phe 1089
Phe 1223
Ala 1221
8
Ile 1130
Phe 1200
Phe 1223
Met 1131
Asp 1222
Ala 1221
Gly 1224
Leu 1140
Leu 1157
Gln 1123
NME 1225
H2O
Lys 1110
Phe 1124
Glu 1127
Leu 1142
Gly 1128
Ile 1145
Val 1155
9_Enol
Ile 1130
Phe 1200
Met 1131
Gly 1224
NME 1225
Gly 1128
Val 1155
Asp 1222
Phe 1223
Ala 1221
Phe 1124
Glu 1127
Phe 1089
Lys 1110
Leu 1157
Leu 1140
9_Keto
NME 1225
Ala 1221
Phe 1200
Leu 1140
Phe 1223
Asp 1222
Met 1131
Gly 1224
Glu 1127
Leu 1142
Gly 1128
Phe 1089
Ile 1145
Leu 1157
H2O
Lys 1110
Phe 1124
Val 1155
10
NME 1225
Phe 1223
Gly 1224
Asp 1222
Phe 1124
Ala 1221
Glu 1127
Gly 1128
Leu 1140
Ile 1145
Val 1155
Leu 1157
Met 1131
Phe 1089
Leu 1142
Lys 1110
11
Met 1131
Leu 1140
NME 1225
Gly 1224
Ala 1221
Asp 1222
Phe 1223
Phe 1200
Ile 1130
Leu 1157
Phe 1124
Leu 1142
Gln 1123
Ile 1145
Glu 1127
Lys 1110
Val 1155
Phe 1089
Gly 1128

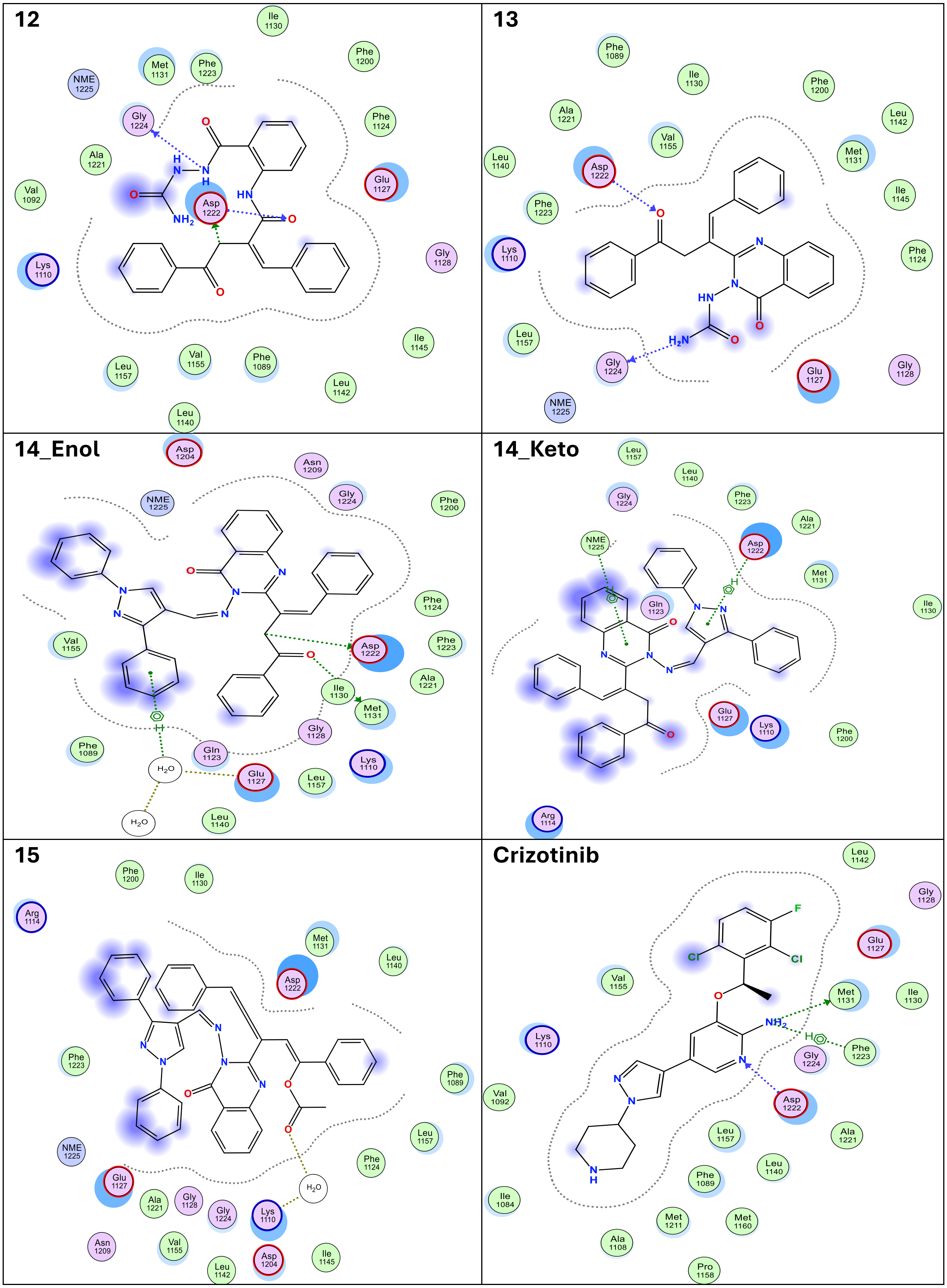

12
Ile 1130
Phe 1200
NME 1225
Met 1131
Phe 1223
Gly 1224
Phe 1124
Ala 1221
Glu 1127
Val 1092
Asp 1222
Lys 1110
Gly 1128
Ile 1145
Leu 1157
Val 1155
Phe 1089
Leu 1142
Leu 1140
13
Phe 1089
Ile 1130
Phe 1200
Ala 1221
Leu 1142
Val 1155
Met 1131
Leu 1140
Asp 1222
Phe 1223
Ile 1145
Lys 1110
Phe 1124
Leu 1157
Gly 1224
Glu 1127
Gly 1128
NME 1225
14_Enol
Asp 1204
Asn 1209
NME 1225
Gly 1224
Phe 1200
Phe 1124
Val 1155
Asp 1222
Phe 1223
Ala 1221
Ile 1130
Met 1131
Gly 1128
Phe 1089
Gln 1123
Glu 1127
Leu 1157
Lys 1110
Leu 1140
14_Keto
Leu 1157
Leu 1140
Gly 1224
Phe 1223
Ala 1221
NME 1225
Asp 1222
Met 1131
Ile 1130
Gln 1123
Glu 1127
Lys 1110
Phe 1200
Arg 1114
15
Phe 1200
Ile 1130
Arg 1114
Met 1131
Leu 1140
Asp 1222
Phe 1223
Phe 1089
Leu 1157
Phe 1124
NME 1225
Glu 1127
Ala 1221
Gly 1128
Gly 1224
Lys 1110
Asn 1209
Val 1155
Leu 1142
Asp 1204
Ile 1145
Crizotinib
Leu 1142
Gly 1128
Glu 1127
Val 1155
Met 1131
Ile 1130
Lys 1110
Phe 1223
Gly 1224
Val 1092
Asp 1222
Ala 1221
Leu 1157
Leu 1140
Phe 1089
Ile 1084
Met 1211
Met 1160
Ala 1108
Pro 1158

c-Met (3U6I)				
Ligand	Residues	Interaction Type	Distance (Å)	E (kcal/mol)
2	ASP 1222 (A)	H-acceptor	3.3	-1.2
	ASP 1222 (A)	pi-H	3.82	-1.2
3_Lactam	MET 1131 (A)	H-donor	3.69	-0.5
	HOH 93 (A)	H-acceptor	2.82	-0.7
	HOH 93 (A)	H-acceptor	2.71	-0.7
	ASP 1222 (A)	H-acceptor	3.44	-0.7
3_Lactim	ASP 1222 (A)	H-donor	3.3	-0.5
	HOH 93 (A)	H-acceptor	2.8	-0.8
4	MET 1131 (A)	H-donor	3.65	-0.6
	MET 1131 (A)	H-donor	3.49	-4.9
	ASP 1222 (A)	H-donor	3.36	-0.6
	MET 1131 (A)	H-donor	3.96	-0.5
5	ET 1131 (A)	H-donor	3.74	-0.8
	MET 1131 (A)	H-donor	3.61	-2.3
	ASP 1222 (A)	H-acceptor	2.95	-1.3
	NME 1225 (A)	pi-H	3.68	-0.6
6	ASP 1222 (A)	H-acceptor	3.4	-2
7	ASP 1222 (A)	H-donor	3.3	-1.7
	ASP 1222 (A)	H-donor	3.41	-0.6
8	GLY 1224 (A)	H-donor	3.23	-2.4
	HOH 93 (A)	H-acceptor	3.27	-0.5
	PHE 1223 (A)	H-pi	4.2	-0.7
	NME 1225 (A)	pi-H	3.99	-0.5
9_Enol	ASP 1222 (A)	H-donor	3.25	-0.6
	ASP 1222 (A)	pi-H	3.82	-1.4
9_Keto	MET 1131 (A)	H-donor	3.85	-0.6
	ASP 1222 (A)	H-acceptor	3.01	-1.4
	HOH 93 (A)	H-acceptor	2.89	-0.7
10	GLY 1224 (A)	H-donor	3.07	-0.5
	ASP 1222 (A)	H-donor	3.17	-3.9
11	ASP 1222 (A)	H-acceptor	3.05	-0.5
	LYS 1110 (A)	H-acceptor	4.46	-4
12	GLY 1224 (A)	H-donor	2.84	-1
	ASP 1222 (A)	H-donor	3.18	-0.5
	ASP 1222 (A)	H-acceptor	3.44	-0.7
13	GLY 1224 (A)	H-donor	3.39	-1.1
	ASP 1222 (A)	H-acceptor	3	-0.6
14_Enol	ASP 1222 (A)	H-donor	3.43	-0.6

	MET 1131 (A)	H-donor	3.81	-0.5
	HOH 77 (A)	pi-H	4.49	-0.5
14_Keto	ASP 1222 (A)	pi-H	4.6	-1.8
	NME 1225 (A)	pi-H	3.46	-0.6
15	HOH 93 (A)	H-acceptor	2.56	-0.9
Crizotinib	MET 1131 (A)	H-donor	3.12	-1
	ASP 1222 (A)	H-acceptor	2.88	-2.6
	PHE 1223 (A)	H-pi	4.27	-0.5

EGFR (1M17)

2

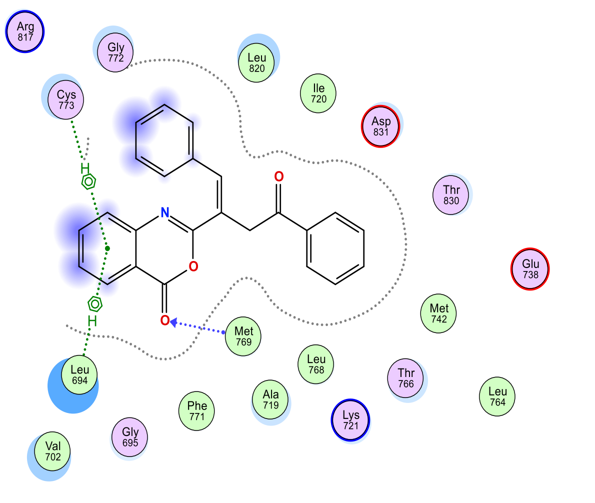

3_Lactam

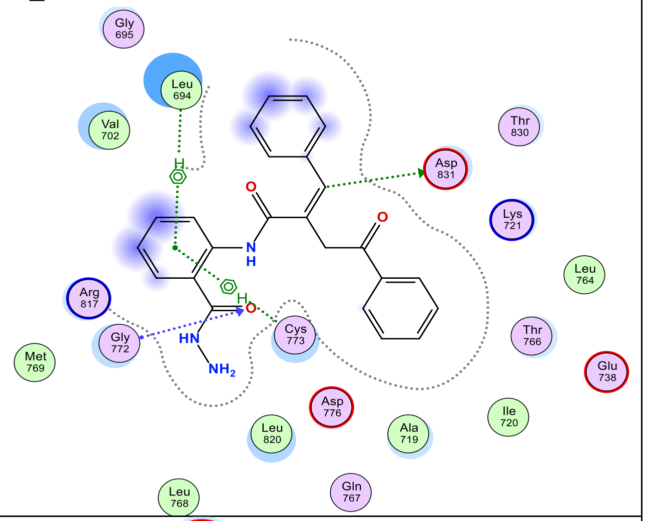

3_Lactim

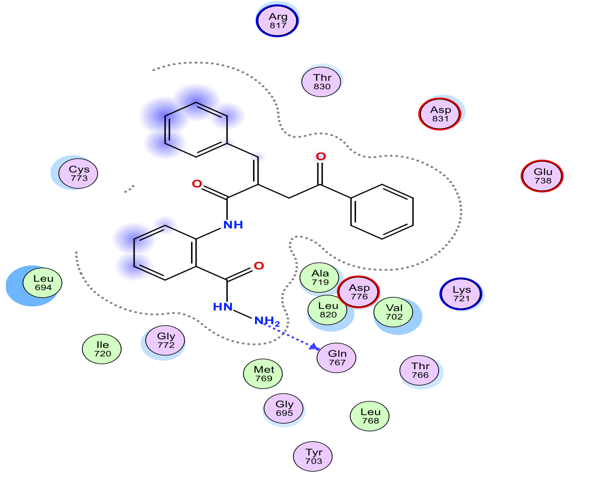

4

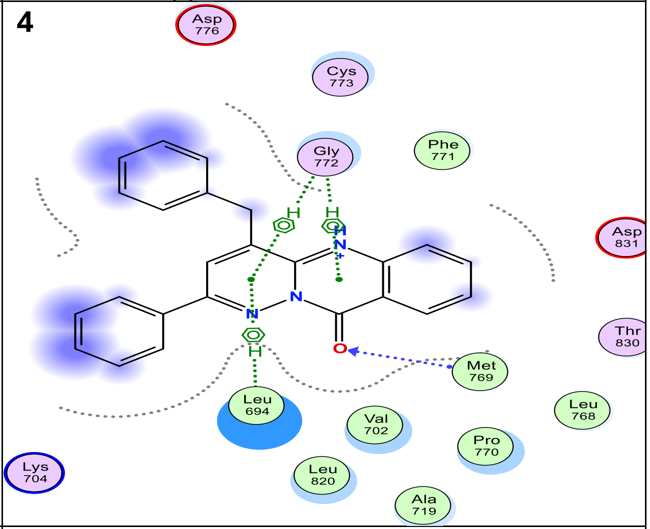

5

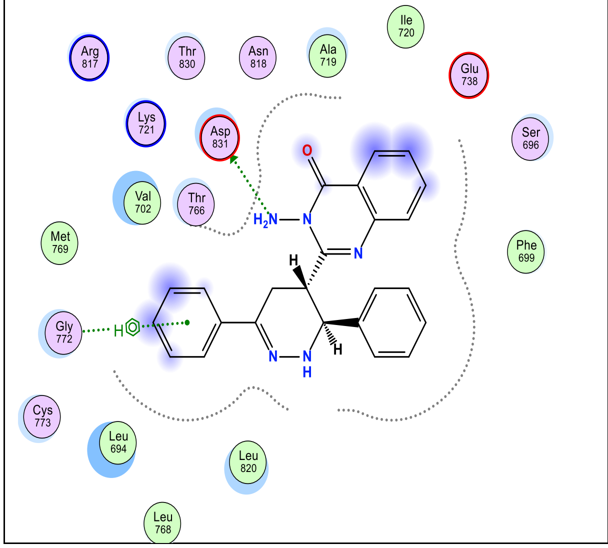

6

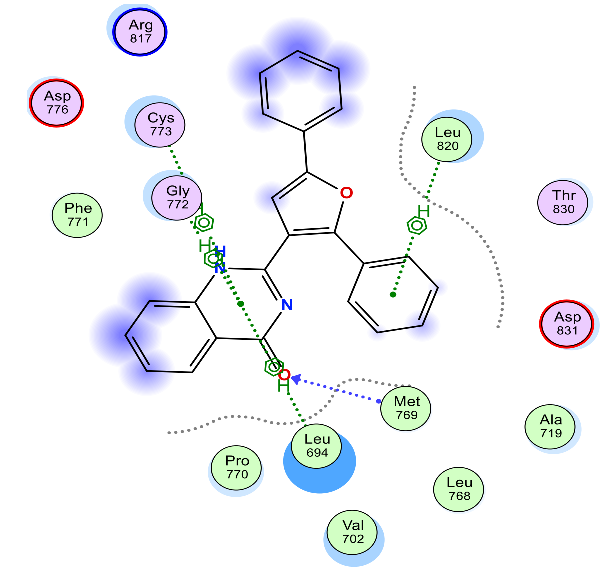

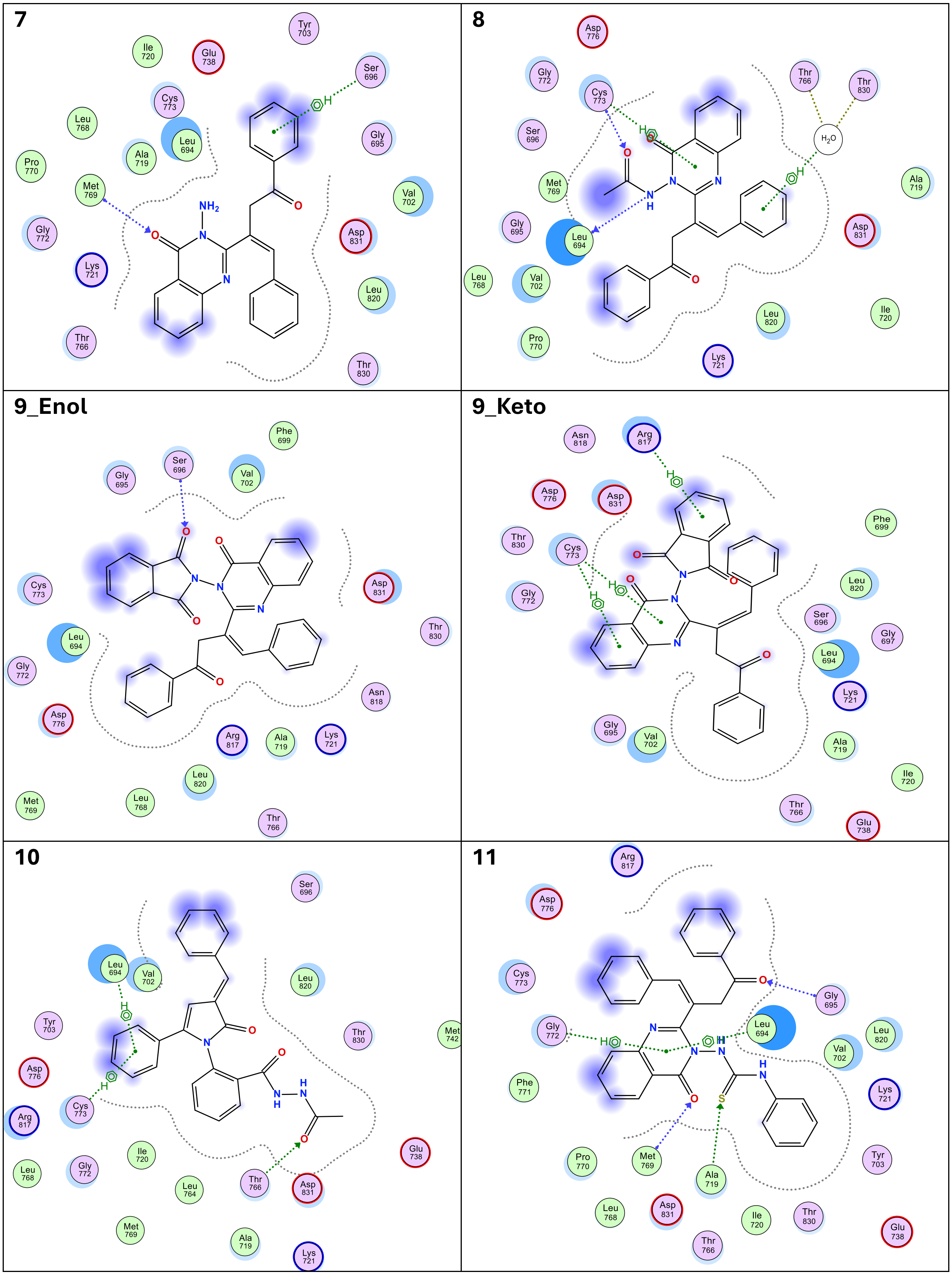
7
Ile 720
Glu 738
Tyr 703
Ser 696
Cys 773
Leu 768
Leu 694
Gly 695
Ala 719
Pro 770
Met 769
Val 702
Gly 772
Asp 831
Lys 721
Leu 820
Thr 766
Thr 830
8
Asp 776
Gly 772
Cys 773
Thr 766
Thr 830
Ser 696
H2O
Met 769
Ala 719
Gly 695
Leu 694
Asp 831
Leu 768
Val 702
Leu 820
Ile 720
Pro 770
Lys 721
9_Enol
Phe 699
Ser 696
Gly 695
Val 702
Cys 773
Asp 831
Leu 694
Thr 830
Gly 772
Asn 818
Asp 776
Arg 817
Ala 719
Lys 721
Leu 820
Met 769
Leu 768
Thr 766
9_Keto
Asn 818
Arg 817
Asp 776
Asp 831
Phe 699
Thr 830
Cys 773
Leu 820
Gly 772
Ser 696
Gly 697
Leu 694
Lys 721
Gly 695
Val 702
Ala 719
Ile 720
Thr 766
Glu 738
10
Ser 696
Leu 694
Val 702
Leu 820
Tyr 703
Thr 830
Met 742
Asp 776
Arg 817
Cys 773
Glu 738
Leu 768
Gly 772
Ile 720
Leu 764
Thr 766
Asp 831
Met 769
Ala 719
Lys 721
11
Arg 817
Asp 776
Cys 773
Gly 695
Gly 772
Leu 694
Leu 820
Val 702
Phe 771
Lys 721
Pro 770
Met 769
Ala 719
Tyr 703
Leu 768
Asp 831
Ile 720
Thr 830
Glu 738
Thr 766

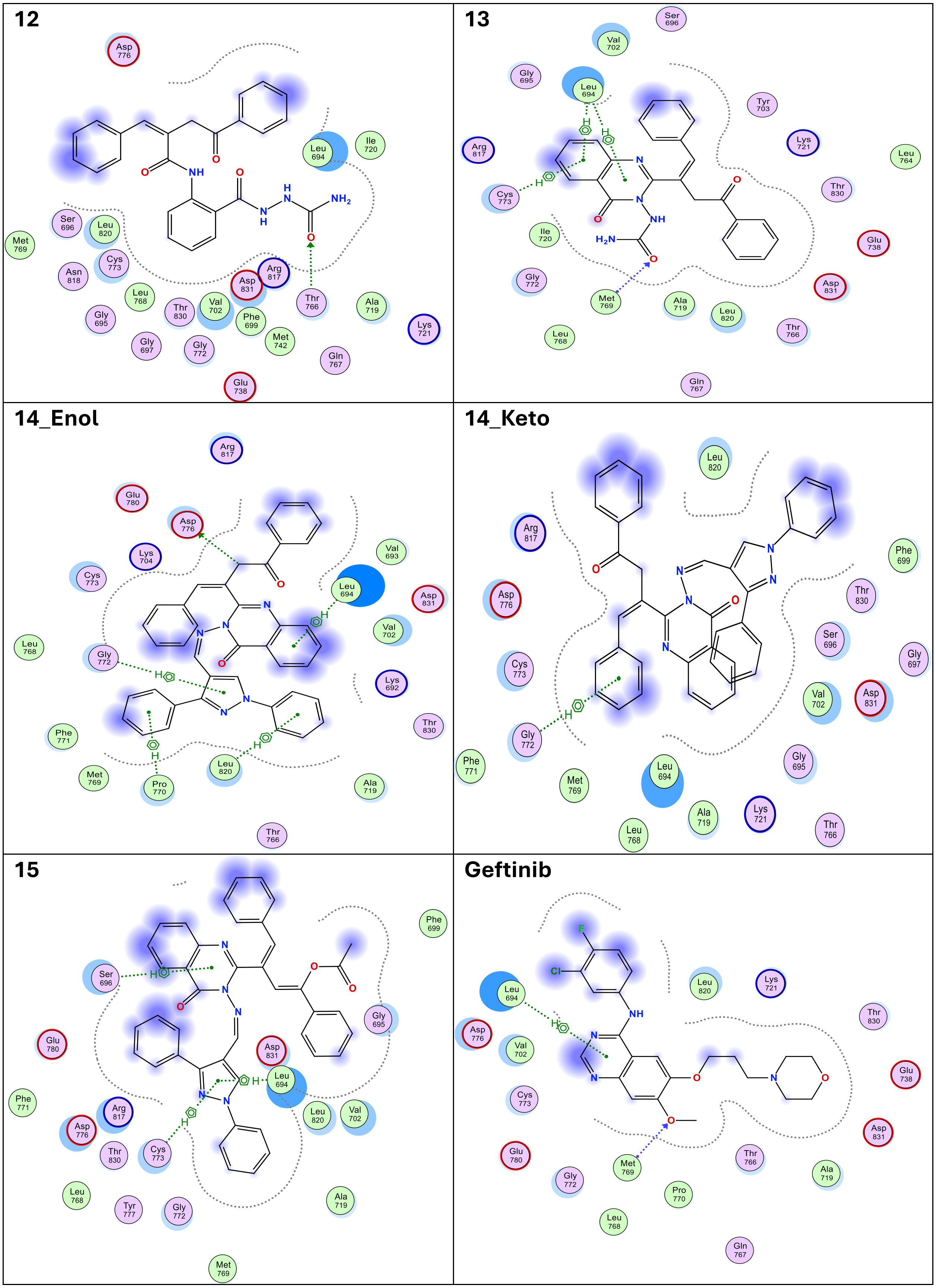
12
Asp 776
Leu 694
Ile 720
Ser 696
Leu 820
Met 769
Cys 773
Asn 818
Asp 831
Arg 817
Leu 768
Gly 695
Thr 830
Val 702
Phe 699
Thr 766
Ala 719
Lys 721
Gly 697
Gly 772
Met 742
Gln 767
Glu 738
13
Ser 696
Val 702
Gly 695
Leu 694
Tyr 703
Arg 817
Lys 721
Leu 764
Thr 830
Cys 773
Ile 720
Glu 738
Gly 772
Asp 831
Met 769
Ala 719
Leu 820
Thr 766
Leu 768
Gln 767
14_Enol
Arg 817
Glu 780
Asp 776
Lys 704
Cys 773
Val 693
Leu 694
Asp 831
Val 702
Leu 768
Gly 772
Lys 692
Thr 830
Phe 771
Met 769
Pro 770
Leu 820
Ala 719
Thr 766
14_Keto
Leu 820
Arg 817
Phe 699
Asp 776
Thr 830
Ser 696
Gly 697
Cys 773
Val 702
Asp 831
Gly 772
Phe 771
Met 769
Leu 694
Gly 695
Ala 719
Lys 721
Leu 768
Thr 766
15
Phe 699
Ser 696
Gly 695
Glu 780
Asp 831
Leu 694
Phe 771
Leu 820
Val 702
Arg 817
Asp 776
Thr 830
Cys 773
Leu 768
Tyr 777
Gly 772
Ala 719
Met 769
Geftinib
Leu 694
Leu 820
Lys 721
Thr 830
Asp 776
Val 702
Cys 773
Glu 738
Glu 780
Asp 831
Met 769
Thr 766
Gly 772
Ala 719
Pro 770
Leu 768
Gln 767

EGFR (1M17)				
Ligand	Residues	Interaction Type	Distance (Å)	E (kcal/mol)
2	MET 769 (A)	H-acceptor	3.09	-3.3
	LEU 694 (A)	pi-H	3.64	-0.6
	CYS 773 (A)	pi-H	4.31	-0.6
3_Lactam	ASP 831 (A)	H-donor	3.55	-0.6
	GLY 772 (A)	H-acceptor	3.42	-0.8
	LEU 694 (A)	pi-H	4.18	-0.8
	CYS 773 (A)	pi-H	4.02	-0.7
3_Lactim	GLN 767 (A)	H-donor	2.99	-1.7
4	MET 769 (A)	H-acceptor	3.39	-1.1
	LEU 694 (A)	pi-H	3.6	-0.5
	GLY 772 (A)	pi-H	3.35	-1.5
	GLY 772 (A)	pi-H	4.58	-1.4
5	ASP 831 (A)	H-donor	3.03	-3.8
	GLY 772 (A)	pi-H	3.39	-0.6
6	MET 769 (A)	H-acceptor	3.25	-2.3
	LEU 694 (A)	pi-H	3.65	-0.5
	GLY 772 (A)	pi-H	3.45	-0.6
	CYS 773 (A)	pi-H	4.42	-0.7
	LEU 820 (A)	pi-H	4.16	-0.6
7	MET 769 (A)	H-acceptor	3.47	-1.2
	SER 696 (A)	pi-H	4.34	-1
8	LEU 694 (A)	H-donor	2.84	-3
	CYS 773 (A)	H-acceptor	2.99	-3.9
	CYS 773 (A)	pi-H	4.59	-0.8
	HOH 10 (A)	pi-H	3.53	-0.5
9_Enol	SER 696 (A)	H-acceptor	2.94	-2.8
9_Keto	CYS 773 (A)	pi-H	3.79	-1.1
	CYS 773 (A)	pi-H	4	-0.5
	ARG 817 (A)	pi-H	3.87	-1.4
10	THR 766 (A)	H-acceptor	2.95	-0.7
	LEU 694 (A)	pi-H	4.04	-1.2
	CYS 773 (A)	pi-H	3.84	-0.5
11	MET 769 (A)	H-acceptor	3.37	-1.4
	GLY 695 (A)	H-acceptor	3.09	-0.6
	ALA 719 (A)	H-acceptor	3.58	-0.5
	LEU 694 (A)	pi-H	3.52	-0.5
	GLY 772 (A)	pi-H	3.4	-1.4
12	THR 766 (A)	H-acceptor	2.83	-0.8

**13**	**MET 769 (A)**	**H-acceptor**	**3.14**	**-0.9**
	**LEU 694 (A)**	**pi-H**	**4.24**	**-0.8**
	**LEU 694 (A)**	**pi-H**	**4.15**	**-0.8**
	**CYS 773 (A)**	**pi-H**	**3.92**	**-1.5**
**14_Enol**	**ASP 776 (A)**	**H-donor**	**3.16**	**-0.5**
	**LEU 694 (A)**	**pi-H**	**4.1**	**-0.6**
	**PRO 770 (A)**	**pi-H**	**3.99**	**-0.5**
	**GLY 772 (A)**	**pi-H**	**3.81**	**-0.6**
	**LEU 820 (A)**	**pi-H**	**3.68**	**-0.5**
**14_Keto**	**GLY 772 (A)**	**pi-H**	**3.8**	**-1.1**
**15**	**LEU 694 (A)**	**pi-H**	**4.58**	**-0.8**
	**SER 696 (A)**	**pi-H**	**4.5**	**-0.5**
	**CYS 773 (A)**	**pi-H**	**3.74**	**-3.3**
**Gefitinib**	**MET 769 (A)**	**H-acceptor**	**3.18**	**-0.8**
	**LEU 694 (A)**	**pi-H**	**3.93**	**-1.3**

ESTRα (3ERT)

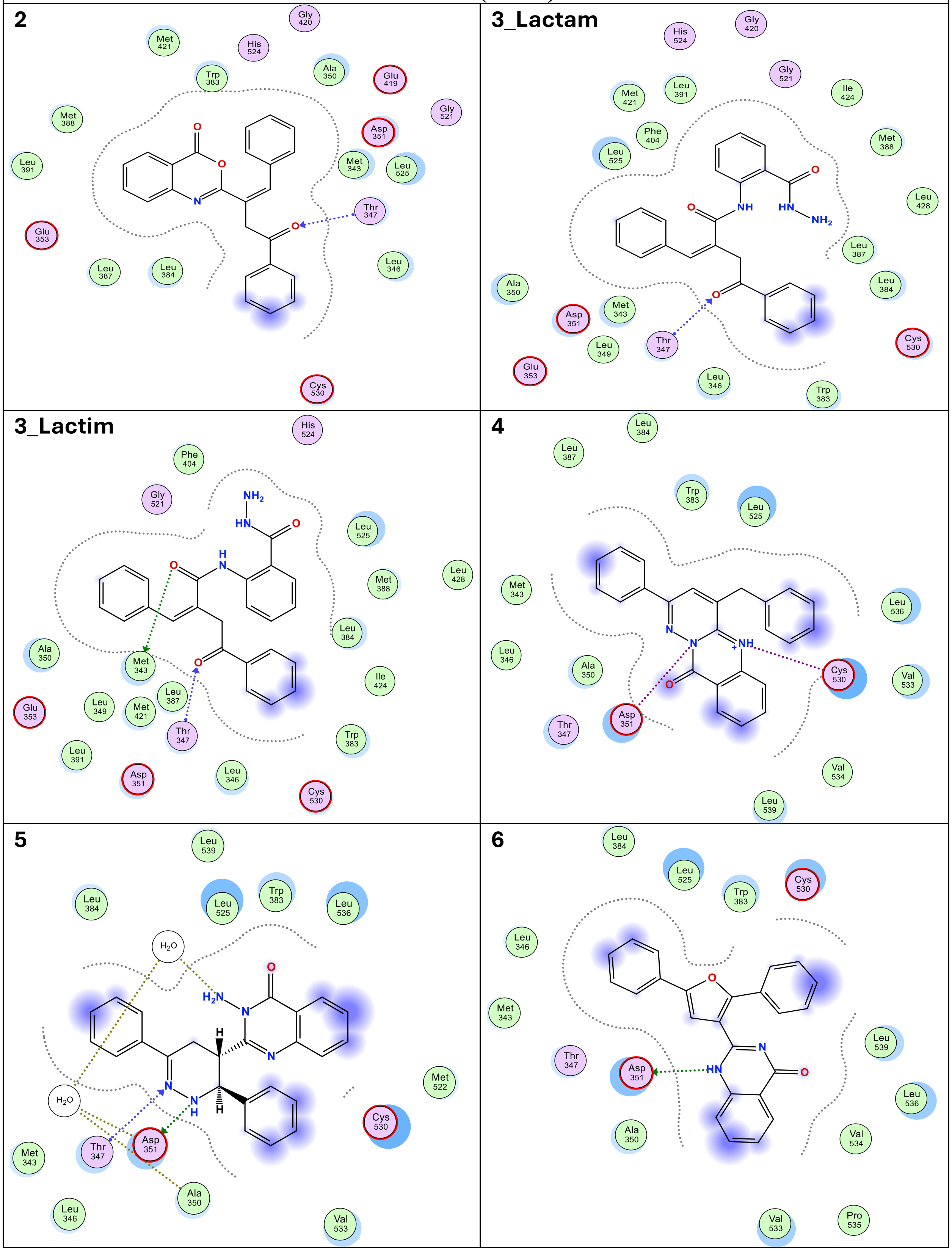
2
Gly 420
Met 421
His 524
Trp 383
Ala 350
Glu 419
Met 388
Gly 521
Asp 351
Leu 391
Met 343
Leu 525
Thr 347
Glu 353
Leu 387
Leu 384
Leu 346
Cys 530
3_Lactam
His 524
Gly 420
Gly 521
Met 421
Leu 391
Ile 424
Phe 404
Met 388
Leu 525
Leu 428
HN
NH
NH2
Leu 387
Leu 384
Ala 350
Met 343
Asp 351
Thr 347
Cys 530
Leu 349
Glu 353
Leu 346
Trp 383
3_Lactim
His 524
Phe 404
Gly 521
NH2
HN
Leu 525
Leu 428
Met 388
Leu 384
Ala 350
Met 343
Ile 424
Leu 387
Glu 353
Leu 349
Met 421
Thr 347
Trp 383
Leu 391
Asp 351
Leu 346
Cys 530
4
Leu 384
Leu 387
Trp 383
Leu 525
Met 343
Leu 536
Leu 346
Ala 350
NH
Cys 530
Val 533
Thr 347
Asp 351
Val 534
Leu 539
5
Leu 539
Leu 384
Trp 383
Leu 525
Leu 536
H2O
H2N
Met 522
Cys 530
Thr 347
Asp 351
Met 343
Ala 350
Leu 346
Val 533
6
Leu 384
Leu 525
Trp 383
Cys 530
Leu 346
Met 343
Leu 539
Thr 347
Asp 351
HN
Leu 536
Val 534
Ala 350
Val 533
Pro 535

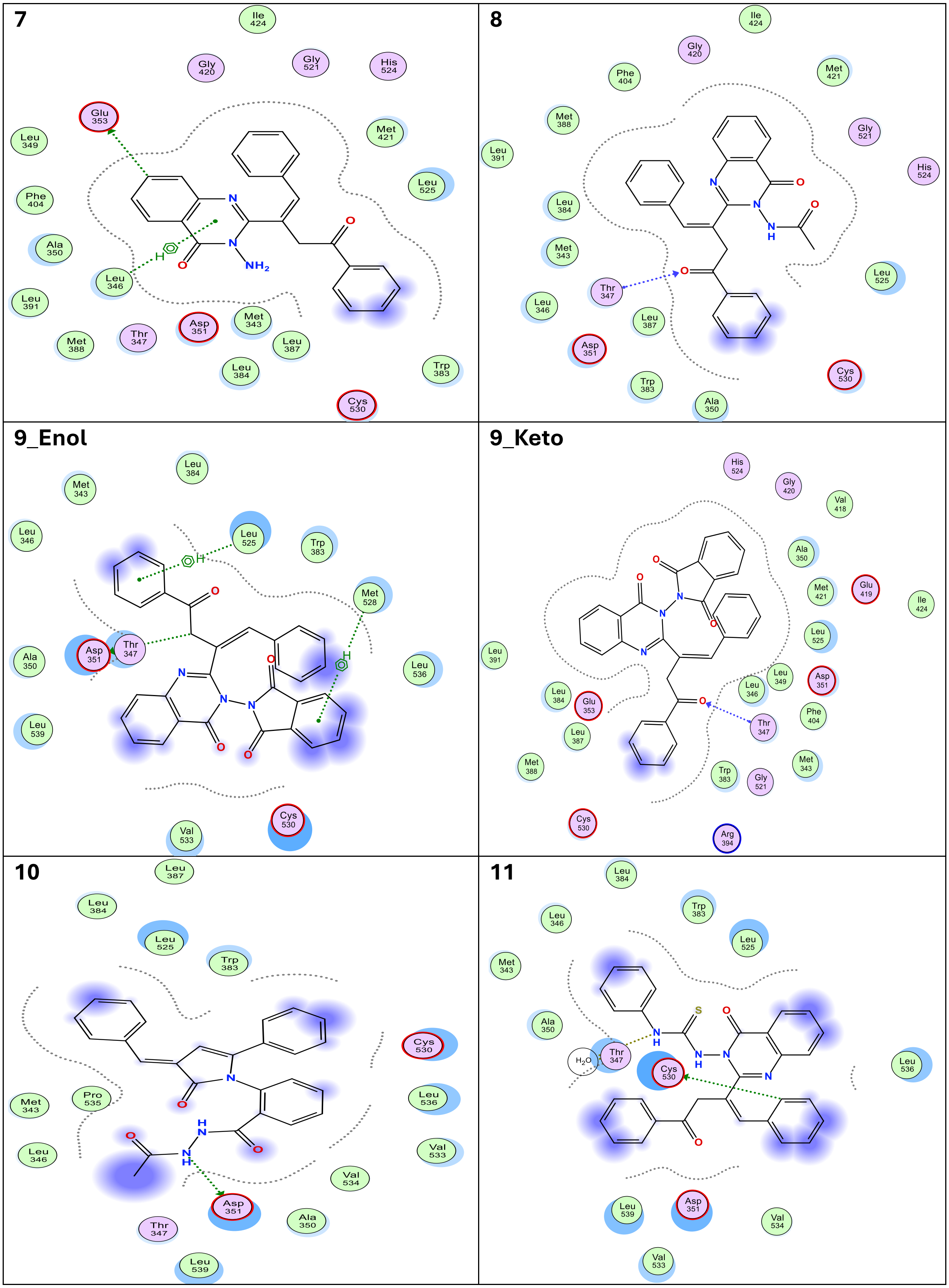
7
Ile 424
Gly 420
Gly 521
His 524
Glu 353
Leu 349
Met 421
Leu 525
Phe 404
N
O
Ala 350
H
N
NH2
O
Leu 346
Leu 391
Asp 351
Met 343
Leu 387
Met 388
Thr 347
Leu 384
Trp 383
Cys 530
8
Ile 424
Gly 420
Phe 404
Met 421
Met 388
Gly 521
Leu 391
His 524
N
O
Leu 384
N
O
N
H
Met 343
Leu 525
Thr 347
O
Leu 346
Leu 387
Asp 351
Cys 530
Trp 383
Ala 350
9_Enol
Leu 384
Met 343
Leu 346
Leu 525
Trp 383
H
O
Met 528
Ala 350
Asp 351
Thr 347
H
Leu 536
N
N
N
O
Leu 539
O
O
Cys 530
Val 533
9_Keto
His 524
Gly 420
Val 418
Ala 350
O
Glu 419
Met 421
Ile 424
O
N
N
O
Leu 525
Leu 391
N
Leu 349
Asp 351
Leu 346
Leu 384
Glu 353
O
Phe 404
Thr 347
Leu 387
Met 388
Met 343
Trp 383
Gly 521
Cys 530
Arg 394
10
Leu 387
Leu 384
Leu 525
Trp 383
Cys 530
N
O
Leu 536
Met 343
Pro 535
H
N
O
O
Leu 346
N
H
Val 533
Val 534
Asp 351
Ala 350
Thr 347
Leu 539
11
Leu 384
Trp 383
Leu 346
Leu 525
Met 343
S
O
Ala 350
N
H
Thr 347
N
H
N
Leu 536
H2O
Cys 530
N
O
Leu 539
Asp 351
Val 534
Val 533

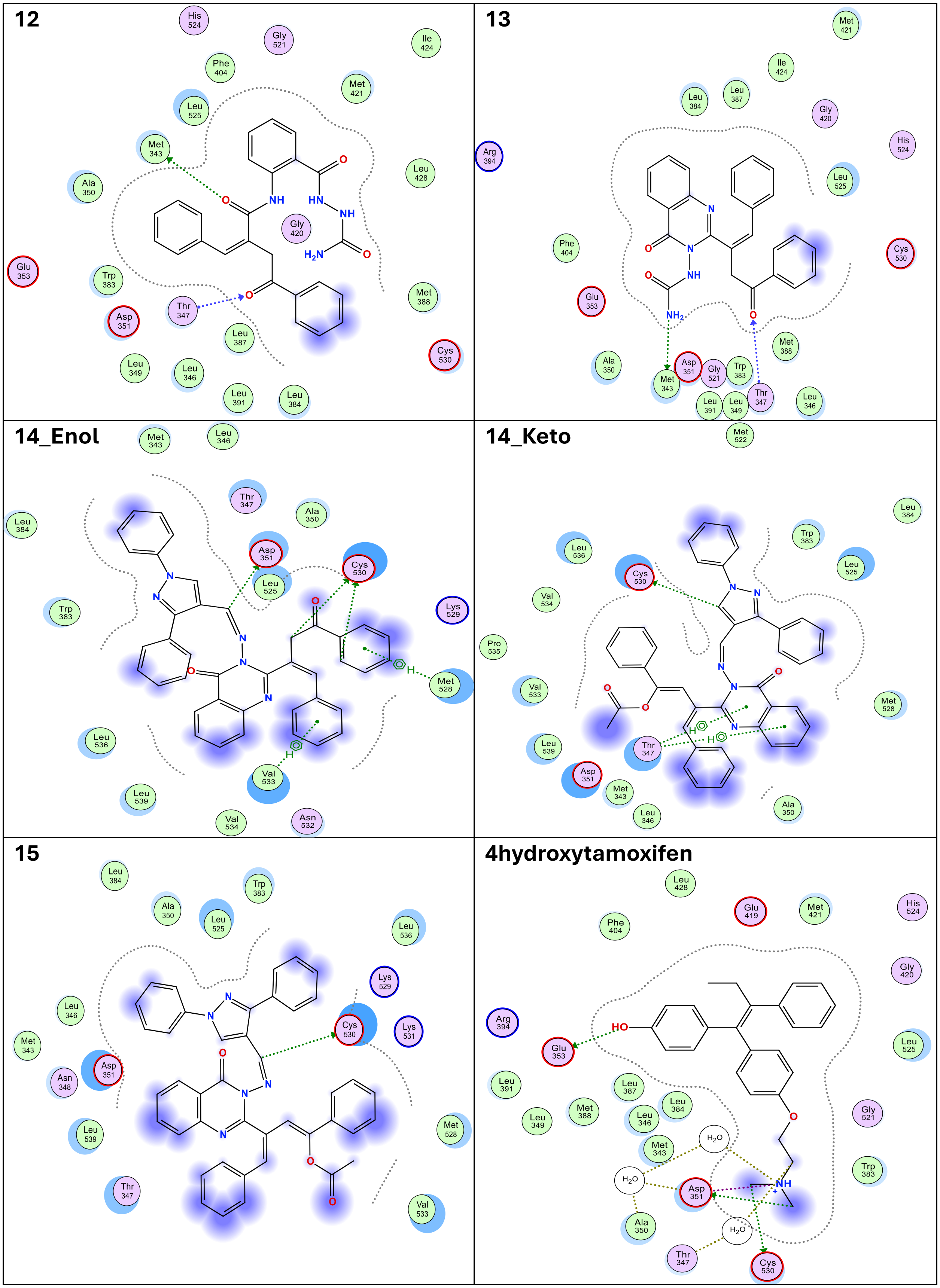

12
His 524
Gly 521
Ile 424
Phe 404
Met 421
Leu 525
Met 343
Ala 350
Leu 428
Gly 420
Glu 353
Trp 383
Met 388
Asp 351
Thr 347
Leu 387
Cys 530
Leu 349
Leu 346
Leu 391
Leu 384
13
Met 421
Ile 424
Leu 384
Leu 387
Gly 420
Arg 394
His 524
Leu 525
Phe 404
Cys 530
Glu 353
Met 388
Ala 350
Asp 351
Gly 521
Trp 383
Met 343
Thr 347
Leu 391
Leu 349
Leu 346
14_Enol
Met 343
Leu 346
Leu 384
Thr 347
Ala 350
Asp 351
Cys 530
Leu 525
Trp 383
Lys 529
Met 528
Leu 536
Val 533
Leu 539
Val 534
Asn 532
14_Keto
Met 522
Leu 384
Trp 383
Leu 536
Leu 525
Cys 530
Val 534
Pro 535
Val 533
Met 528
Leu 539
Thr 347
Asp 351
Met 343
Leu 346
Ala 350
15
Leu 384
Trp 383
Ala 350
Leu 525
Leu 536
Lys 529
Leu 346
Cys 530
Lys 531
Met 343
Asn 348
Asp 351
Met 528
Leu 539
Thr 347
Val 533
4hydroxytamoxifen
Leu 428
Glu 419
Met 421
His 524
Phe 404
Gly 420
Arg 394
Glu 353
Leu 525
Leu 391
Leu 387
Leu 384
Leu 349
Met 388
Leu 346
Gly 521
Met 343
Trp 383
Asp 351
Ala 350
Thr 347
Cys 530

ESTRα (3ERT)				
Compound	Residues	Interaction	Distance	E (kcal/mol)
2	THR 347 (A)	H-acceptor	3.34	-0.6
3_Lactam	THR 347 (A)	H-acceptor	3.26	-1
3_Lactim	MET 343 (A)	H-donor	3.26	-0.9
	THR 347 (A)	H-acceptor	3.28	-1
4	CYS 530 (A)	Ionic	3.56	-1.7
	ASP 351 (A)	Ionic	3.46	-2.1
	ASP 351 (A)	Ionic	3.89	-0.7
5	ASP 351 (A)	H-donor	3.34	-1.5
	HOH 58 (A)	H-donor	3.03	-1.9
	THR 347 (A)	H-acceptor	3.6	-0.5
6	ASP 351 (A)	H-donor	3.32	-0.6
7	GLU 353 (A)	H-donor	3.35	-0.5
	LEU 346 (A)	pi-H	4.47	-0.6
8	THR 347 (A)	H-acceptor	3.41	-0.7
9_Enol	ASP 351 (A)	H-donor	3.47	-0.6
	LEU 525 (A)	pi-H	4.31	-0.6
	MET 528 (A)	pi-H	4.72	-0.5
9_Keto	THR 347 (A)	H-acceptor	3.37	-0.6
10	ASP 351 (A)	H-donor	3.01	-1.5
11	HOH 31 (A)	H-donor	2.75	-0.8
	CYS 530 (A)	H-donor	3.31	-0.5
12	MET 343 (A)	H-donor	3.44	-0.7
	THR 347 (A)	H-acceptor	3.27	-1.2
13	MET 343 (A)	H-donor	3.09	-2.6
	THR 347 (A)	H-acceptor	3.45	-0.5
14_Enol	ASP 351 (A)	H-donor	3.13	-0.7
	CYS 530 (A)	H-donor	4.09	-1.7
	CYS 530 (A)	H-donor	4.12	-1.5
	MET 528 (A)	pi-H	4.12	-0.6
	VAL 533 (A)	pi-H	4.19	-0.9
14_Keto	CYS 530 (A)	H-donor	3.59	-1.4
	THR 347 (A)	pi-H	4.62	-0.7
	THR 347 (A)	pi-H	3.6	-0.7
15	CYS 530 (A)	H-donor	3.45	-2
4hydroxytamoxifen	GLU 353 (A)	H-donor	2.92	-3.1
	HOH 31 (A)	H-donor	3.18	-0.7
	HOH 58 (A)	H-donor	3.2	-1.8
	CYS 530 (A)	H-donor	3.61	-2.1

	**ASP 351 (A)**	**H-donor**	**3.54**	**-0.6**
	**ASP 351 (A)**	**Ionic**	**3.28**	**-2.8**

Molecule 2

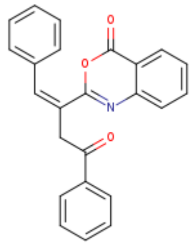

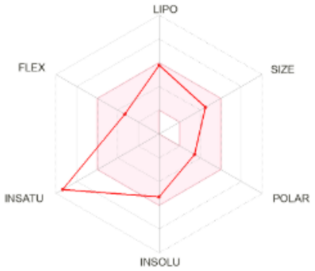

SMILES O=C(c1ccccc1)C/C(=C/c1ccccc1)/c1nc2ccccc2c(=O)o1

Physicochemical Properties	
Formula	C24H17NO3
Molecular weight	367.40 g/mol
Num. heavy atoms	28
Num. arom. heavy atoms	22
Fraction Csp3	0.04
Num. rotatable bonds	5
Num. H-bond acceptors	4
Num. H-bond donors	0
Molar Refractivity	110.17
TPSA	60.17 Å²

Lipophilicity	
Log $P_{o/w}$ (iLOGP)	3.30
Log $P_{o/w}$ (XLOGP3)	4.64
Log $P_{o/w}$ (WLOGP)	4.89
Log $P_{o/w}$ (MLOGP)	3.33
Log $P_{o/w}$ (SILICOS-IT)	5.58
Consensus Log $P_{o/w}$	4.35

Water Solubility	
Log *S* (ESOL)	-5.29
Solubility	1.87e-03 mg/ml ; 5.10e-06 mol/l
Class	Moderately soluble
Log *S* (Ali)	-5.63
Solubility	8.62e-04 mg/ml ; 2.35e-06 mol/l
Class	Moderately soluble
Log *S* (SILICOS-IT)	-8.66
Solubility	7.96e-07 mg/ml ; 2.17e-09 mol/l
Class	Poorly soluble

Pharmacokinetics	
GI absorption	High
BBB permeant	Yes
P-gp substrate	No
CYP1A2 inhibitor	Yes
CYP2C19 inhibitor	Yes
CYP2C9 inhibitor	Yes
CYP2D6 inhibitor	No
CYP3A4 inhibitor	Yes
Log K_p (skin permeation)	-5.25 cm/s

Druglikeness	
Lipinski	Yes; 0 violation
Ghose	Yes
Veber	Yes
Egan	Yes
Muegge	Yes
Bioavailability Score	0.55

Medicinal Chemistry	
PAINS	0 alert
Brenk	0 alert
Leadlikeness	No; 2 violations: MW>350, XLOGP3>3.5
Synthetic accessibility	3.53

Molecule 3

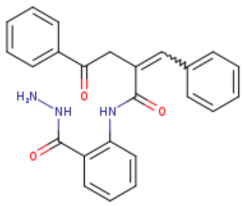

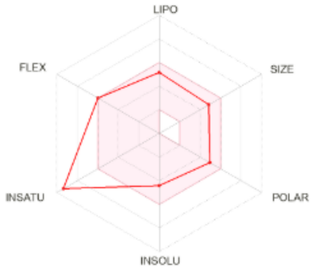

SMILES NNC(=O)c1ccccc1NC(=O)/C(=C\c1ccccc1)/CC(=O)c1ccccc1

Physicochemical Properties	
Formula	C24H21N3O3
Molecular weight	399.44 g/mol
Num. heavy atoms	30
Num. arom. heavy atoms	18
Fraction Csp3	0.04
Num. rotatable bonds	9
Num. H-bond acceptors	4
Num. H-bond donors	3
Molar Refractivity	115.79
TPSA	101.29 Å²

Lipophilicity	
Log $P_{o/w}$ (iLOGP)	2.23
Log $P_{o/w}$ (XLOGP3)	3.55
Log $P_{o/w}$ (WLOGP)	3.29
Log $P_{o/w}$ (MLOGP)	2.99
Log $P_{o/w}$ (SILICOS-IT)	3.26
Consensus Log $P_{o/w}$	3.06

Water Solubility	
Log *S* (ESOL)	-4.40
Solubility	1.58e-02 mg/ml ; 3.95e-05 mol/l
Class	Moderately soluble
Log *S* (Ali)	-5.36
Solubility	1.74e-03 mg/ml ; 4.34e-06 mol/l
Class	Moderately soluble
Log *S* (SILICOS-IT)	-7.44
Solubility	1.45e-05 mg/ml ; 3.64e-08 mol/l
Class	Poorly soluble

Pharmacokinetics	
GI absorption	High
BBB permeant	No
P-gp substrate	No
CYP1A2 inhibitor	No
CYP2C19 inhibitor	Yes
CYP2C9 inhibitor	Yes
CYP2D6 inhibitor	No
CYP3A4 inhibitor	Yes
Log K_p (skin permeation)	-6.22 cm/s

Druglikeness	
Lipinski	Yes; 0 violation
Ghose	Yes
Veber	Yes
Egan	Yes
Muegge	Yes
Bioavailability Score	0.55

Medicinal Chemistry	
PAINS	0 alert
Brenk	3 alerts: acyl_hydrazine, hydrazine, michael_acceptor_1
Leadlikeness	No; 3 violations: MW>350, Rotors>7, XLOGP3>3.5
Synthetic accessibility	3.13

Molecule 4

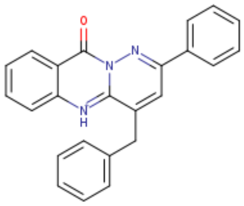

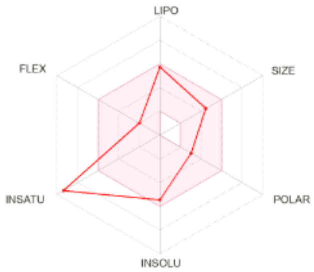

SMILES O=c1n2nc(cc(c2[nH+]c2c1cccc2)Cc1ccccc1)c1ccccc1

Physicochemical Properties	
Formula	C24H18N3O+
Molecular weight	364.42 g/mol
Num. heavy atoms	28
Num. arom. heavy atoms	26
Fraction Csp3	0.04
Num. rotatable bonds	3
Num. H-bond acceptors	2
Num. H-bond donors	1
Molar Refractivity	112.55
TPSA	48.51 Å²

Lipophilicity	
Log $P_{o/w}$ (iLOGP)	3.31
Log $P_{o/w}$ (XLOGP3)	4.52
Log $P_{o/w}$ (WLOGP)	3.92
Log $P_{o/w}$ (MLOGP)	5.05
Log $P_{o/w}$ (SILICOS-IT)	4.84
Consensus Log $P_{o/w}$	4.33

Water Solubility	
Log *S* (ESOL)	-5.44
Solubility	1.33e-03 mg/ml ; 3.66e-06 mol/l
Class	Moderately soluble
Log *S* (Ali)	-5.26
Solubility	2.00e-03 mg/ml ; 5.49e-06 mol/l
Class	Moderately soluble
Log *S* (SILICOS-IT)	-9.10
Solubility	2.87e-07 mg/ml ; 7.89e-10 mol/l
Class	Poorly soluble

Pharmacokinetics	
GI absorption	High
BBB permeant	Yes
P-gp substrate	Yes
CYP1A2 inhibitor	Yes
CYP2C19 inhibitor	Yes
CYP2C9 inhibitor	Yes
CYP2D6 inhibitor	No
CYP3A4 inhibitor	No
Log K_p (skin permeation)	-5.31 cm/s

Druglikeness	
Lipinski	Yes; 1 violation: MLOGP>4.15
Ghose	Yes
Veber	Yes
Egan	Yes
Muegge	Yes
Bioavailability Score	0.55

Medicinal Chemistry	
PAINS	0 alert
Brenk	1 alert: polycyclic_aromatic_hydrocarbon_2
Leadlikeness	No; 2 violations: MW>350, XLOGP3>3.5
Synthetic accessibility	3.15

Molecule 5

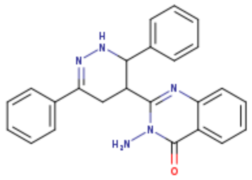

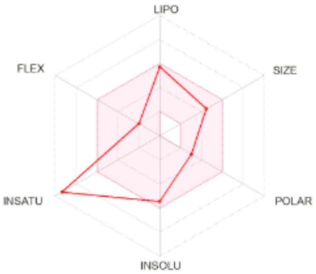

SMILES O=c1n2nc(cc(c2[nH+]c2c1cccc2)Cc1ccccc1)c1ccccc1

Physicochemical Properties	
Formula	C24H18N3O+
Molecular weight	364.42 g/mol
Num. heavy atoms	28
Num. arom. heavy atoms	26
Fraction Csp3	0.04
Num. rotatable bonds	3
Num. H-bond acceptors	2
Num. H-bond donors	1
Molar Refractivity	112.55
TPSA	48.51 Å²

Lipophilicity	
Log $P_{o/w}$ (iLOGP)	3.31
Log $P_{o/w}$ (XLOGP3)	4.52
Log $P_{o/w}$ (WLOGP)	3.92
Log $P_{o/w}$ (MLOGP)	5.05
Log $P_{o/w}$ (SILICOS-IT)	4.84
Consensus Log $P_{o/w}$	4.33

Water Solubility	
Log *S* (ESOL)	-5.44
Solubility	1.33e-03 mg/ml ; 3.66e-06 mol/l
Class	Moderately soluble
Log *S* (Ali)	-5.26
Solubility	2.00e-03 mg/ml ; 5.49e-06 mol/l
Class	Moderately soluble
Log *S* (SILICOS-IT)	-9.10
Solubility	2.87e-07 mg/ml ; 7.89e-10 mol/l
Class	Poorly soluble

Pharmacokinetics	
GI absorption	High
BBB permeant	Yes
P-gp substrate	Yes
CYP1A2 inhibitor	Yes
CYP2C19 inhibitor	Yes
CYP2C9 inhibitor	Yes
CYP2D6 inhibitor	No
CYP3A4 inhibitor	No
Log K_p (skin permeation)	-5.31 cm/s

Druglikeness	
Lipinski	Yes; 1 violation: MLOGP>4.15
Ghose	Yes
Veber	Yes
Egan	Yes
Muegge	Yes
Bioavailability Score	0.55

Medicinal Chemistry	
PAINS	0 alert
Brenk	1 alert: polycyclic_aromatic_hydrocarbon_2
Leadlikeness	No; 2 violations: MW>350, XLOGP3>3.5
Synthetic accessibility	3.15

Molecule 6

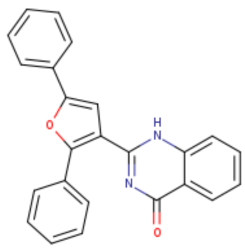

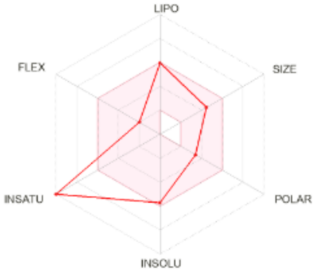

SMILES O=c1nc([nH]c2c1cccc2)c1cc(oc1c1ccccc1)c1ccccc1

Physicochemical Properties	
Formula	C24H16N2O2
Molecular weight	364.40 g/mol
Num. heavy atoms	28
Num. arom. heavy atoms	27
Fraction Csp3	0.00
Num. rotatable bonds	3
Num. H-bond acceptors	3
Num. H-bond donors	1
Molar Refractivity	110.94
TPSA	58.89 Å²

Lipophilicity	
Log $P_{o/w}$ (iLOGP)	3.13
Log $P_{o/w}$ (XLOGP3)	4.92
Log $P_{o/w}$ (WLOGP)	5.52
Log $P_{o/w}$ (MLOGP)	3.97
Log $P_{o/w}$ (SILICOS-IT)	6.03
Consensus Log $P_{o/w}$	4.71

Water Solubility	
Log *S* (ESOL)	-5.71
Solubility	7.03e-04 mg/ml ; 1.93e-06 mol/l
Class	Moderately soluble
Log *S* (Ali)	-5.89
Solubility	4.66e-04 mg/ml ; 1.28e-06 mol/l
Class	Moderately soluble
Log *S* (SILICOS-IT)	-9.95
Solubility	4.09e-08 mg/ml ; 1.12e-10 mol/l
Class	Poorly soluble

Pharmacokinetics	
GI absorption	High
BBB permeant	No
P-gp substrate	Yes
CYP1A2 inhibitor	Yes
CYP2C19 inhibitor	Yes
CYP2C9 inhibitor	No
CYP2D6 inhibitor	No
CYP3A4 inhibitor	No
Log K_p (skin permeation)	-5.03 cm/s

Druglikeness	
Lipinski	Yes; 0 violation
Ghose	Yes
Veber	Yes
Egan	Yes
Muegge	Yes
Bioavailability Score	0.55

Medicinal Chemistry	
PAINS	0 alert
Brenk	0 alert
Leadlikeness	No; 2 violations: MW>350, XLOGP3>3.5
Synthetic accessibility	3.49

Molecule 7

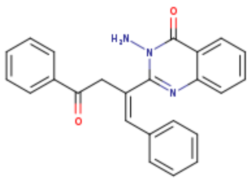

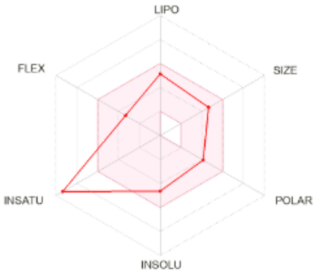

SMILES O=C(c1ccccc1)C/C(=C/c1ccccc1)/c1nc2ccccc2c(=O)n1N

Physicochemical Properties	
Formula	C24H19N3O2
Molecular weight	381.43 g/mol
Num. heavy atoms	29
Num. arom. heavy atoms	22
Fraction Csp3	0.04
Num. rotatable bonds	5
Num. H-bond acceptors	3
Num. H-bond donors	1
Molar Refractivity	116.04
TPSA	77.98 Å²

Lipophilicity	
Log $P_{o/w}$ (iLOGP)	3.11
Log $P_{o/w}$ (XLOGP3)	3.50
Log $P_{o/w}$ (WLOGP)	3.82
Log $P_{o/w}$ (MLOGP)	3.58
Log $P_{o/w}$ (SILICOS-IT)	3.94
Consensus Log $P_{o/w}$	3.59

Water Solubility	
Log *S* (ESOL)	-4.64
Solubility	8.71e-03 mg/ml ; 2.28e-05 mol/l
Class	Moderately soluble
Log *S* (Ali)	-4.82
Solubility	5.76e-03 mg/ml ; 1.51e-05 mol/l
Class	Moderately soluble
Log *S* (SILICOS-IT)	-7.47
Solubility	1.30e-05 mg/ml ; 3.42e-08 mol/l
Class	Poorly soluble

Pharmacokinetics	
GI absorption	High
BBB permeant	No
P-gp substrate	No
CYP1A2 inhibitor	No
CYP2C19 inhibitor	Yes
CYP2C9 inhibitor	Yes
CYP2D6 inhibitor	No
CYP3A4 inhibitor	No
Log K_p (skin permeation)	-6.14 cm/s

Druglikeness	
Lipinski	Yes; 0 violation
Ghose	Yes
Veber	Yes
Egan	Yes
Muegge	Yes
Bioavailability Score	0.55

Medicinal Chemistry	
PAINS	0 alert
Brenk	0 alert
Leadlikeness	No; 1 violation: MW>350
Synthetic accessibility	3.35

Molecule 8

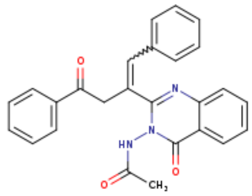

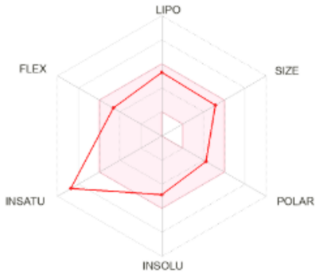

SMILES CC(=O)Nn1c(nc2c(c1=O)cccc2)/C(=C\c1ccccc1)/CC(=O)c1ccccc1

Physicochemical Properties	
Formula	C26H21N3O3
Molecular weight	423.46 g/mol
Num. heavy atoms	32
Num. arom. heavy atoms	22
Fraction Csp3	0.08
Num. rotatable bonds	7
Num. H-bond acceptors	4
Num. H-bond donors	1
Molar Refractivity	125.95
TPSA	81.06 Å²

Lipophilicity	
Log $P_{o/w}$ (iLOGP)	3.15
Log $P_{o/w}$ (XLOGP3)	3.76
Log $P_{o/w}$ (WLOGP)	4.00
Log $P_{o/w}$ (MLOGP)	3.52
Log $P_{o/w}$ (SILICOS-IT)	4.34
Consensus Log $P_{o/w}$	3.75

Water Solubility	
Log *S* (ESOL)	-4.88
Solubility	5.57e-03 mg/ml ; 1.32e-05 mol/l
Class	Moderately soluble
Log *S* (Ali)	-5.16
Solubility	2.96e-03 mg/ml ; 7.00e-06 mol/l
Class	Moderately soluble
Log *S* (SILICOS-IT)	-8.18
Solubility	2.82e-06 mg/ml ; 6.67e-09 mol/l
Class	Poorly soluble

Pharmacokinetics	
GI absorption	High
BBB permeant	No
P-gp substrate	No
CYP1A2 inhibitor	No
CYP2C19 inhibitor	Yes
CYP2C9 inhibitor	Yes
CYP2D6 inhibitor	No
CYP3A4 inhibitor	Yes
Log K_p (skin permeation)	-6.21 cm/s

Druglikeness	
Lipinski	Yes; 0 violation
Ghose	Yes
Veber	Yes
Egan	Yes
Muegge	Yes
Bioavailability Score	0.55

Medicinal Chemistry	
PAINS	0 alert
Brenk	0 alert
Leadlikeness	No; 2 violations: MW>350, XLOGP3>3.5
Synthetic accessibility	3.74

Molecule 9

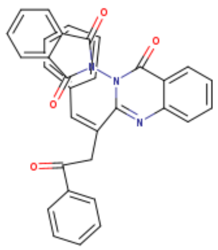

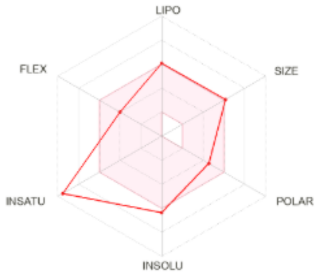

SMILES O=C(c1ccccc1)C/C(=C/c1ccccc1)/c1nc2ccccc2c(=O)n1N1C(=O)c2c(C1=O)cccc2

Physicochemical Properties	
Formula	C32H21N3O4
Molecular weight	511.53 g/mol
Num. heavy atoms	39
Num. arom. heavy atoms	28
Fraction Csp3	0.03
Num. rotatable bonds	6
Num. H-bond acceptors	5
Num. H-bond donors	0
Molar Refractivity	152.15
TPSA	89.34 Å²

Lipophilicity	
Log $P_{o/w}$ (iLOGP)	3.45
Log $P_{o/w}$ (XLOGP3)	5.11
Log $P_{o/w}$ (WLOGP)	4.65
Log $P_{o/w}$ (MLOGP)	4.56
Log $P_{o/w}$ (SILICOS-IT)	5.20
Consensus Log $P_{o/w}$	4.59

Water Solubility	
Log *S* (ESOL)	-6.37
Solubility	2.20e-04 mg/ml ; 4.30e-07 mol/l
Class	Poorly soluble
Log *S* (Ali)	-6.73
Solubility	9.53e-05 mg/ml ; 1.86e-07 mol/l
Class	Poorly soluble
Log *S* (SILICOS-IT)	-9.88
Solubility	6.77e-08 mg/ml ; 1.32e-10 mol/l
Class	Poorly soluble

Pharmacokinetics	
GI absorption	High
BBB permeant	No
P-gp substrate	No
CYP1A2 inhibitor	No
CYP2C19 inhibitor	Yes
CYP2C9 inhibitor	Yes
CYP2D6 inhibitor	No
CYP3A4 inhibitor	No
Log K_p (skin permeation)	-5.79 cm/s

Druglikeness	
Lipinski	No; 2 violations: MW>500, MLOGP>4.15
Ghose	No; 2 violations: MW>480, MR>130
Veber	Yes
Egan	Yes
Muegge	No; 1 violation: XLOGP3>5
Bioavailability Score	0.17

Medicinal Chemistry	
PAINS	0 alert
Brenk	1 alert: phthalimide
Leadlikeness	No; 2 violations: MW>350, XLOGP3>3.5
Synthetic accessibility	3.90

Molecule 10

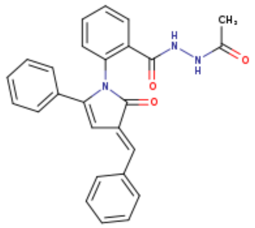

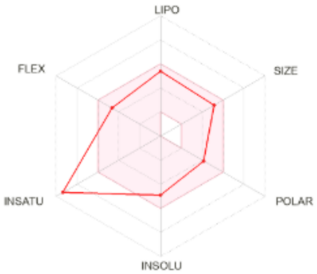

SMILES CC(=O)NNC(=O)c1ccccc1N1C(=O)/C(=C/c2ccccc2)/C=C1c1ccccc1

Physicochemical Properties	
Formula	C26H21N3O3
Molecular weight	423.46 g/mol
Num. heavy atoms	32
Num. arom. heavy atoms	18
Fraction Csp3	0.04
Num. rotatable bonds	7
Num. H-bond acceptors	3
Num. H-bond donors	2
Molar Refractivity	126.51
TPSA	78.51 Å²

Lipophilicity	
Log $P_{o/w}$ (iLOGP)	2.93
Log $P_{o/w}$ (XLOGP3)	3.93
Log $P_{o/w}$ (WLOGP)	3.45
Log $P_{o/w}$ (MLOGP)	3.35
Log $P_{o/w}$ (SILICOS-IT)	3.52
Consensus Log $P_{o/w}$	3.43

Water Solubility	
Log *S* (ESOL)	-4.90
Solubility	5.39e-03 mg/ml ; 1.27e-05 mol/l
Class	Moderately soluble
Log *S* (Ali)	-5.28
Solubility	2.23e-03 mg/ml ; 5.27e-06 mol/l
Class	Moderately soluble
Log *S* (SILICOS-IT)	-7.83
Solubility	6.19e-06 mg/ml ; 1.46e-08 mol/l
Class	Poorly soluble

Pharmacokinetics	
GI absorption	High
BBB permeant	Yes
P-gp substrate	No
CYP1A2 inhibitor	No
CYP2C19 inhibitor	Yes
CYP2C9 inhibitor	Yes
CYP2D6 inhibitor	No
CYP3A4 inhibitor	No
Log K_p (skin permeation)	-6.09 cm/s

Druglikeness	
Lipinski	Yes; 0 violation
Ghose	Yes
Veber	Yes
Egan	Yes
Muegge	Yes
Bioavailability Score	0.55

Medicinal Chemistry	
PAINS	1 alert: ene_five_het_C
Brenk	1 alert: michael_acceptor_1
Leadlikeness	No; 2 violations: MW>350, XLOGP3>3.5
Synthetic accessibility	3.94

Molecule 11

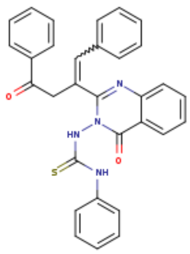

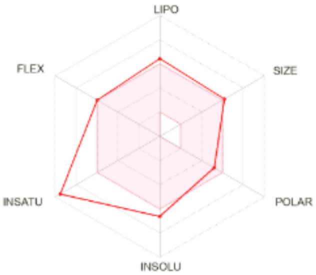

SMILES S=C(Nn1c(nc2c(c1=O)cccc2)/C(=C\c1ccccc1)/CC(=O)c1ccccc1)Nc1ccccc1

Physicochemical Properties	
Formula	C31H24N4O2S
Molecular weight	516.61 g/mol
Num. heavy atoms	38
Num. arom. heavy atoms	28
Fraction Csp3	0.03
Num. rotatable bonds	9
Num. H-bond acceptors	3
Num. H-bond donors	2
Molar Refractivity	157.56
TPSA	108.11 Å²

Lipophilicity	
Log $P_{o/w}$ (iLOGP)	3.88
Log $P_{o/w}$ (XLOGP3)	5.74
Log $P_{o/w}$ (WLOGP)	5.66
Log $P_{o/w}$ (MLOGP)	4.56
Log $P_{o/w}$ (SILICOS-IT)	6.07
Consensus Log $P_{o/w}$	5.18

Water Solubility	
Log *S* (ESOL)	-6.61
Solubility	1.27e-04 mg/ml ; 2.45e-07 mol/l
Class	Poorly soluble
Log *S* (Ali)	-7.78
Solubility	8.62e-06 mg/ml ; 1.67e-08 mol/l
Class	Poorly soluble
Log *S* (SILICOS-IT)	-10.46
Solubility	1.78e-08 mg/ml ; 3.45e-11 mol/l
Class	Insoluble

Pharmacokinetics	
GI absorption	Low
BBB permeant	No
P-gp substrate	No
CYP1A2 inhibitor	No
CYP2C19 inhibitor	Yes
CYP2C9 inhibitor	Yes
CYP2D6 inhibitor	No
CYP3A4 inhibitor	Yes
Log K_p (skin permeation)	-5.38 cm/s

Druglikeness	
Lipinski	No; 2 violations: MW>500, MLOGP>4.15
Ghose	No; 3 violations: MW>480, WLOGP>5.6, MR>130
Veber	Yes
Egan	Yes
Muegge	No; 1 violation: XLOGP3>5
Bioavailability Score	0.17

Medicinal Chemistry	
PAINS	0 alert
Brenk	1 alert: thiocarbonyl_group
Leadlikeness	No; 3 violations: MW>350, Rotors>7, XLOGP3>3.5
Synthetic accessibility	4.30

Molecule 12

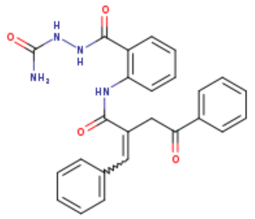

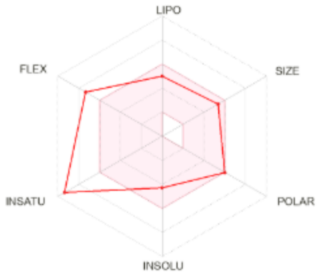

SMILES NC(=O)NNC(=O)c1ccccc1NC(=O)/C(=C\c1ccccc1)/CC(=O)c1ccccc1

Physicochemical Properties	
Formula	C25H22N4O4
Molecular weight	442.47 g/mol
Num. heavy atoms	33
Num. arom. heavy atoms	18
Fraction Csp3	0.04
Num. rotatable bonds	11
Num. H-bond acceptors	4
Num. H-bond donors	4
Molar Refractivity	123.98
TPSA	130.39 Å²

Lipophilicity	
Log $P_{o/w}$ (iLOGP)	2.20
Log $P_{o/w}$ (XLOGP3)	3.24
Log $P_{o/w}$ (WLOGP)	3.00
Log $P_{o/w}$ (MLOGP)	2.76
Log $P_{o/w}$ (SILICOS-IT)	2.44
Consensus Log $P_{o/w}$	2.73

Water Solubility	
Log *S* (ESOL)	-4.30
Solubility	2.21e-02 mg/ml ; 4.99e-05 mol/l
Class	Moderately soluble
Log *S* (Ali)	-5.65
Solubility	9.87e-04 mg/ml ; 2.23e-06 mol/l
Class	Moderately soluble
Log *S* (SILICOS-IT)	-7.40
Solubility	1.76e-05 mg/ml ; 3.98e-08 mol/l
Class	Poorly soluble

Pharmacokinetics	
GI absorption	High
BBB permeant	No
P-gp substrate	No
CYP1A2 inhibitor	No
CYP2C19 inhibitor	No
CYP2C9 inhibitor	Yes
CYP2D6 inhibitor	No
CYP3A4 inhibitor	No
Log K_p (skin permeation)	-6.70 cm/s

Druglikeness	
Lipinski	Yes; 0 violation
Ghose	Yes
Veber	No; 1 violation: Rotors>10
Egan	Yes
Muegge	Yes
Bioavailability Score	0.55

Medicinal Chemistry	
PAINS	0 alert
Brenk	1 alert: michael_acceptor_1
Leadlikeness	No; 2 violations: MW>350, Rotors>7
Synthetic accessibility	3.39

Molecule 13

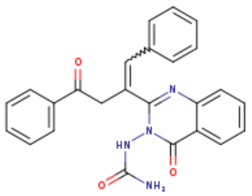

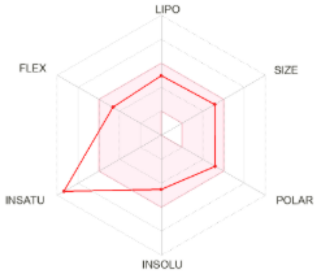

SMILES NC(=O)Nn1c(nc2c(c1=O)cccc2)/C(=C\c1ccccc1)/CC(=O)c1ccccc1

Physicochemical Properties	
Formula	C25H20N4O3
Molecular weight	424.45 g/mol
Num. heavy atoms	32
Num. arom. heavy atoms	22
Fraction Csp3	0.04
Num. rotatable bonds	7
Num. H-bond acceptors	4
Num. H-bond donors	2
Molar Refractivity	124.24
TPSA	107.08 Å²

Lipophilicity	
Log $P_{o/w}$ (iLOGP)	2.89
Log $P_{o/w}$ (XLOGP3)	3.18
Log $P_{o/w}$ (WLOGP)	3.53
Log $P_{o/w}$ (MLOGP)	3.32
Log $P_{o/w}$ (SILICOS-IT)	3.10
Consensus Log $P_{o/w}$	3.21

Water Solubility	
Log *S* (ESOL)	-4.52
Solubility	1.28e-02 mg/ml ; 3.01e-05 mol/l
Class	Moderately soluble
Log *S* (Ali)	-5.10
Solubility	3.37e-03 mg/ml ; 7.95e-06 mol/l
Class	Moderately soluble
Log *S* (SILICOS-IT)	-7.43
Solubility	1.58e-05 mg/ml ; 3.72e-08 mol/l
Class	Poorly soluble

Pharmacokinetics	
GI absorption	High
BBB permeant	No
P-gp substrate	No
CYP1A2 inhibitor	No
CYP2C19 inhibitor	Yes
CYP2C9 inhibitor	Yes
CYP2D6 inhibitor	No
CYP3A4 inhibitor	No
Log K_p (skin permeation)	-6.63 cm/s

Druglikeness	
Lipinski	Yes; 0 violation
Ghose	Yes
Veber	Yes
Egan	Yes
Muegge	Yes
Bioavailability Score	0.55

Medicinal Chemistry	
PAINS	0 alert
Brenk	0 alert
Leadlikeness	No; 1 violation: MW>350
Synthetic accessibility	3.77

Molecule 14

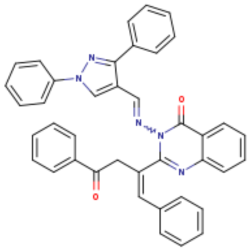

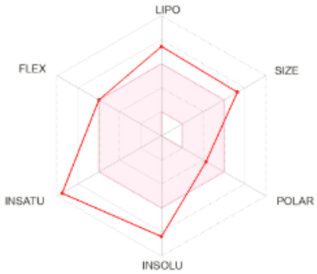

SMILES O=C(c1ccccc1)C/C(=C/c1ccccc1)/c1nc2ccccc2c(=O)n1/N=C\c1cn(nc1c1ccccc1)c1ccccc1

Physicochemical Properties	
Formula	C40H29N5O2
Molecular weight	611.69 g/mol
Num. heavy atoms	47
Num. arom. heavy atoms	39
Fraction Csp3	0.03
Num. rotatable bonds	9
Num. H-bond acceptors	5
Num. H-bond donors	0
Molar Refractivity	187.89
TPSA	82.14 Å²

Lipophilicity	
Log $P_{o/w}$ (iLOGP)	4.94
Log $P_{o/w}$ (XLOGP3)	7.51
Log $P_{o/w}$ (WLOGP)	7.84
Log $P_{o/w}$ (MLOGP)	5.44
Log $P_{o/w}$ (SILICOS-IT)	7.62
Consensus Log $P_{o/w}$	6.67

Water Solubility	
Log *S* (ESOL)	-8.38
Solubility	2.53e-06 mg/ml ; 4.13e-09 mol/l
Class	Poorly soluble
Log *S* (Ali)	-9.07
Solubility	5.22e-07 mg/ml ; 8.53e-10 mol/l
Class	Poorly soluble
Log *S* (SILICOS-IT)	-13.23
Solubility	3.64e-11 mg/ml ; 5.94e-14 mol/l
Class	Insoluble

Pharmacokinetics	
GI absorption	Low
BBB permeant	No
P-gp substrate	No
CYP1A2 inhibitor	No
CYP2C19 inhibitor	No
CYP2C9 inhibitor	No
CYP2D6 inhibitor	No
CYP3A4 inhibitor	No
Log K_p (skin permeation)	-4.70 cm/s

Druglikeness	
Lipinski	No; 2 violations: MW>500, MLOGP>4.15
Ghose	No; 4 violations: MW>480, WLOGP>5.6, MR>130, #atoms>70
Veber	Yes
Egan	No; 1 violation: WLOGP>5.88
Muegge	No; 2 violations: MW>600, XLOGP3>5
Bioavailability Score	0.17

Medicinal Chemistry	
PAINS	0 alert
Brenk	1 alert: imine_1
Leadlikeness	No; 3 violations: MW>350, Rotors>7, XLOGP3>3.5
Synthetic accessibility	4.74

Molecule 15

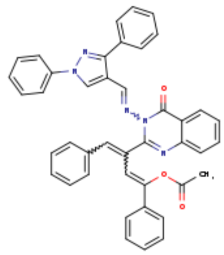

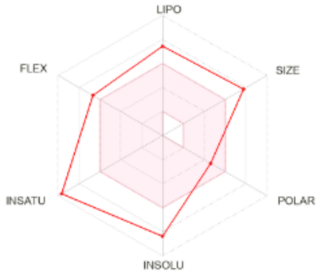

SMILES	CC(=O)O/C(=C\C(=C\c1ccccc1)\c1nc2ccccc2c(=O)n1/N=C\c1cn(nc1c1ccccc1)c1ccccc1)/c1ccccc1

Physicochemical Properties	
Formula	C42H31N5O3
Molecular weight	653.73 g/mol
Num. heavy atoms	50
Num. arom. heavy atoms	39
Fraction Csp3	0.02
Num. rotatable bonds	10
Num. H-bond acceptors	6
Num. H-bond donors	0
Molar Refractivity	198.68
TPSA	91.37 Å²

Lipophilicity	
Log $P_{o/w}$ (iLOGP)	4.94
Log $P_{o/w}$ (XLOGP3)	7.51
Log $P_{o/w}$ (WLOGP)	8.17
Log $P_{o/w}$ (MLOGP)	5.44
Log $P_{o/w}$ (SILICOS-IT)	7.62
Consensus Log $P_{o/w}$	6.67

Water Solubility	
Log *S* (ESOL)	-8.38
Solubility	2.53e-06 mg/ml ; 4.13e-09 mol/l
Class	Poorly soluble
Log *S* (Ali)	-9.07
Solubility	5.22e-07 mg/ml ; 8.53e-10 mol/l
Class	Poorly soluble
Log *S* (SILICOS-IT)	-13.23
Solubility	3.64e-11 mg/ml ; 5.94e-14 mol/l
Class	Insoluble

Pharmacokinetics	
GI absorption	Low
BBB permeant	No
P-gp substrate	No
CYP1A2 inhibitor	No
CYP2C19 inhibitor	No
CYP2C9 inhibitor	No
CYP2D6 inhibitor	No
CYP3A4 inhibitor	No
Log K_p (skin permeation)	-4.70 cm/s

Druglikeness	
Lipinski	No; 2 violations: MW>500, MLOGP>4.15
Ghose	No; 4 violations: MW>480, WLOGP>5.6, MR>130, #atoms>70
Veber	Yes
Egan	No; 1 violation: WLOGP>5.88
Muegge	No; 2 violations: MW>600, XLOGP3>5
Bioavailability Score	0.17

Medicinal Chemistry	
PAINS	0 alert
Brenk	1 alert: imine_1
Leadlikeness	No; 3 violations: MW>350, Rotors>7, XLOGP3>3.5
Synthetic accessibility	4.74
